# Lamin C is required to establish genome organization after mitosis

**DOI:** 10.1186/s13059-021-02516-7

**Published:** 2021-11-15

**Authors:** Xianrong Wong, Victoria E. Hoskins, Ashley J. Melendez-Perez, Jennifer C. Harr, Molly Gordon, Karen L. Reddy

**Affiliations:** 1 Department of Biological Chemistry, Center for Epigenetics, School of Medicine, Johns Hopkins University, Baltimore, MD 21205 USA; 2 Current Address: Laboratory of Developmental and Regenerative Biology, A*STAR Skin Research Labs, Agency for Science, Technology and Research (A*STAR), Immunos, Singapore, 138648 Singapore; 3 McKusick-Nathans Department of Genetic Medicine, Johns Hopkins University School of Medicine, Baltimore, MD 21205 USA; 4 Department of Biological Sciences, St. Mary’s University, San Antonio, TX 78228 USA; 5 Department of Cell Biology, Center for Cell Dynamics, School of Medicine, Johns Hopkins University, Baltimore, MD 21205 USA; 6 Sidney Kimmel Comprehensive Cancer Center, School of Medicine, Johns Hopkins University, Baltimore, MD 21205 USA

## Abstract

**Background:**

The dynamic 3D organization of the genome is central to gene regulation and development. The nuclear lamina influences genome organization through the tethering of lamina-associated domains (LADs) to the nuclear periphery. Evidence suggests that lamins A and C are the predominant lamins involved in the peripheral association of LADs, potentially serving different roles.

**Results:**

Here, we examine chromosome architecture in mouse cells in which lamin A or lamin C are downregulated. We find that lamin C, and not lamin A, is required for the 3D organization of LADs and overall chromosome organization. Striking differences in localization are present as cells exit mitosis and persist through early G1 and are linked to differential phosphorylation. Whereas lamin A associates with the nascent nuclear envelope (NE) during telophase, lamin C remains in the interior, surrounding globular LAD aggregates enriched on euchromatic regions. Lamin C association with the NE is delayed until several hours into G1 and correlates temporally and spatially with the post-mitotic NE association of LADs. Post-mitotic LAD association with the NE, and global 3D genome organization, is perturbed only in cells depleted of lamin C, and not lamin A.

**Conclusions:**

Lamin C regulates LAD dynamics during exit from mitosis and is a key regulator of genome organization in mammalian cells. This reveals an unexpectedly central role for lamin C in genome organization, including inter-chromosomal LAD-LAD segregation and LAD scaffolding at the NE, raising intriguing questions about the individual and overlapping roles of lamin A/C in cellular function and disease.

**Supplementary Information:**

The online version contains supplementary material available at 10.1186/s13059-021-02516-7.

## Introduction

Lamins, encoded by *LMNA*, *LMNB1*, and *LMNB2*, form networks of nuclear intermediate filaments that are major components of the nucleoskeleton. Lamin filaments interact with key partners including most nuclear membrane proteins and linked to the cytoplasm through LINC (Linker of Nucleoskeleton and Cytoskeleton) to form nuclear lamina networks that determine nuclear mechanics, modulate signaling, and dynamically organize the genome [1–6]. Lamina networks interact with large regions of transcriptionally silent heterochromatin in each cell type to customize the 3D configurations of individual chromosomes with respect to the nuclear envelope (NE). These silent heterochromatin regions, identified operationally as lamina-associated domains (LADs), correspond to the “B” compartment identified via HiC and related chromatin mapping strategies [7–9]. Chromatin association with the lamina, and its opposite (dynamic release as active “non-LAD” or A-compartment chromatin) are particularly important for developmentally regulated genes needed to create or maintain cell-specific identity [8, 10–12]. “Silent” histone modifications including H3 lysine 9 methylation (H3K9me2/3) and H3 lysine 27 trimethylation (H3K27me3) are key components of LAD organization [6, 13–16]. We and others have previously found that A-type lamins, encoded by *LMNA*, establish and/or maintain interphase LAD configuration in some cell types [6, 17, 18]. In recent years, there have been further advances in understanding how LADs, chromatin looping, epigenetic modifications, and liquid-liquid phase transitions of chromatin during interphase are interrelated [2, 3, 5–7, 13–16, 19–24].

In contrast, the mechanisms by which nuclear 3D structural information is faithfully transmitted and re-established during cell division are of significance and remain largely unknown. During entry into mitosis, interphase spatial genome and NE organization are lost as chromosomes condense and the NE and nuclear lamina networks disassemble, yet global chromosome and 3D-genome organization is re-established in the next interphase [8, 9, 16, 25–31]. As cells enter anaphase, both super-resolution imaging and single cell Hi-C (a genome-wide method to detect chromatin contacts) showed that LAD regions of chromosomes begin to self-aggregate as globular “compartments” prior to nuclear lamina formation [9]. These LAD aggregates slowly make their way to the nascent NE during early G1 and ultimately “spread” across the lamina as they approach and interact with the nuclear periphery [9].

One key protein in interphase LAD organization and NE function are the A-type lamins. Alternative mRNA splicing of *LMNA* produces two main somatic isoforms, lamin A and lamin C, the first 566 or 568 residues of which are identical in human and mice, respectively [32]. Lamin C has six unique C-terminal residues, whereas lamin A has an extended tail domain that undergoes four post-translational modification steps to achieve its final mature length of 646 or 647 residues, in humans and mice respectively [33]. Until recently lamin A and lamin C were assumed to be mostly functionally redundant. Super-resolution microscopy showed lamins A, C, B1, and B2 form separate but structurally inter-dependent filaments such that removing any one (e.g., lamin C) affects the distribution and geography of the other three [34–36]. An additional study found that the lamins may form concentric rings around the nuclear periphery, with A-type lamins positioned interior to lamin B [37]. These data strongly imply that these isotypes might have different functions and this is supported by other data as well. For instance, lamin A specifically confers mechanical stiffness that determines when different white blood cell lineages exit the bone marrow [38]. In CNS neurons, lamin C is the predominant A-type lamin isoform expressed due to the selective downregulation of the prelamin A transcript by miR-9 [39–41]. Studies in mice that express only one A-type lamin (mature lamin A, or lamin C) also highlight potential functional differences. Mice expressing mature lamin A (no lamin C) have few if any overt phenotypes with the exception of misshapen nuclei, whereas mice that only express only lamin C (no lamin A) have longer lifespans, are mildly obese, and are predisposed to cancer [42–44].

Intriguingly, both A-type lamins and the Lamin B Receptor (LBR; a nuclear membrane protein) are essential molecular tethers for heterochromatin at the NE [18]. LBR is especially important in early development (when lamin A/C expression is low), while A-type lamins are prominent later in development, perhaps explaining why lamins are thought to be dispensable for proliferation and differentiation of mouse embryonic stem cells (mESC) [45–47]. It remains unclear if lamins are necessary for robust LAD organization in mESC [45, 46]. Previously, we showed in fibroblasts, which are more terminally differentiated and where LBR is only minimally expressed, interphase LAD organization is disrupted by depleting both A-type lamins (A and C), but not lamin A alone [6]. Taken together, these data suggest lamin C might be required to tether LADs at the NE in cells lacking LBR. Therefore, in this study, we examine the role of lamin C in LAD organization by examining LAD and chromosome configuration in cells specifically depleted in either lamin C or lamin A. Our data strongly support the hypothesis that lamin C is uniquely required for large-scale chromosome organization. Our results provide insight into the mechanisms of 3D genome organization during interphase and its dynamic re-establishment after mitosis in cycling cells.

## Results

### LAD proximity to the nuclear envelope is maintained by Lamin C

To test our hypothesis, we developed short hairpin RNAs (shRNAS) that specifically downregulate lamin A (shA) or lamin C (shC). We also used shRNAs that downregulate both lamins A and C (shAC) [6], or lamin B1 (shB1). To characterize the efficacy of lamin depletion and global effects on mouse embryonic fibroblasts (MEFs), we assayed for the presence of lamin isotypes after 4 days and monitored growth of cells during the same time-frame (Additional file 1:Fig. S1). Each shRNA specifically targeted its own lamin, as shown by western analysis (Additional file 1:Fig. S1A). shC, shB1, and shlacZ (control) all grew at the same rate, while shA and shAC reduced growth rates, suggesting a unique role for lamin A in cell growth and cycling (Fig S1B). To try to assess if these isotypes had effects on LAD organization, particularly at specific regions, we performed a global DamID-seq analysis of LAD positioning (using Dam-lamin B1) in shRNA-treated MEFs. Surprisingly, these analyses revealed no significant differences in cells depleted of both lamin A and lamin C compared to wild-type cells (shAC; Additional file 1:Fig. S2, S3). While this was initially surprising, especially given our earlier findings that lamin A/C is required to organize regions to the lamina, we realize that an inherent limitation of DamID and related techniques is that data are aggregated from millions of cells, potentially obscuring true differences that would be detectable at the level of individual cells [6, 8, 48]. Detailed analysis of LAD boundaries (region of transition from NE-associated to NE non-associated), which normally are quite sharp, showed that LAD boundaries also remained intact under all four conditions (control, shAC, shA, shB1, or shC); genome-wide comparisons between log_2_ ratios of DamID-seq signals were virtually indistinguishable (Additional file 1:Fig. S2A, B, S3), and genome-wide bioinformatically defined LADs for all three downregulated conditions showed preservation of WT LADs (>90% by base coverage) (Additional file 1:Fig. S2C).

**Fig. 1:**
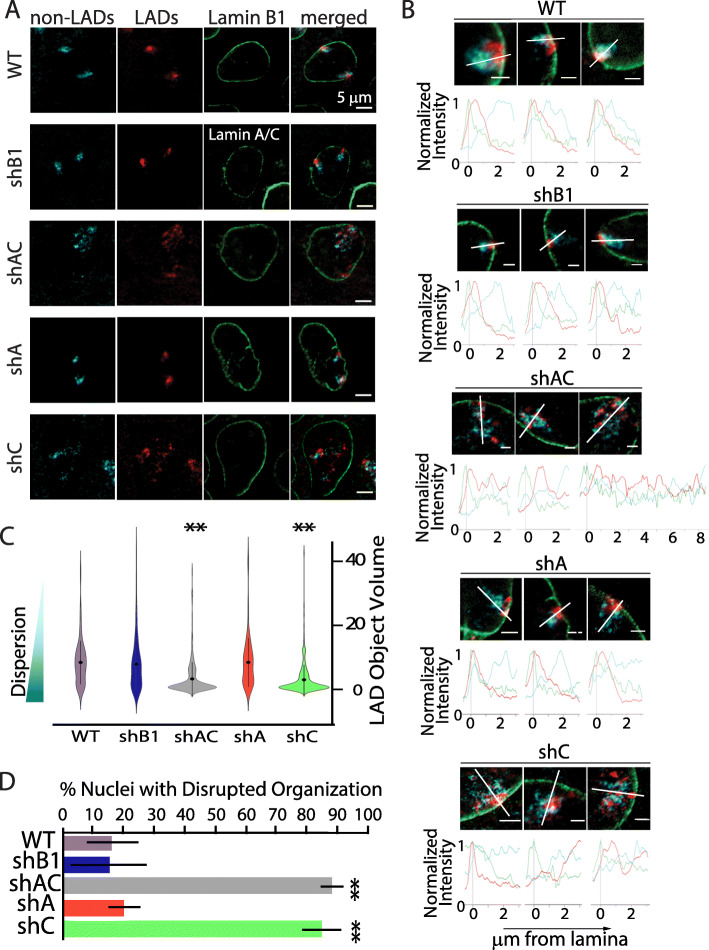
Specific knockdown of lamin C disrupts LAD aggregation and localization at the single cell level. **A** Representative images showing the organization of chromosome 11 with nonLADs in cyan, LADs in red, and lamin B1 (or lamin A/C for shB1) in green. ShA representative image shows one territory that is attached in the medial plane and one territory that is attached at the top of the nucleus where a portion of the LAD aggregate hangs down into the medial plane. **B** Representative images of chromosome conformation paints to chromosome nonLADS (cyan), LADs (red), and lamin B1 (green) in primary MEFs. Images were chosen to represent the spectrum of phenotypes for each knockdown. Normalized fluorescence intensity histogram plots, from nuclear lamina (0μm) to 3μm into the nucleus, were plotted for all chromosome 11 territories (except territory 3 of shAC which required a longer measurement) to display the extent of LAD signals. The line each plot travels through is represented by a white line. Scale bar = 2μm. **C** Violin plots showing the distribution of the volumes of segmented objects based on LAD signals, with smaller objects indicating greater dispersion of LADs (n>50 territories per condition). ShAC (gray) and shC (green) show significant LAD dispersion. Statistically significant changes from wild-type dispersion were noted in shAC and shC conditions (marked with **, *p*<0.001). **D** Quantification of nuclei with disrupted organization for each knockdown condition. Error bars represent 1 standard deviation. ** indicates statistically significant differences in percent disrupted nuclei when compared to WT control (*t*-test *p* value <0.001; *n*>200 nuclei per condition)

To observe LAD organization in single cells, we used 3D-immunoFISH to examine downregulated MEFs. We highlighted the NE in green using antibodies to lamin B1 (or lamin A/C in the case of shB1), and the LAD and non-LAD regions of chromosome 11 were “painted” red and cyan, respectively, using oligonucleotide-based Chromosome Conformation Paints (CCP) [8, 9]. As previously shown, in control nuclei, each chromosome occupied its own territory (Fig. 1A top row), with LADs clustered together near the NE and non-LADs extending into the nucleoplasm [8, 9]. This organization was grossly disrupted in cells depleted of both lamin A and lamin C (shAC); LADs failed to co-associate, with many LADs mislocalized to the nucleoplasm, and non-LADs dispersed to occupy a territory considerably larger than controls (Fig. 1A). This finding is consistent with another study that showed chromosome territory expansion upon removal of lamin A/C [17].

We next asked what the role and contribution of each individual lamin isotype was to this organization. We observed no gross perturbations of genome organization after loss of lamin B1. Furthermore, loss of lamin A had no discernible effect on LAD organization, even though loss of this isotype displayed delayed growth. Strikingly, loss of lamin C was sufficient to fully recapitulate the gross disruption of chromosome 11 organization seen with loss of both isotypes (Fig. 1A, shAC vs. shC). To better visualize and describe the observed perturbations, we plotted signal intensities from individual nuclei across lines drawn through the medial plane of each chromosome territory, as previously described [9] (Figs. 1B, S4, S5, S6, S7 and S8). This analysis confirmed the normal positioning of LADs and non-LADs in control nuclei (Figs. 1A, B, S4), lamin B-depleted nuclei (Figs. 1A, B, S5), and lamin A-depleted nuclei (Figs 1A, B and S7) and confirmed the broadening of the distribution of the LAD signal intensity and the overall dispersion of LADs away from the NE in lamin C-depleted cells (Figs. 1A, B, S8); however, because of the severity of the phenotype and the inability to discriminate between individual deranged chromosomes, we could not reliably average these data.

The line scan analyses show that LADs lose association with the NE. To more accurately capture the loss of LAD:LAD interactions and compartmentalization, we computationally determined the relative extent of LAD dispersion across the conditions using object identification to measure “LAD” sizes (illustrated in Additional file 1:Fig. S9). The rationale is that LAD:LAD interactions lead to an intact and larger LAD sub-chromosomal domain territory and will be recognized as one (or few) objects by segmentation, while loss of LAD:LAD cohesion will result in more and smaller object volumes (see Additional file 1:Fig. S9). LAD signals were segmented into objects irrespective of size and the distribution of these were plotted (Figs. 1C, S9). Strikingly, while the LAD object distributions appear similar in control nuclei, lamin B-depleted nuclei, and lamin A-depleted nuclei, nuclei depleted of lamin A/C or lamin C alone showed a statistically significant degree of LAD dispersion, with the distribution of LAD objects skewed towards smaller volumes (Fig. 1C, *p* < 0.001 in both cases).

We note that while both the abovementioned analyses have their own merits, signal intensity plotting (line scans) and LAD object volumes measure different aspects of genome organizational disruption with respect to LADs (peripheral positioning and dispersion, respectively). Therefore, to account for all disrupted cells, we counted the percentage of cells with visually disrupted genome organization in each population (Fig. 1D). Nuclei were considered disrupted if one (or both) chromosomes had LADs that were either dispersed (not aggregated or loss of LAD:LAD cohesion) and/or gross loss of NE-association. These were scored by two independent observers (blinded). By this metric, genome organization was disrupted in 16% of wildtype control nuclei (Fig. 1D). Cells depleted of lamin A showed a similar baseline (18%; Fig. 1D). We note that these cells are unsynchronized, so any alterations in LAD organization due to cell cycle stage are encompassed in this baseline data. In contrast, genome organization was disrupted in 85% of lamin C-depleted cells (*p*<0.001; Figs. 1D, S8) and 88% of shAC cells (*p*<0.001; Figs. 1D and S6). One possibility that lamin C was uniquely required for cell cycle progression is unlikely since the doubling times for shC matched control cells were the same (Additional file 1:Fig. S1, shLacZ vs. shC). Overall, these results suggest that lamin C is required for LAD self-association (LAD:LAD cohesion), LAD retention near the NE, and overall compaction of the chromosome territory (including non-LAD regions).

### Lamin C (not lamin A or lamin B1) is required to maintain LAD association with the NE

**Fig. 2:**
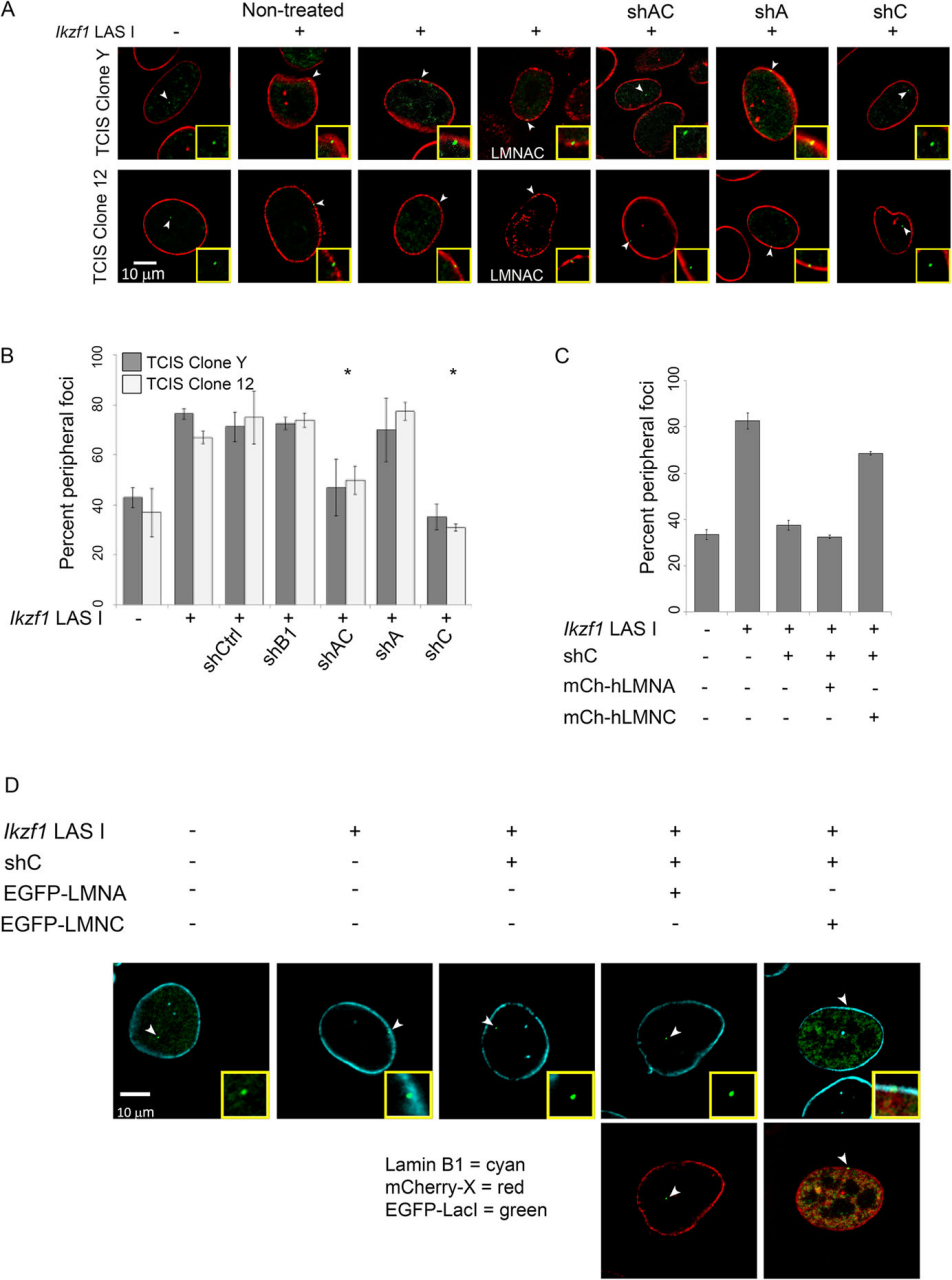
Specific knockdown of lamin C causes LASes to delocalize from the nuclear periphery and can be rescued by re-expression of lamin C. **A** Representative images showing the disposition of EGFP-LacI bound *lacO* arrays (arrowheads, green) and lamin B1 (red; lamin A/C for shB) in the TCIS clones Y (top) and 12 (bottom) pre- and post-integration of the *Ikzf* LASI, which drives lamina-association, and indicated lamin isotype depletion. The inset shows 300× magnification. **B** Quantitation of peripheral association was determined by overlap of EGFP-LacI bound foci and lamin B1 (or lamin A/C for shB1; *n* ≥ 50). Error bars indicate SD and statistically significant differences in peripheral association of treatment conditions when compared to shCtrl (*p* ≤ 0.001) are indicated by asterisks. **C** Quantitation of peripheral association in rescue experiments was determined as in **B**. **D** Representative images showing the disposition of *lacO* arrays in rescue experiments as in **A**

To independently evaluate the role of lamin C, we used cells bearing a single “TCIS” LAD. TCIS (Tagged Chromosomal Insertion Site) is comprised of 256 tandem copies of the *lacO* sequence (allowing visualization upon expression of eGFP-LacI) and a modified RMCE (recombination-mediated cassette exchange) “cassette,” allowing for insertion of ectopic sequences. We previously showed, using this system, that a single segment of DNA (lamina-associated sequence or LAS) can redirect the TCIS locus to the nuclear lamina. To test if lamin C is required for localization of a de novo LAD, we used two independent MEF clones bearing one of these TCIS-LAS, specifically, the *Ikzf1* (Ikaros zinc finger protein) D6 Lamin Associated Segment (*Ikzf1 LAS I*), as previously described [6]. When this LAS is introduced into the clonal TCIS MEF lines (clone Y or clone 12, Fig. 2A, B), the *lacO* locus was NE-associated in 75–80% of nuclei, compared to 40% of nuclei with a no LAS, when visualized by 3D-immunoFISH (Fig. 2A, B) and quantified by co-localization with lamin B1 (or lamin A/C for shB1) (Fig. 2A, B).

We used this TCIS-LAS (*Ikzf1 LAS I*) which had high association with the NE to monitor and quantify LAD organization in MEFs depleted of lamin isotypes. Removing lamin B1 alone (shB1) or lamin A alone (shA) had no significant effect on *lacO* array disposition (Fig. 2A, B), whereas NE-association was reduced significantly in cells depleted of both A and C (shAC; Fig. 2A, B; *p*<0.001), or lamin C alone (shC; Fig. 2A, B; *p*<0.001). Without lamin C, the percentage of NE-associated *Ikzf1 LAS I lacO* loci was reduced to the background seen with LAS-less *lacO* array loci (Fig. 2A, B). These data are consistent with our previous finding that acute shRNA-mediated removal of lamin A was insufficient to perturb LAS NE-association [6]. Given that shC treated cells showed loss of LAD organization, we next verified that this phenotype could be rescued by expression of mCherry-tagged lamin C, but not other lamin variants. We stably expressed either mCherry-tagged human lamin A or lamin C in shC-treated MEFs. Importantly, these constructs were not targeted by our murine-specific shRNA. *TCIS-LAS* localization at the NE was fully restored by mCherry-lamin C, and not by mCherry-lamin A (Fig. 2C, D). These results independently supported the hypothesis that lamin C, in contrast to lamin A and lamin B1, is required to maintain LAD association with the NE and nuclear lamina and that simply depleting levels of A-type lamins is not sufficient to cause de-localization.

### Lamin C is nucleoplasmic during telophase and early G1-phase and is significantly delayed in its association with the reforming NE

**Fig. 3:**
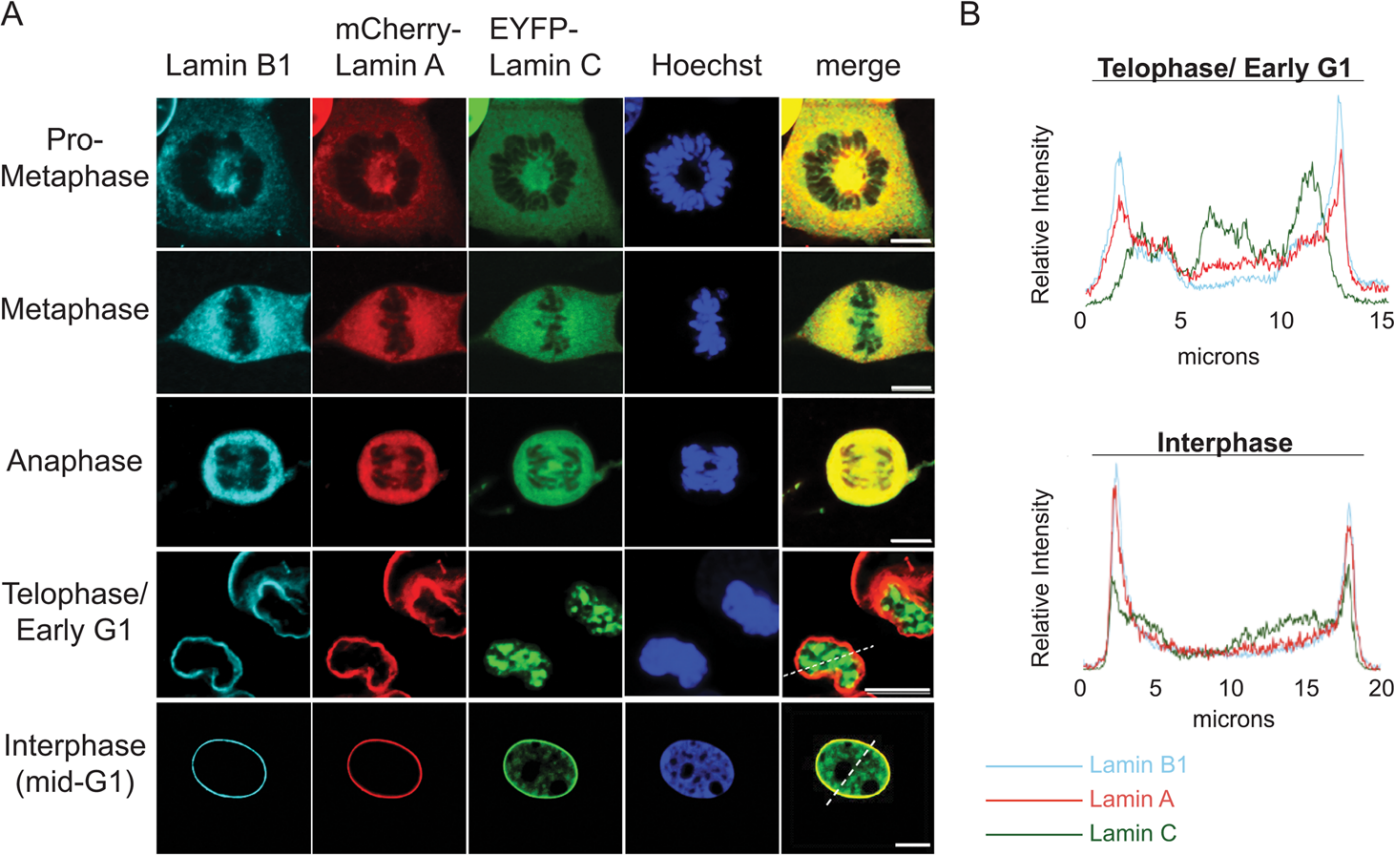
Lamin C shows delayed localization to the nuclear periphery during nuclear envelope reformation. **A** Representative images of lamin B1 (cyan), lamin A (red), lamin C (green), and chromatin (blue) in different stages of the cell cycle. Merged images show lamins A and C localization. Dotted lines on the merged images for telophase/early G1 and G1 indicate the segment used for line scan displays shown in **B**. **B** shows representative plots of the normalized intensities of lamin B1 (cyan), lamin A (red), and lamin C (green) along the dotted lines (from left to right) as shown in the merged images in **A** for the telophase/early G1 transition stage of the cell cycle and the mid-G1 (interphase) stage. Scale bar is 10μm

Our results thus far show that lamin C is important for normal interphase LAD configuration in MEFs (Figs. 1, 2). After mitosis, the nuclear lamina itself must be rebuilt and organized. The major consensus is that B-type lamins associate with the nascent NE prior to A-type lamins, and evidence of the differential dynamics of NE incorporation for lamin A versus lamin C are conflicting. In support of lamin C incorporating at the NE after lamin A, a study using injected recombinant proteins showed lamin A exhibited much faster lamina incorporation kinetics (20min) compared to lamin C (180 min) [49]. Even at 180min post-injection, nucleoplasmic lamin C foci were still evident. However, in that study, the incorporation of lamin C into the lamina was accelerated upon co-injection with lamin A, suggesting some cross regulation, in agreement with another study suggesting lamin C localization is dependent on lamin A [50]. A drawback of this study is that the normal regulation of both A/C ratios and post-translational modifications (PTM) are lacking and other studies have correct localization of lamin C to the NE in the absence of lamin A [34]. In support of lamin A and C arriving at the NE at the same time, a study using lamin A and lamin C over-expressed individually found that both A-type lamins post-mitosis had similar kinetics of NE localization [51]. Thus, there are conflicting data on the timing and recruitment of the A-type lamins to the NE. Of interest, interphase LAD and chromatin compartment organization is also ablated during mitosis and must also be re-built in the next G1, with overall chromosome positions and chromatin domains faithfully reinstated [8, 9, 16, 25, 31]. Yet, the pathway(s) and the mechanisms by which LADs reorganize and re-associate with the nuclear lamina after mitosis are still not understood. In order to understand the role that lamin C might play in this process and given the conflicting data regarding timing of lamin C incorporation to the lamina, we first sought to evaluate post-mitotic lamin isotype NE incorporation dynamics in our MEFs. We imaged lamins A, C, and B1 during exit from mitosis using a specific lamin B1 antibody in MEFs co-expressing mCherry-lamin A and eYFP-lamin C (Fig. 3). Localization of each lamin isotype was measured by fluorescence intensity histograms for a minimum of 20 nuclei along a line drawn through the medial plane of the nuclear volume as determined by Hoechst signal (Fig. 3). We found that all isotypes tested, lamins A, C, and B1 concentrate at the NE during interphase (Fig. 3 and Additional file 1:Fig. S10), with lamin C and, to a lesser degree, lamin A also localizing diffusely in the nucleoplasm (Fig. 3 and Additional file 1:Fig. S10). Such nucleoplasmic localization was not unexpected given previous reports describing a “nuclear veil” of lamin A/C [52–54]. However, during telophase, when lamin A and lamin B1 are already colocalized at the nascent NE, lamin C was solely nucleoplasmic, with no detectable concentration at the NE (Fig. 3 and Additional file 1:Fig. S11). This telophase association of lamin A with the NE has recently been confirmed in a high throughput imaging screen with a lamin A specific antibody [55]. We verified these results using antibodies specific for each of the lamin isoforms (lamin B1, lamin A, and lamin C; Additional file 1:Fig. S12). These results were highly reproducible from cell to cell (Figs. 3, S11, S12), with lamin C persisting predominantly in the nuclear interior well into early G1, and then gradually incorporating into the nuclear lamina.

This delayed arrival of lamin C at the NE during early G1-phase was reminiscent of our previous finding relating to the timing of LAD re-incorporation at the nuclear lamina after mitosis [9]. LADs themselves form nucleoplasmic intra-chromosomal LAD:LAD agglomerations during telophase and early-G1, and only later re-associate with the NE; as cells progress further into G1-phase, more LADs become NE-proximal and flattened against the lamina, with a subset of LADs remaining in the nuclear interior for up to 3 or 4 h, well into G1-phase, quite reminiscent of the timing we noted for lamin C NE incorporation [9, 16]. This led us to question whether lamin C might colocalize with LADs at the end of mitosis and into early G1. To test this, we used a LAD-tracer system to fluorescently localize all endogenous LADs in living cells (MEFs) that also expressed eYFP-lamin C [9, 16, 56]. The LAD-tracer system relies on the expression of two constructs. First, a construct expressing Dam-lamin B1 enables methylation of DNA at adenine residues in proximity to the nuclear periphery (i.e., marks LADs with ^me^A), but has two additional domains that strictly control its expression: a destabilization domain (DD) that causes degradation in the absence of the shield ligand and a Cdc10 dependent transcript 1 (CDT) regulatory domain restricts its expression to G1 [9, 16, 56, 57]. The second construct, the LAD-tracer, is a modified mCherry-tagged version of the previously described m6A-tracer [16, 56] that binds the ^me^A, the modification generated by Dam-lamin B1, thus marking LADs with mCherry. The LAD-tracer is expressed throughout the cell cycle and identifies ^me^A-modified DNA (LADs) in all phases in cells where both constructs are expressed at appropriate levels.

**Fig. 4:**
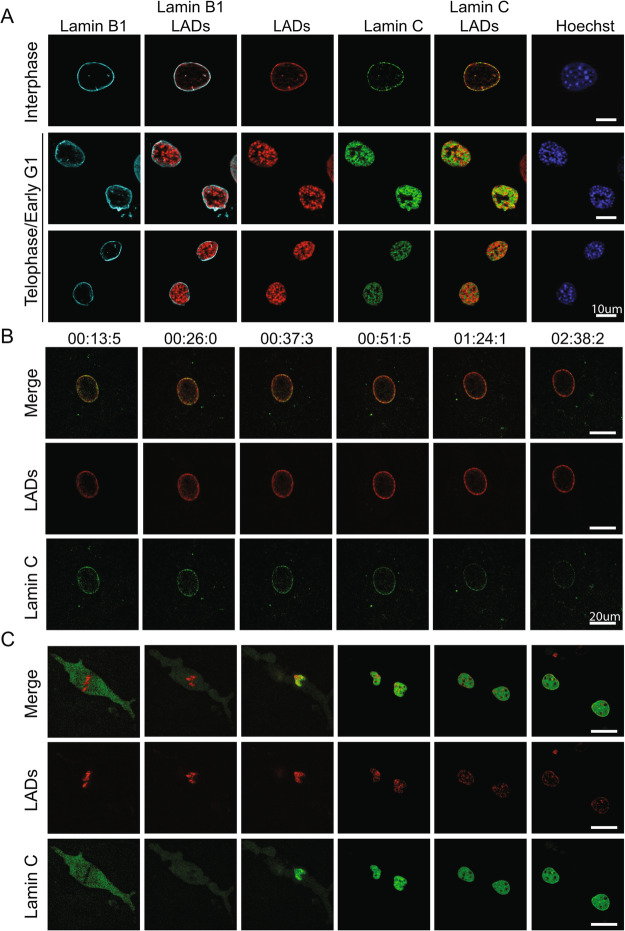
Lamin C and LADs do not colocalize but resolve to the lamina at the same time during G1. **A** Representative images of interphase and early G1 nuclei of anti-lamin B1 (cyan), LAD-tracer (red), lamin C (green), and Hoechst 33342 (blue). **B** Still images from time lapse movie 3 of LADs (red) and EGFP-LmnC (green) during interphase collected in parallel to panel B. Scale Bar is 20μm. **C** Still images from time lapse movie 1 of LADs (red), and EGFP-LmnC (green) during mitosis. Scale bar is 20μm. Images were chosen to exemplify certain stages (metaphase, anaphase, telophase, early G1, partially resolved, fully resolved)

As expected, eYFP-lamin C and LAD-tracer signals were both at the NE in most interphase cells (mid-G1-G2; Figs. 4A, B, S13, S14, movie 1, and movie 2). However, during telophase and early-G1 we were surprised to find that lamin C and LADs occupied distinct nuclear volumes with minimal or no co-localization (Figs. 4A, C and S13, S15, S16). Despite their apparent separation, time-lapse movies showed that eYFP-lamin C and LAD aggregates had coincident arrival to and integration with the nuclear lamina (Figs. 4C, S13, movie 3, and movie 4). These results suggest lamin C functions distal to LADs to influence their organization.

### Ser22 phosphorylation is preserved on nucleoplasmic lamin C upon mitotic exit

Our finding that lamin C preferentially colocalizes with euchromatic regions at mitotic exit is particularly intriguing given a recent study that found that A-type lamins, particularly lamin C, in the nucleoplasm in interphase preferentially interact with enhancers and promoters of active genes [54]. The lamins bound to these euchromatic regions remain phosphorylated on Serine 22 (a known mitotic PTM) even after mitotic exit, suggesting that these interactions were set up during mitosis and persist into interphase. Previous studies also found nucleoplasmic lamins bound to euchromatic regions, which relied on the nucleoplasmic LAP2 (lamina associated peptide 2) isoform lap2ɑ [58, 59]. How the dependence of nucleoplasmic A-type lamins on LAP2ɑ is manifest has not been explored, but could be the result of PTM regulation on A-type lamins.

**Fig. 5:**
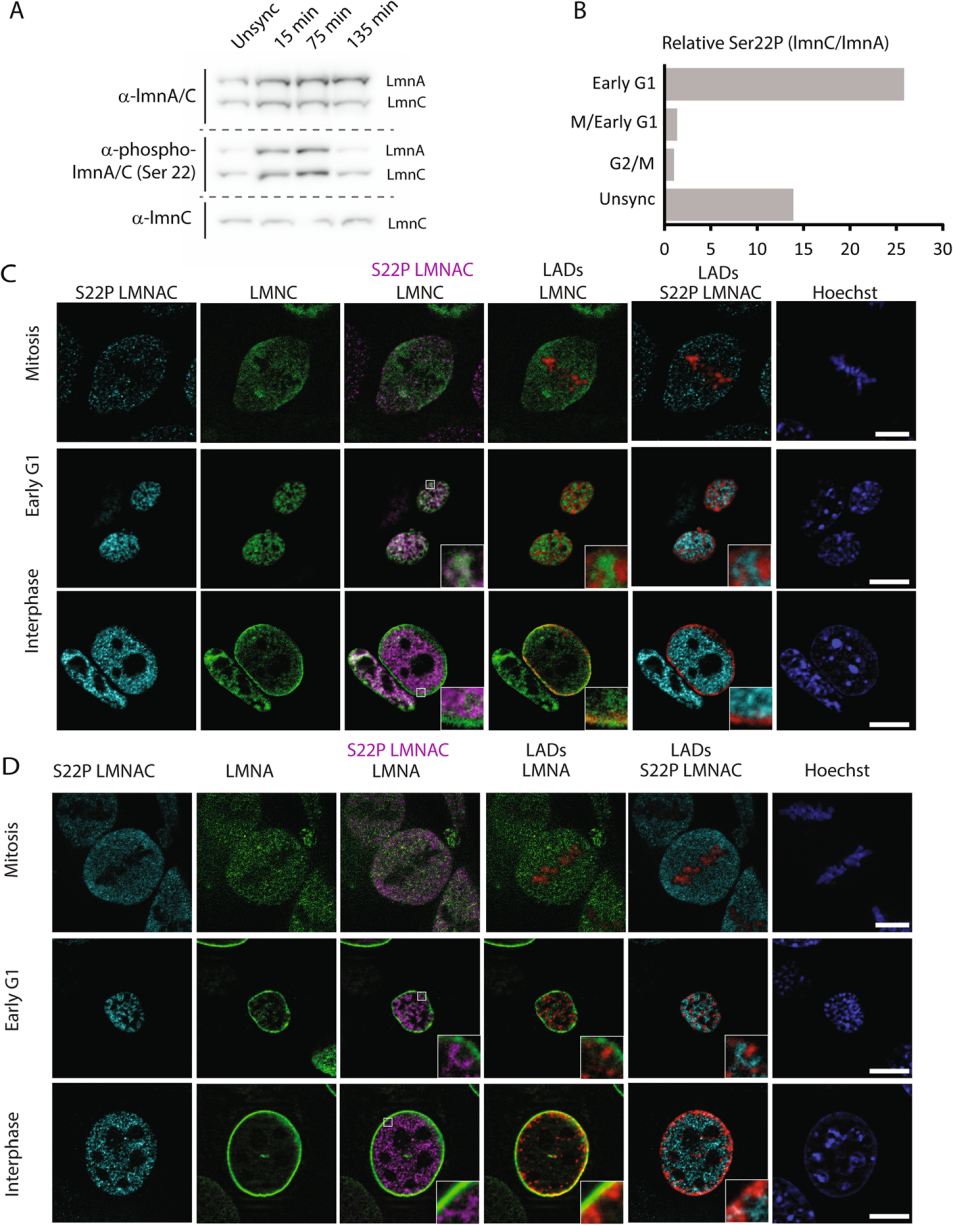
Both lamin A and C are phosphorylated on serine 22 during mitosis and this modification is found on nucleoplasmic lamins, particularly lamin C, in interphase. **A** Representative western blot of whole cell lysates from MEFs that have been unsynchronized or synchronized and released for 15min, 75min, or 135min, stained for lamin A/C, lamin C, or phospho-serine 22 lamin A/C. **B** Quantification of western blot. The phosphorylation levels (detected by anti-phospho serine 22 lamin A/C) of lamin A and lamin C were first normalized to their endogenous levels of the isoforms (lamin A/C staining) to account for differential expression of lamin A and C relative to each other. The ratio of the normalized levels of phosphorylated isotypes is then plotted to monitor relative changes in phosphorylation, with a ratio of “1” correlating with lamin A and C having equal amounts of phosphorylation. **C** Representative images of MEFs expressing the LAD tracer system (red) and EGFP-lmnC (green) stained with anti-phospho-serine Lamin A/C (cyan or magenta) and counterstained with Hoechst 33342 (blue) during mitosis, early G1 and interphase. Phospho-serine 22 lamin A/C was pseudo-colored magenta to enhance contrast with the lamin C overlay. Scale bar indicated by white line is 10μm. **D** Representative images of MEFs expressing the LAD tracer system (red) stained with anti-phospho-serine lamin A/C (cyan or magenta) and anti-lamin A (green), and counterstained with Hoechst 33342 (blue) during mitosis, early G1 and interphase. Phospho-serine 22 lamin A/C was pseudo-colored in magenta to enhance contrast with the lamin A overlay. Scale bar indicated by white line is 10μm

To explore the possibility that lamin C remains preferentially phosphorylated upon exit from mitosis relative to lamin A, we arrested cells at G2/M by first subjecting them to a single thymidine block, followed by a release and a subsequent treatment with the Cdk1 inhibitor RO3306 [9, 60], and a final release into mitosis. Cells were harvested at various time points after release from G2/M and the relative amounts of Ser22 phosphorylation between lamins A and C were analyzed. As expected, both lamin isotypes were equally phosphorylated at the start of mitosis (Fig. 5A, B). In contrast, the levels of Ser22 phosphorylation became more lamin C biased at the later time point as cells begin to exit mitosis, suggesting the persistence of the modification on lamin C (Fig. 5A, B). Importantly, and as expected of Ser22 phosphorylation on A-type lamins, immunofluorescent staining of cells in early G1 with an antibody against Ser22P revealed a nuclear “veil” staining, colocalizing with a substantial pool of lamin C that remains nucleoplasmic (Fig. 5C). Taken together, these data suggest that the preservation of Ser22P on lamin C as cells exit mitosis confers lamin C the ability to remain nucleoplasmic.

As mentioned above, a recent study found that ser22 phosphorylated A-type lamins interact with active promoters and enhancers [54]. As we have shown, lamin C is the isotype that is predominantly phosphorylated through most of interphase. Lamin C has also been shown to preferentially interact with nuclear pore complex proteins, suggesting that at least a subset of these enhancer/promoter interactions would be interacting with, and potentially at, NPCs [34]. To test this hypothesis, we intersected genome-wide data from two studies in human fibroblast cells, one measuring NUP interactions with chromatin and the other euchromatin bound lamin A/C. A subset of the lamin A/C euchromatic interactions were also aligned with NUP interactions, above what would be expected by chance (Additional file 1:Fig. S17).

### Lamin C is required during G1 phase for LAD integrity and LAD recruitment to the NE

**Fig. 6:**
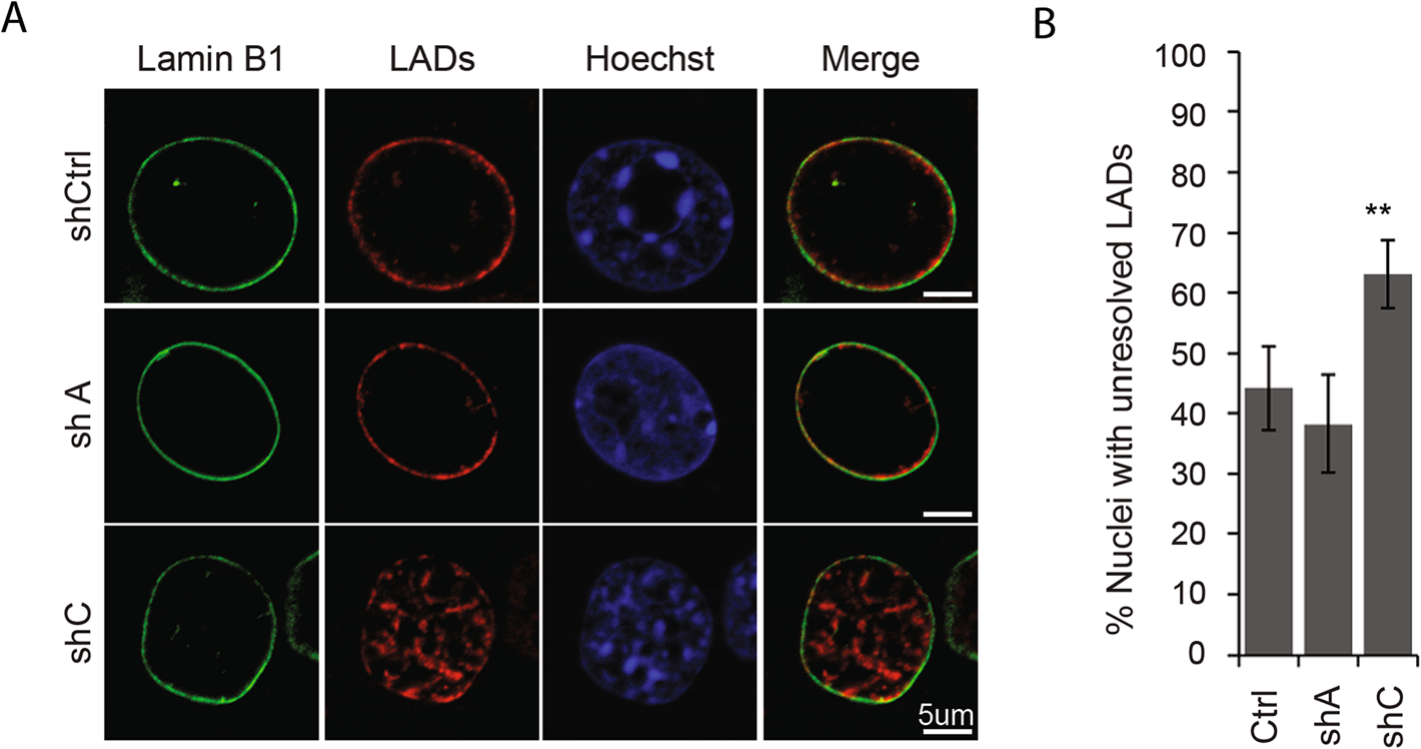
Lamin C ensures the integrity of LAD aggregates and relocalization during early G1. **A** Cells shown 4 h after release from G2/M border. Anti-lamin B1 (green), LAD tracer (red), and Hoechst 33342 (blue). **B** Blinded quantification of nuclei with unresolved LADs (nuclei with LADs not at the periphery) 4 h after release from the G2/M border (*n* ≥ 120). Error bars indicate SD. Statistically significant differences in percent of nuclei with unresolved LADs when comparing shA or shC relative to shCtrl (*p* ≤ 0.001) is indicated by asterisks

To test if lamin C might play a role in restoring LAD organization after mitosis, we used shRNAs to specifically deplete lamin A or lamin C for 4 days to allow lamin turnover in MEF cells harboring the LAD-tracer system. These shRNA-treated cells were then subjected to a single thymidine block (24 h), followed by release into enriched media for several hours and subsequently treated with the Cdk1 inhibitor RO3306 to arrest them at the G2/M transition [9, 60]. After overnight block, cells were released into complete medium (without shield ligand). Cells rapidly entered mitosis and were examined at subsequent time points, up to 4 h later. Because LAD targeting to the NE after mitosis is normally gradual, over several hours, we chose to assay cells 4 h after release from the G2/M block, when a majority of control cells would have exited mitosis and “resolved” interphase LAD organization (Fig. 6A). Nuclei were independently scored by two observers as either “resolved” (all LAD-tracers near lamin B1) or “unresolved” (if any LAD-tracer signal was not adjacent to lamin B1); nuclei were counted over groups of 5 frames and then averaged across groups of 5 (*n*=4 groups, >150 nuclei). Nuclei lacking LAD-tracer signal were not scored. For the shCtrl-treated cells, 44% of nuclei had unresolved LADs (Fig. 6B). We note that this background of “unresolved” LADs is mainly due to timing and shortcomings inherent in systems requiring co-expression of two independent components. We estimated that about 30% of LAD-tracer expressing nuclei simply underexpressed Dam-lamin B1 relative to LAD-tracer, leading to accumulation of diffuse mCherry in the nucleoplasm even in Mid-G1 and, while qualitatively different, these were included in the “unresolved” numbers. Cells depleted of lamin A showed a similar background, with 38% unresolved nuclei (shA; Fig. 6), again suggesting lamin A has no active role in post-mitotic LAD assembly at the NE. By contrast, in the lamin C knockdown population, 62% of nuclei had unresolved LADs, a significant increase of nearly 100% over controls (Fig. 6; *p*<0.001). Interestingly, many disrupted lamin C-depleted nuclei revealed an additional phenotype: LAD aggregates appeared to have decondensed slightly, sometimes forming string-like networks in the nucleoplasm (Fig. 6A), quite distinct from the compact NE-associated LADs in controls (Additional file 1:Fig. S18). This conformation of the heterochromatic LADs was even visible via Hoechst stain (Fig. 6A). Such string-like networks were absent from control and shA treated cells and differed from LAD organization seen in untreated or lamin A-depleted cells immediately after mitosis, where LADs not at the NE remained in clear condensed and separated domains. It is important to note that at 4 h post release, the percentages of cells in M, early G1, and mid-G1/S/G2 were quite similar for all treatments. However, 2 h after release, while the percentages of cells in mitosis and early G1 were the same in control (shLacZ) and shC-treated cells, we note that shA- and shAC-treated cells displayed an apparent lag in early G1 at 2 h (Additional file 1:Fig. S1C). We indicate that this is an apparent lag since we quantify “early G1” on morphometric measures (nuclear size, shape, and/or obvious match to a sister nucleus) and both nuclear shape and size could be influenced by loss of lamin A, since lamin A has been implicated in the mechanoregulation of nuclear morphology. The immediate post-mitosis stage, where the nuclei are still rounded up and the cytoskeleton has not yet exerted its influence on nuclear shape may be a particularly relevant point in the cell cycle for lamin A. This finding makes it even more striking that shA-treated cells do not display perturbed lamin organization. Together, these findings collectively demonstrate that lamin C is required for LAD integrity and dynamic LAD recruitment and association with the NE and nuclear lamina after cell division.

## Discussion

Our findings provide novel insights into 3D genome organization by showing that LAD integrity and post-mitotic association with the NE depend on lamin C, and not on lamin A. To examine the effects of removing specific A-type lamin spliceoforms on LADs and overall genome organization, we employed three technical approaches: (1) Chromosome conformation paints (CCP), (2) Tagged Chromosomal Insertion System (TCIS), and (3) the LAD-tracer system. These three methods allow us to examine genome architecture at different levels of spatial and time resolution in both normal and lamin depleted cells. Our CCP differentially label LADs and non-LADs across an entire chromosome, enabling us to visualize the organization of LADs and non-LADs, relative to each other and the nuclear lamina, in the context of the entire chromosome in situ. The TCIS system, on the other hand, allows us to observe changes to the peripheral localization of a single lamina associated genomic locus (LAS) and enables more robust quantification of perturbations to lamina association through a binary measure of lamina localization. Finally, the LAD-tracer system, which tags all LADs within the nucleus, allows us to measure the dynamics of LAD organization across the genome and relative to the nuclear lamina.

While we and others have previously implicated A-type lamins in regulating LAD organization, we speculated that lamin A and lamin C might have different roles in LAD organization [6]. Both lamin A and lamin C are encoded by the *LMNA* gene with their being crosstalk in their expression levels in a given cell type [49]. Several lines of evidence, from super-resolution microscopy to ectopic expression studies, indicated that lamin C and lamin A form unique networks that are nonetheless inter-dependent on some level [34–36]. Lamin C depleted mice (from birth) show little overt phenotype, with the exception of perturbed nuclear shapes, while mice expressing only lamin C (depleted of A) have longer lifespans [42–44]. Taken together, these data suggest that lamin A and C do indeed have differential roles in the nucleus. One such difference is in how lamin A and C form networks at the lamina, both in timing of association with the NE and in protein:protein interactions. For instance, lamin C preferentially interacts with nuclear pore complexes and altered A/C ratios change the mechanical properties of the nucleus [34, 61]. In this study, we identified a critical role for lamin C in genome organization. In particular, lamin C is critical for both organizing LADs to the nuclear lamina and for LAD sub-chromosomal domain integrity, since acute depletion of this isotype caused derangement, dispersion, and inter-mixing of LAD/non-LAD (A/B compartment) chromatin (Figs. 1 and 2).

Interphase genome organization, including LAD and lamin organization, is ablated during mitosis and re-established after mitosis [8, 9, 16, 25, 26, 29–31]. Previous studies have suggested that A-type lamins organize to the reforming nuclear envelope with different kinetics, although there is some discrepancy on how lamin A and C might differ in their timing of association [49–51, 61]. We find, in agreement with previous studies, that lamin B1 incorporates into the reforming NE at anaphase, preceding both lamin A and C incorporation. Our results, using both fluorescent proteins as well as immunofluorescence, indicate that lamin A precedes lamin C association with the NE in MEFs, with the majority of lamin C remaining nucleoplasmic well into early G1 (Figs. 3, S10, S11 and S12). This is intriguing given that lamin C appears to be critical for LAD organization (Figs. 1 and 2) and we had previously shown that LADs are also nucleoplasmic during this stage of the cell cycle [9].

To further define the spatial and temporal relationship between LAD organization and lamin C organization during the critical transition from mitosis into G1, we used the LAD-tracer system to demarcate LADs in eYFP-lamin C expressing MEFs. Strikingly, lamin C was excluded from heterochromatic LADs at mitotic exit well into early G1 (3 h), suggesting that lamin C predominantly interacts with euchromatic regions of the genome or is excluded from heterochromatic regions in early G1 (Figs. 4, S13, S15, and S16, movie 3 and movie 4). This result struck us as highly intriguing given a recent study showing phosphorylated A-type lamins interact with enhancers and promoters of active genes in interphase [54]. These euchromatin bound lamins were found to be phosphorylated on Serine 22 (a known mitotic PTM) and suggested to be enriched for lamin C. In agreement with these studies, our data strongly support a role for lamin C in the nucleoplasm and in euchromatic regions and we show that lamin A and C are differentially phosphorylated in early G1 (Fig. 5). Furthermore, we posit that interphase euchromatic interactions may be set up during mitosis, as we report, and persist into interphase. (Fig. 5). Our findings reveal that while phosphorylation on Serine 22 is largely lost on lamin A as cells re-enter interphase, the modification is more highly preserved on a pool of lamin C, potentially favoring its nucleoplasmic and euchromatic localization that persists into G1 (Fig. 5). On the other hand, we also find that lamin C is required for normal LAD organization in early G1, suggesting an additional role for this protein. It is intriguing that the timing of LAD organization to the lamina coincides with lamin C recruitment to the nuclear envelope (and its de-phosphorylation) [54]. We also predict that the Serine 22 phosphorylation blocks or alters polymerization of A-type lamins, and enables a control in timing and level of incorporation into the NE [36, 50, 54]. Finally, as determined using a proteomics approach, lamin C preferentially interacts with components of the NPC, a complex associated with euchromatin and depleted in heterochromatin and is enriched on sites where NUPs interact with chromatin [34]. Our finding that lamin C integration into the NE is delayed relative to A also helps explain how independent networks, as detected by super-resolution microscopy, could be achieved. Previous studies found that lamin B1 is recruited to the reforming NE prior to the A-type lamins, thus separate B-networks from A-type lamins made sense. It has been harder to determine why A- and C-networks would remain as structurally separate meshworks, but the delay noted in this study perhaps explains this observation. Thus, as important as lamin C is for organization of LADs, the post-mitotic organization of these heterochromatic domains to the lamina via guided transit towards the NE through direct interactions with lamin C, is unlikely.

What role might lamin C be playing during the transition from mitosis into G1? If lamin C is not directly interacting with LADs, how can it be such an important regulator of LAD organization? The idea that a factor interacting with euchromatin could be important for heterochromatin organization is not far-fetched. Recent work in *Caenorhabditis elegans* has uncovered a heterochromatin independent mechanism for peripheral anchoring. In *C. elegans*, CEC-4 is required to tether heterochromatin to the NE in developing animals, but a second mechanism is evident in differentiated tissues that is dependent on MRG-1, a binder and regulator of euchromatin. In the absence of MRG-1, active modifications invade heterochromatin domains and cause their release from the NE. Thus, a euchromatic factor directly influences and directs peripheral heterochromatin positioning. Lamin C appears to have a dual role: one at the lamina and one in the nucleoplasm. We find that, in the absence of lamin C (but not lamin A), LAD aggregates are delayed or prevented in their association with the NE. Importantly, the LADs appear to form string-like networks of interconnected aggregates (Figs. 6, S18), strongly suggesting that there is a problem in spatially segregating the forming LAD/non-LAD (A/B) chromatin compartments. This is supported by our CCP studies (Fig. 1), in which we observe gross disruption of spatial organization of LAD:LAD interactions, loss of clear A/B intra-chromosomal domain organization, and loss of LAD association with the NE. Our findings therefore suggest that lamin C is dynamically spatially regulated during exit from mitosis to promote novel associations needed for LAD control, and/or to block aberrant interactions and premature reassembly at the NE. We propose that lamin C surrounds LAD agglomerations in the nucleoplasm and prevents their aberrant interactions. In this scenario, nucleoplasmic lamin C serves as a kind of buffer between LADs on different chromosomes, preventing them from sticking to each other and forming a stringy mess or even a large clump in the center, as is seen in some cells lacking lamin A/C and LBR over long periods of time [18].

**Fig. 7:**
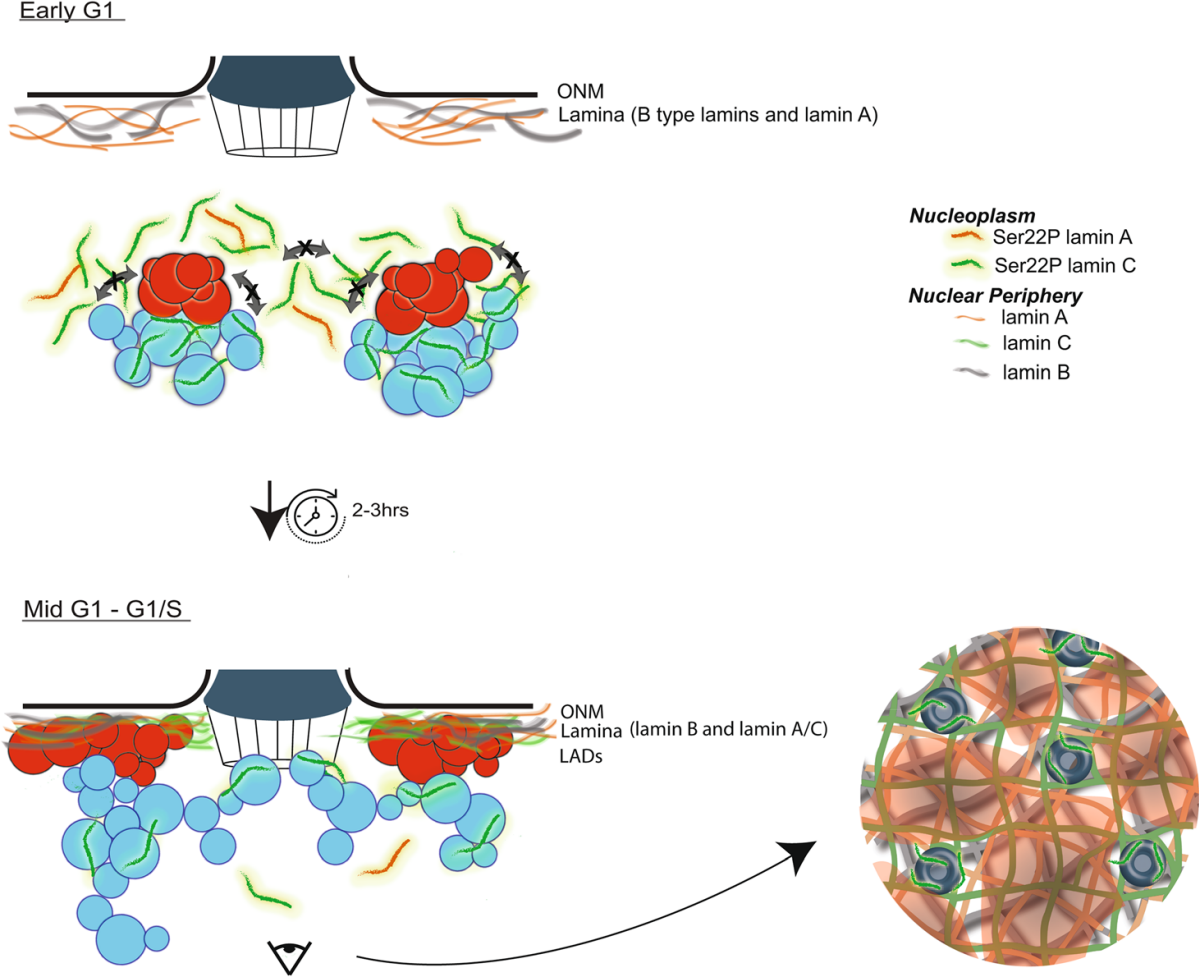
Model of lamin C role in genome organization. Top panel: Post translational modifications (e.g., phospho-serine 22) of lamin C allows its nucleoplasmic localization during mitotic exit and into early G1. During this phase, lamin C is spatially excluded from LADs potentially via protein-protein interactions (for example with Lap2ɑ) on euchromatin and/or phase separation phenomena. We propose that this nucleoplasmic pool physically hinders aberrant inter-chromosomal LAD interactions and reinforces intra-chromosomal A/B compartmentalization, both of which are affected in the absence of lamin C. Bottom panel, left: in mid to late G1, the sustained interchromosomal compartmentalization of LADs is maintained as they, and a subset of lamin C, arrive at the nuclear periphery, as depicted. A subset of phospho-lamin C interacts with euchromatin at NPC. At the lamina, the lamin C that is incorporated into the lamina meshwork is unphosphorylated and we speculate that this de-phosphorylation is required for its incorporation into a NE lamin network. Bottom right panel: inside-out view of LAD/lamina meshwork organization. LADs are shown as transparent red areas and non-LADs are not shown. LADs show robust proximity to all three lamin isotypes, while the lamins A, B, and C form distinct networks. Lamin C (green) is shown as interacting preferentially with nuclear pores (compared to lamins A and B)

We postulate that the temporal separation of incorporation of lamin isotypes at the nuclear periphery post mitosis allows for the formation of separate, but interdependent, lamina meshworks, as has been reported [34]. Our data suggests that lamin B NE meshworks are formed first, followed by lamin A and, lastly, lamin C, much of which remains nucleoplasmic throughout interphase. This late recruitment of lamin C to the NE post-mitosis is strikingly coincident with LAD accumulation to this region, suggesting a coordinated regulation which is supported by LAD disruption in the absence of lamin C. We further speculate that, prior to its accumulation at the nascent NE, lamin C surrounds but is excluded from and might “instruct” genome reorganization by promoting robust LAD-LAD self-association within each chromosome and preventing LAD aggregation between chromosomes; a potential danger since euchromatin and heterochromatin are each capable of self-aggregating via liquid-liquid phase partitioning [23]. Without a mechanism to prevent wide-spread self-aggregation, heterochromatin might globally cluster, leading to chromosome entanglement and difficulty accessing appropriate genes. In support of this, regulated wholesale aggregation of heterochromatin in the center of the nucleus is reported in cell types that lack A-type lamins and LBR, such as rod photoreceptor cells [62]. We therefore propose a gross overall model (Fig. 7) for the role of lamin C in the reorganization of the genome post-mitosis. We propose that lamin C might directly associate with euchromatin on promoters, enhancers, and borders of compartments and LADs [9, 14]. Integrating our data with a recent study, we propose that lamin C with phosphorylated Ser22, a mitotic modification, will be retained in the nucleoplasm and interact with euchromatin, potentially through its interactions with LAP2ɑ or NPC components [34, 54, 59]. In this model, a heterochromatin (LAD) core would be surrounded by lamin C monomers or short polymers which would serve as a “buffer” between chromosomes. These interactions would also reinforce the separation between A- and B-compartment chromatin in each chromosome and prevent aberrant “sticky” heterochromatin interactions between chromosomes. Lastly, this dual purposed segregation mechanism persists as both LADs and lamin C accumulate at the nuclear periphery, with the latter potentially dependent on or enriched in interactions with NPCs and their associated underlying euchromatin [63, 64].

## Conclusions

In summary, we discovered that lamin C is uniquely required to efficiently target LADs to the NE after cell division and to maintain the integrity of A/B compartments and overall gross 3D genome organization. We propose that lamin C promotes intra-chromosomal LAD aggregation and prevents aberrant trans-chromosomal heterochromatin interactions, and that enrichment of S22P specifically on lamin C may contribute to this role. Our results bring up several additional questions, particularly the role these proteins have in organizing and regulating the genome during development and how our findings might impact how we think and investigate laminopathies that do not directly affect lamin C. To fully understand the molecular pathway by which cells re-establish tissue-specific 3D genome architecture after mitosis, it will be important to establish both the role of post-mitotic PTMs of lamin C and the lamin C protein interactome during exit from mitosis.

## Methods

### Generation and maintenance of primary murine embryonic fibroblast (MEFs)

For primary MEFs, wild-type 8-week-old C57BL/6J mice were bred and embryos were harvested at E13.5. Individual embryos were homogenized using a razor blade, and cells were dissociated in 3 mL 0.05% trypsin for 20 min at 37°C, then 2 mL of 0.25% trypsin was added and incubated again at 37°C for 5 min. Cells were pipetted vigorously to establish single cells, passed through a 70-μm cell strainer, pelleted and then plated in 10-cm dishes and labeled as P0. MEFs were cultured DMEM High Glucose with 10% FBS, penicillin/streptomycin, L-glutamine, and non-essential amino acids. Cells were cultured for no longer than 5 passages before harvesting for experiments. For initial DamID and m6A tracer experiments, longer term-culture C57BL/6 MEFs were purchased from ATCC (American Tissue Culture Collection, CRL-2752) and cultured according to their established protocols, in medium containing DMEM High, 10% FBS, penicillin/streptomycin and L-glutamine.

### Lamin A/C knockdown

shRNA-mediated knockdown was carried out as described previously. Specifically, virus for knockdowns were generated in HEK 293T/17 cells (ATCC CRL-11268) by co-transfecting VSV-G, delta 8.9, and a plko.1 vector driving the expression of control shRNAs: shLuciferase (shLuc; 5′-CGCTGAGTACTTCGAAATGTC-3′) or shLacz (5′-CGCTAAATACTGGCAGGCGTT-3′), shLmnA/C (shAC; Sigma clone NM_001002011.2-901s21c, 5′- GCGGCTTGTGGAGATCGATAA-3′), shLmnA (shA; produced in our lab), shLmnC (shC; produced in our lab, 5′-TCTCCCACCTCCATGCCAAAG-3′) or shLmnB1 (Sigma clone NM_010721.1-956s1c1, 5′-GCGTCAGATTGAGTATGAGTA-3′) with Fugene 6 transfection reagent (Promega E2691). 10 mM sodium butyrate was then added to the transfected cells 3 h post transfection for an overnight incubation at 37°C, 5% CO_2_. The transfection media containing sodium butyrate was removed the following day and the cells were washed with 1X PBS. Opti-MEM was then added back to the cells which were then incubated at 37°C, 5% CO_2_. Viral supernatant was collected every 12 h up to 3 collections and the supernatant of all 3 collections were pooled. Primary MEFs were cultured as described and incubated overnight with different shRNA viruses per condition supplemented with 4 μg/mL polybrene and 10% FBS for 12–14 h. Fresh MEF media was then added to the cells after the virus was removed and selected with 20 μg/ml blasticidin or 2ug/ml puromycin. For DamID profiling, cells were infected with DamID virus 4 days post shRNA transduction and cultured for an additional 48 h.

### DamID infection

DamID was performed as described previously [5, 6, 14, 65, 66]. Cells were transduced with lentiviruses harboring the Dam constructs. Lentiviral vectors pLGW-Dam and pLGW Dam-LmnB1 were co-transfected with VSV-G and delta 8.9 into HEK 293T/17 packaging cells using the Fugene 6 transfection reagent in DMEM High glucose complete media (DMEM High glucose supplemented with 10% FBS, Penicillin/Streptomycin, L-glutamine). 10 mM sodium butyrate was added to the transfected cells 3 h post-transfection and left overnight. The following day this media was removed and the cells were washed briefly with 1X PBS before Opti-MEM media was added. Supernatants containing viral particles were collected every 12 h between 36 and 72 h after transfection, and these collections were pooled, filtered through 0.45-μM SFCA or PES, and then concentrated by ultracentrifugation. For infection with lentivirus, MEFs were incubated overnight with either Dam-only or Dam-LmnB1 viral supernatant and 4 μg polybrene. Cells were allowed to expand for 2–4 days then pelleted for harvest.

### DamID protocol

MEFs were collected by trypsinization and DNA was isolated using QIAamp DNA Mini kit (Qiagen, 51304), followed by ethanol precipitation and resuspension to 1 μg/ul in 10 mM Tris, pH 8.0. Digestion was performed overnight using 0.5–2.5 μg of this genomic DNA and restriction enzyme DpnI (NEB, R0176) and then heat-killed for 20 min at 80°C. Samples were cooled, then double stranded adapters of annealed oligonucleotides (IDT, HPLC purified) AdRt (5′-CTAATACGACTCACTATAGGGCAGCGTGGTCGCGGCCGAGGA-3′) and AdRb (5′-TCCTCGGCCG-3′) were ligated to the DpnI digested fragments in an overnight reaction at 16°C using T4 DNA ligase (Roche, 799009). After incubation the ligase was heat-inactivated at 65°C for 10 min, samples were cooled and then digested with DpnII for 1 h at 37°C (NEB, R0543). These ligated pools were then amplified using AdR_PCR oligonucleotides as primer (5′-GGTCGCGGCCGAGGATC-3′) (IDT) and Advantage cDNA polymerase mix (Clontech, 639105). Amplicons were electrophoresed in 1% agarose gel to check for amplification and the size distribution of the library and then column purified (Qiagen, 28104). Once purified, material was checked for LAD enrichment via qPCR (Applied Biosystems, 4368577 and StepOne Plus machine) using controls specific to an internal Immunoglobulin heavy chain (*Igh)* LAD region (J558 1, 5′-AGTGCAGGGCTCACAGAAAA-3′, and J558 12, 5′-CAGCTCCATCCCATGGTTAGA-3′) for validation prior to sequencing.

### DamID-seq library preparation

In order to ensure sequencing of all DamID fragments, post-DamID amplified material was randomized by performing an end repair reaction, followed by ligation and sonication. Briefly, 0.5–5 μg of column purified DamID material (from above) was end-repaired using the NEBNext End Repair Module (NEB E6050S) following manufacturer’s recommendations. After purification using the QIAquick PCR Purification Kit (Qiagen, 28104), 1 μg of this material was then ligated in a volume of 20 μL with 1μl of T4 DNA ligase (Roche, 10799009001) at 16°C to generate a randomized library of large fragments. These large fragments were sonicated (in a volume of 200μL, 10mM Tris, pH 8.0) to generate fragments suitable for sequencing using a Bioruptor® UCD-200 at high power, 30 s ON, 30 s OFF for 1 h in a 1.5-mL DNA LoBind microfuge tube (Eppendorf, 022431005). The DNA was then transferred to 1.5-ml TPX tubes (Diagenode, C30010010-1000) and sonicated for 4 rounds of 10 min (high power, 30 s ON and 30 s OFF). The DNA was transferred to new TPX tubes after each round to prevent etching of the TPX plastic. The sonication procedure yielded DNA sizes ranging from 100 to 200 bp. After sonication, the DNA was precipitated by adding 20 μl of 3M sodium acetate pH 5.5, 500 μl ethanol, and supplemented with 3 μl of glycogen (molecular biology grade, 20 mg/ml) and kept at −80°C for at least 2 h. The DNA mix was centrifuged at full speed for 10 min to pellet the sheared DNA with the carrier glycogen. The pellet was washed with 70% ethanol and then centrifuged again at full speed. The DNA pellet was then left to air dry. 20 μl of 10 mM Tris-HCl was used to resuspend the DNA pellet. One microliter was quantified using the Quant-iT PicoGreen dsDNA kit (Invitrogen, P7589). Sequencing library preparation was performed using the NEBNext Ultra DNA library prep kit for Illumina (NEB, E7370S ), following the manufacturer’s instructions. Library quality and size was determined using a Bioanalyzer 2100 with DNA High Sensitivity reagents (Agilent, 5067-4626). Libraries were then quantified using the Kapa quantification Complete kit for Illumina (Kapa Biosystems, KK4824) on an Applied Biosystems 7500 Real Time qPCR system. Samples were normalized and pooled for multiplex sequencing.

### DamID-seq data processing

DamID-seq reads were processed using LADetector (https://github.com/thereddylab/pyLAD), an updated and packaged version of the circular binary segmentation strategy previously described for identifying LADs from either array or sequencing data (https://github.com/thereddylab/LADetector) [5, 6]. LADs separated by less than 25 kb were considered to be part of a single LAD. All other parameters were left at default values. LADs were post-filtered to be greater than 100 kb, complementary genomic regions to LADs were defined as non-LADs. Bed files were generated for visualization using the pyLAD LADetector.

### LAD and non-LAD chromosome-wide probe design and labeling

LADs from murine embryonic fibroblasts were defined through the LADetector algorithm, and complementary regions to chromosomes 11 and 12 were defined as non-LADs [9]. Data provided Geo GSE56990. Centromeres were excluded, and LAD and non-LADs were repeat masked. Probes were selected in silico based on TM and GC content, and those with high homology to off target loci were specifically removed. 150 base pair oligos were chemically synthesized using proprietary Agilent technology and probes were labeled with either Cy3 or Cy5 dyes using the Genomic DNA ULS Labeling Kit (Agilent, 5190-0419). Forty nanograms of LAD and non-LAD probes were combined with hybridization solution (10% dextran sulfate, 50% formamide, 2X SSC) then denatured at 98°C for 5 min and pre-annealed at 37°C.

### Immunofluorescence

Cells were prepared for immunofluorescence by plating on sterilized 15 or 25 mm round coverslips (German borosilicate glass #1.5; Harvard Apparatus) in 12- or 6-well tissue culture dishes. Immunofluorescence was carried out as previously described [67]. The nuclear lamina was visualized using an anti-LMNB antibody (sc-6217, goat IgG; Santa Cruz Biotechnology, Inc.) and Alexa Fluor tagged AffiniPure Donkey Anti-Goat, -Rabbit, or -Mouse (Jackson Immunoresearch) for secondary detection. Other antibodies used for immunofluorescence were Anti-lamin A antibody (ab8980, Mouse IgG3, Abcam) at a concentration of 1/200, Anti-lamin C antibody (ab125679, Rabbit IgG, Abcam) at a concentration of 1/200, and Phospho-lamin A/C (Ser22) (D2B2E) XP® Rabbit (#13448S, Rabbit IgG, Cell Signaling Technology) at a concentration of 1/200. Immunofluorescence samples were mounted in SlowFade Diamond (Invitrogen). All imaging was performed on an inverted fluorescence microscope (AxioVision; Carl Zeiss) fitted with an ApoTome and camera (AxioCam MRm; Carl Zeiss). The objective lens used was a 63× Apochromat oil immersion (Carl Zeiss) with an NA of 1.5 (Immersol 518; Carl Zeiss). All immunofluorescence was performed at room temperature on #1.5 coverslips. AxioVision software (Carl Zeiss) was used for image acquisition. Images were imported into (FIJI ImageJ, National Institutes of Health) for further analyses [68].

### 3D-ImmunoFISH

3D-immunoFISH was performed as described previously [6, 66]. Briefly, primary mouse embryonic fibroblast cells were plated on poly-L-lysine coated slides overnight. Cells on slides were fixed in 4% paraformaldehyde (PFA)/1X PBS for 16 min, then subjected to 3–5-min washes in 1X PBS. After fixation and washing, cells were permeabilized in 0.5% TritonX-100/0.5% saponin for 15–20 min. The cells were washed 3 times 5 min each wash in 1X PBS, then acid treated in 0.1N hydrochloric acid for 12 min at room temperature. After acid treatment, slides were placed directly in 20% glycerol/1X PBS and then incubated at least 1 h at room temperature or overnight at 4°C. After soaking in glycerol, cells were subjected to 4 freeze/thaw cycles by immersing glycerol coated slides in a liquid nitrogen bath. Cells were treated with RNAse (100 μg/ml) for 15 min in 2X SSC at room temperature in a humidified chamber. DNA in cells was denatured by incubating the slides in 70% formamide/2X SSC at 74°C for 3 min, then 50% formamide/2X SSC at 74°C for 1 min. After this denaturation, cells were covered with a coverslip containing chromosome conformation paints in hybridization solution and sealed. After overnight incubation at 37°C, slides were washed three times in 50% formamide/2X SSC at 47°C, three times with 63°C 0.2X SSC, one time with 2X SSC, and then two times with 1X PBS before blocking with 4% BSA in PBS for 30–60 min in a humidified chamber. Slides were then incubated with anti-LmnB1 primary antibody (1:200 dilution; Santa Cruz, SC-6217) in blocking medium overnight at 4°C. Slides were washed three times with 1X PBS/0.05% Triton X-100 and then incubated with secondary antibody in blocking medium Alexa Fluor 488 (1:200 dilution; A32814) for 1 h at room temperature. Post incubation, slides were washed three times with 1X PBS/0.05% Triton X-100, and then DNA counterstained with 1 μg/ml Hoechst. Slides were then washed, mounted with SlowFade Gold (Life Technologies, S36936).

### LAD object segmentation

Images corresponding to LADs were segmented and the volume of each object was obtained using the 3D objects counter tool in FIJI [68, 69]. The images were first thresholded in an unbiased fashion based on the signal intensity from the medial plane and the minimum size filter was then set to 100.

### Live cell imaging

Immortalized C57BL/6 MEF (ATCC CRL-2752) cells were infected to stably express ddDam-laminB1-CDT, eGFP-lamin C, and m6A tracer. ddDam-laminB1-CDT is a destabilized version of the previously described DamID construct that has incorporated the CDT domain from the Fucci system to ensure its expression is restricted to interphase [9, 16, 56, 57]. The m6A-tracer is comprised of a catalytically inactive version of DpnI that retains its ability to bind DNA, in frame with an mCherry red fluorescent protein [16, 56]. For cell cycle experiments, these cells were grown in the presence of shield ligand (AOBIOUS, AOB6677), which stabilizes the ddDam-laminB1-CDT, along with 1mM thymidine (Sigma) block for 24 h to enable synchronization of cells at G1/S. This arrest was followed by release into complete DMEM medium (DMEM high glucose, +10%FBS, 100 U/mL Penicillin and 100 μg/mL Streptomycin) containing 25μM 2′-Deoxycytidine for 4 h. Cells were then blocked at G2/M by incubation by replacing media with complete media containing 10uM R0-3306 (AOBIOUS, AOB2010) for 16–20 h [60]. Cells were released from this block by washing 3 times with warm Fluorobrite DMEM +10% FBS with 100 U/mL Penicillin and 100 μg/mL Streptomycin. One to 4 h after release, cells were imaged live every 1–5 using a 3i spinning disc confocal microscope. Interphase cells were not synchronized and were imaged every 1–5 min.

### TCIS

The two TCIS clones, clone Y and clone 12, harbored a LAS corresponding to a fragment of the *Ikzf1* gene as previously described [6]. C57BL/6 fibroblasts were transfected, using Fugene 6 (Promega), with a linearized TCIS construct described previously [6]. Cells were selected for hygromycin resistance (500 μg/ml), and clones were isolated and expanded. Single integration clones were screened for by qPCR and transfection with EGFP-LacI retroviral vector to visualize the insert site. Clones 12 and Y had single integrations of the TCIS system at a chromosomal position away from the nuclear lamina, as determined by microscopy and either the presence or lack of an overlap in LMNB1 and EGFP-LacI accumulation at the lacO insert site. Site-specific recombination was obtained by cotransfection of TCIS clones with a DNA fragment corresponding to the LAS (Ikzf1) cloned into a switch vector and Cre recombinase. Switched cells were then seeded at low density with 10,000 cells per well of a 6-well tissue culture dish and treated with 1 μM ganciclovir for 24 h. TCIS cells require a short treatment with ganciclovir and to be treated at low confluence. Negative ganciclovir selection occurs when the non-switched thymidine kinase gene cassette expresses thymidine kinase, which in turn phosphorylates ganciclovir. Phosphorylated ganciclovir is toxic to the cells. Once released into the media, it can affect neighboring cells if not maintained at low confluence and if media is removed after 24 h. Cells that have successfully switched cannot phosphorylate ganciclovir and are therefore resistant. Cells resistant to ganciclovir (1 μM) were then expanded for nuclear positioning analysis. Transfections for specific recombination in TCIS clones were performed with the electroporation system (Amaxa Nucleofector 4; Lonza), to ensure essentially 100% transfection efficiency. Ingenio Electroporation Products (MIR 50111; Mirus Bio LLC) were used in combination with the Amaxa nucleofector. All cell lines were maintained in DMEM high with 10% FBS (U.S. Defined Fetal Bovine Serum; Hyclone) in the presence of 500 μg/ml hygromycin (50 mg/ml; Hygromycin B; Corning/CellGro) and 1 mM IPTG when EGFP-LacI was present. To enable binding of EGFP-LacI, IPTG was removed from the cultures, and cells were analyzed after 24–36 h in fresh media.

### Fluorescent tagged lamin overexpression

Lentiviral vectors containing fluorescently tagged lamin A or C were co-transfected with VSV-G and delta 8.9 into HEK 293T/17 packaging cells using the Fugene 6 transfection reagent in DMEM High glucose complete media (DMEM High glucose supplemented with 10% FBS, Penicillin/Streptomycin, L-glutamine). 10 mM sodium butyrate was added to the transfected cells 3 h post-transfection and left overnight. The following day this media was removed and the cells were washed briefly with 1X PBS before Opti-MEM media was added. Supernatants containing viral particles were collected every 12 h between 36 and 72 h after transfection, and these collections were pooled, filtered through 0.45-μM SFCA or PES, and then concentrated by ultracentrifugation. For infection, MEFs were incubated overnight with either mCherry-LmnA or eYFP Lamin C viral supernatant and 4 μg polybrene. Cells were allowed to expand in selection media containing 2μg/mL puromycin or 20μg/mL blasticidin respectively for 2–4 days followed by a second round of transduction with the other fluorescent tagged lamin viral supernatant followed by expansion in selection media containing both 2μg/ml puromycin and 20μg/ml blasticidin.

### GenometriCorr

To determine positional correlation between genome binding sites of S22-phosphorylated lamin A/C and nuclear pores, we input publicly available data into the R package, GenometriCorr [70]. GenometriCorr is an R package used to determine the correlation of different genome wide datasets or biological features. Human fibroblasts Nup153 DamID peaks from GSE 87831and S22P lamin A/C peaks from GSE113354 were used as input to GenometriCorr [54, 71]. To test the positional correlation of each Nup153 peak with neighboring S22P lamin A/C peaks, the S22P lamin A/C peak dataset was set as the reference dataset and the Nup153 peaks dataset was set as the query.

### Cell cycle synchronization

Immortalized C57BL/6 mouse embryonic fibroblast (ATCC C-57Bl/6) cells were plated at a confluency of 15–20% in complete DMEM (High glucose, Penicillin-Streptomycin, 10% FBS) and allowed to rest in a tissue culture incubator (37°C, 5% CO_2_, high humidity) for 2 h before addition of thymidine to induce cell cycle arrest at the G1/S interphase. Approximately 22–24 h later, thymidine was removed, and cells were left to rest in a tissue culture incubator for 3 h with 2′ deoxycytidine to promote release and progression past the G1/S checkpoint. At this point, the CDK inhibitor, RO3306 (AOBIOUS), was added to induce cell cycle arrest at the G2/M checkpoint. To enrich for different points as cells transit mitosis and enter G1, cells were released for different amounts of time. The majority of cells enter mitosis within the first 30 min, with most cells in G1 by 135 min post-release. These times can vary depending on how frequently the cells are being removed from the tissue culture incubator since this affects the temperature of the media.

### Western blots

Immortalized mouse embryonic fibroblast (MEF) cells (ATCC C-57BL/6) were synchronized to enrich for specific cell cycle stages. Protein extracts were made from unsynchronized, blocked, 15-min release, 60-min release, and 120-min release. After protein extraction, samples for loading were prepared with 4x laemmli and 10% Beta-MercaptoEthanol. Thirty micrograms of each sample as determined by BCA quantitation was loaded into each well of the gel (10% Tris-Glycine, Novex NuPage, Invitrogen) for SDS-PAGE. Gels were subsequently transferred to a PVDF membrane using the Transblot SD semi-dry transfer system (Bio-Rad) . Post transfer the membrane was blocked in TBST (Tris-Buffered Saline 0.1% Tween 20) with 5% milk (w/v) for 1 h followed by three washes in TBST. The membrane was then incubated primary antibody diluted in 1% milk in TBST overnight, with nutation or rocking at 4°C. Primary antibodies used for western blots were Anti-lamin A antibody (ab8980, Mouse IgG3, Abcam) at a concentration of 1/200, Anti-lamin C antibody (ab125679, Rabbit IgG, Abcam) at a concentration of 1/500, anti-LMNB antibody (sc-6217, goat IgG; Santa Cruz Biotechnology, Inc.) at a concentration of 1/5,000, Anti-lamin A + lamin C antibody [EP4520-16] (ab133256, Rabbit monoclonal, Abcam) at a concentration of 1/1,000, and Phospho-lamin A/C (Ser22) (D2B2E) XP Rabbit (#13448S, Rabbit IgG, Cell Signaling Technology) at a concentration of 1/1,000. After incubation with primary antibody, the membrane was washed 3 times in TBST, followed by a 1–2-h incubation in HRP-coupled secondary antibody (Donkey anti-Goat (ca#705036147), Donkey anti-Mouse (cat#715036150), and Donkey anti-Rabbit (cat#711035152), from Jackson ImmunoResearch Laboratories, Inc. with a working concentration of 1/10,000) at room temperature diluted in 1% milk in TBST. After washing 3 times in TBST (10 min each), the blot was developed using Clarity Western ECL Substrate (cat#1705060) reagents and the emitted luminescence captured by an Azure Biosystems c600 multi-modal imaging system. For full western blots, see Additional file 2.

## Supplementary Information

**Additional file 1.** Contains all supplemental figures

**Additional file 2.** Contains images of full western blots

**Additional file 3.** Review history

## Data Availability

The data generated for this publication have been deposited in NCBI’s Gene Expression Omnibus and are accessible through GEO Series accession number GSE97095 [72]. Other high throughput datasets discussed are available from GEO under GSE124205 [9], GSE87831 [71], GSE113354 [54], and GSE8854 [14].
