# Supplementary material for: Lamin C is required to establish genome organization after mitosis
Source: Genome Biol. 2021 Nov 15;22:305. doi: 10.1186/s13059-021-02516-7 (PMC8591896; doi:10.1186/s13059-021-02516-7)
Supplement: Supplementary file 1 — Additional file 1. Contains all supplemental figures [file 13059_2021_2516_MOESM1_ESM.pdf]

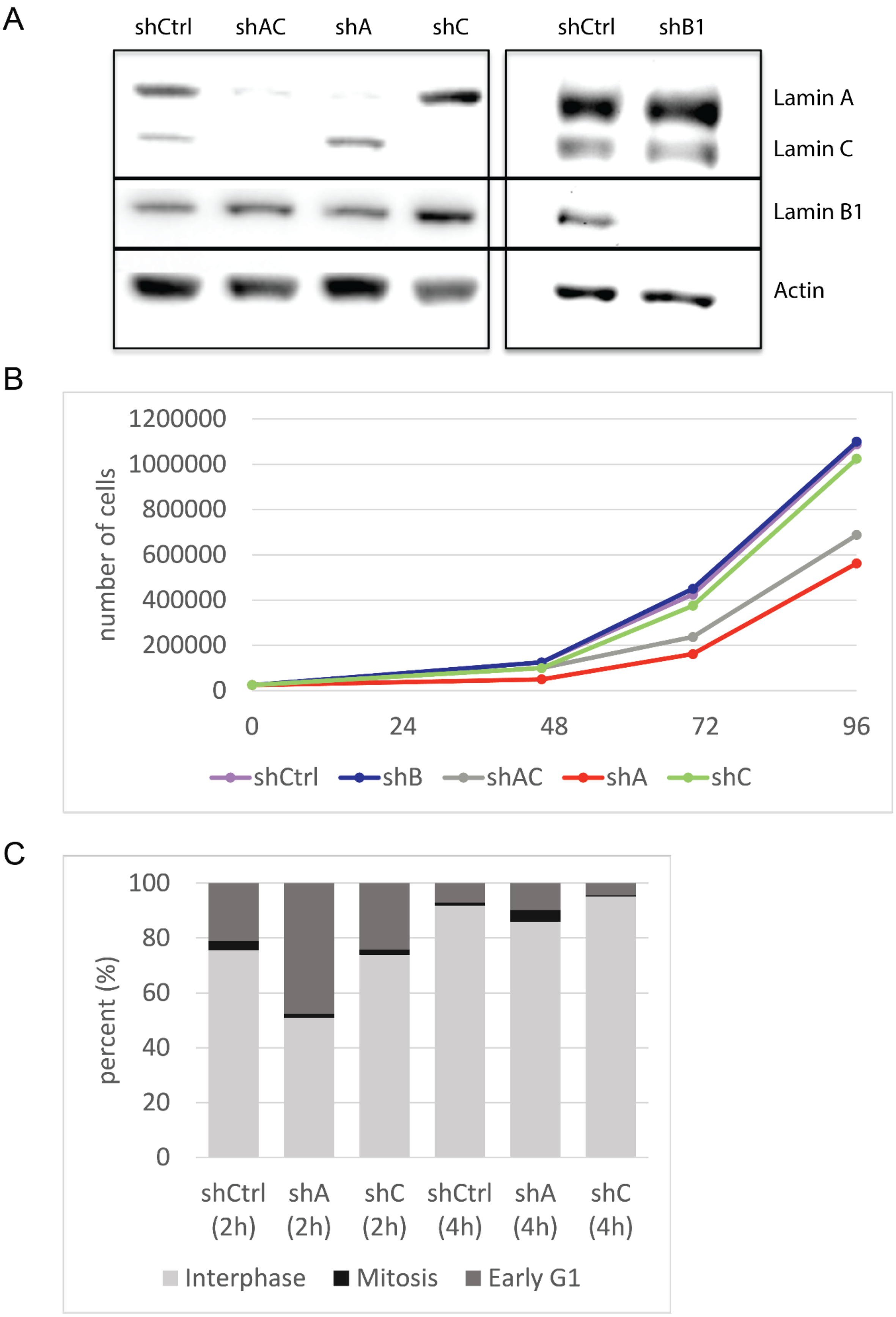
A
shCtrl
shAC
shA
shC
shCtrl
shB1
Lamin A
Lamin C
Lamin B1
Actin
B
number of cells
1200000
1000000
800000
600000
400000
200000
0
0
24
48
72
96
shCtrl
shB
shAC
shA
shC
C
percent (%)
100
80
60
40
20
0
shCtrl (2h)
shA (2h)
shC (2h)
shCtrl (4h)
shA (4h)
shC (4h)
Interphase
Mitosis
Early G1

S1: Assessment of shRNA efficacy and effects on cell cycle progression

(A) Representative western blots showing specificity of the various knockdown constructs. (B) 25,000 cells were plates at 0 hours. Graph represents the number of cells on each plate after a given number of hours for each shRNA treatment indicated (shControl, shB, shAC, shA, shC). (C) Graph indicates the percentage of synchronized cells in either interphase, mitosis or G1 as assessed from nuclear lamina morphology either at 2 hours or 4 hours after release into mitosis for each condition (shControl, shA or shC).

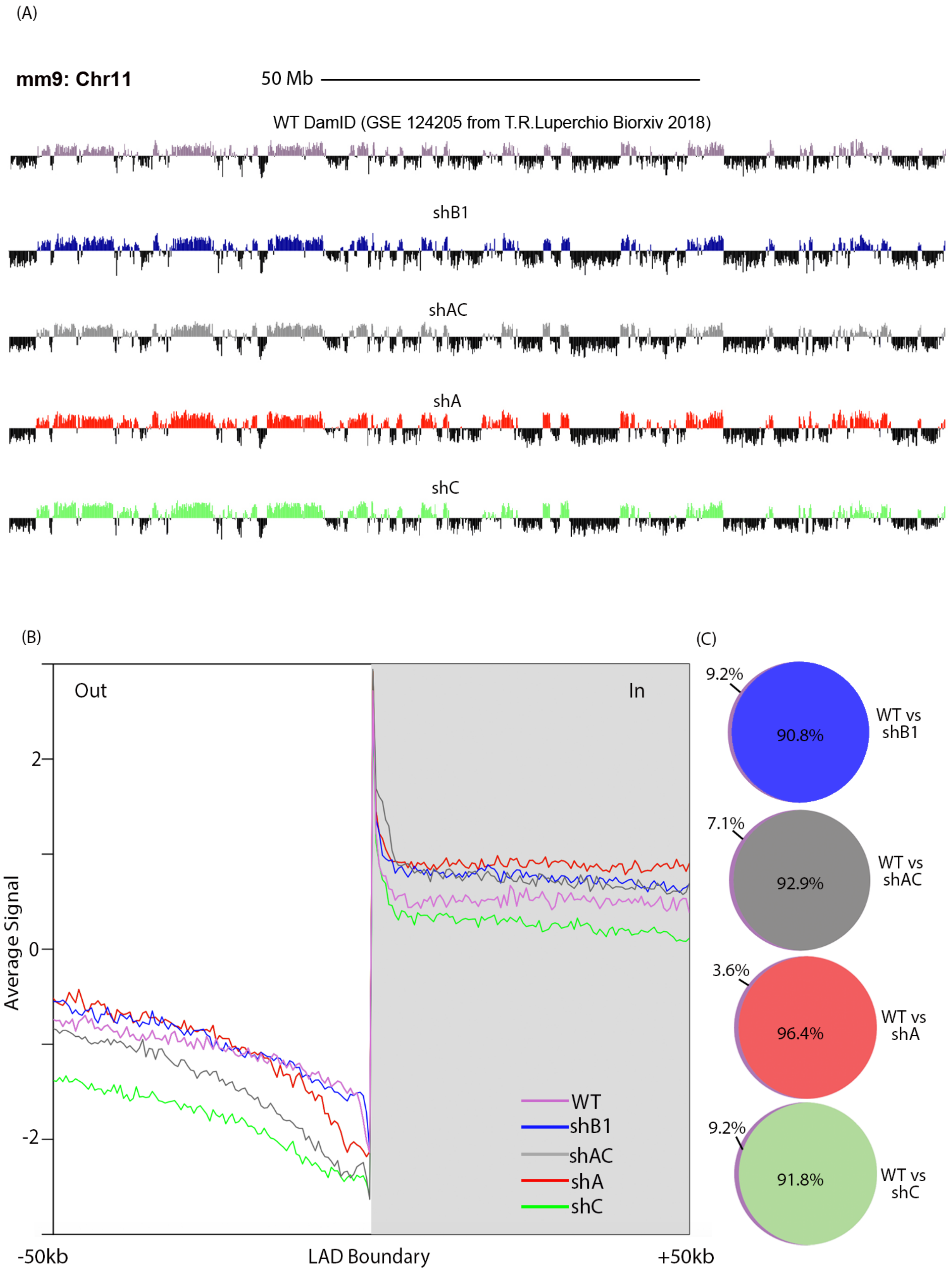
(A)
mm9: Chr11
50 Mb
WT DamID (GSE 124205 from T.R.Luperchio Biorxiv 2018)
shB1
shAC
shA
shC
(B)
Out
In
Average Signal
2
0
-2
-50kb
LAD Boundary
+50kb
WT
shB1
shAC
shA
shC
(C)
9.2%
90.8%
WT vs shB1
7.1%
92.9%
WT vs shAC
3.6%
96.4%
WT vs shA
9.2%
91.8%
WT vs shC

Fig S2: DamID shows no discernible LAD perturbations in the absence of different lamin isotypes

(A) Chromosome wide DamID traces (chr 11) for wildtype (purple), lamin B1 depleted (blue), lamin A depleted (red), lamin A/C depleted (grey) and lamin C depleted (green) samples. Vertical axes are of log2 scale and traces above 0, in darker shades, indicate a higher than expected frequency of peripheral association. (B) Profiles of aligned LAD border regions (left and mirrored right border regions combined) are shown for lamin B1 occupancy. To align LAD borders, genome-wide positions of lamin B1 occupancy were converted to coordinates relative to the nearest border. Gray area and positive coordinates, inside LADs; white area and negative coordinates, outside LADs. (C) Venn Diagram showing the proportion of wild type LAD domains preserved in absence of lamin B or lamin A or C alone or both lamins A and C.

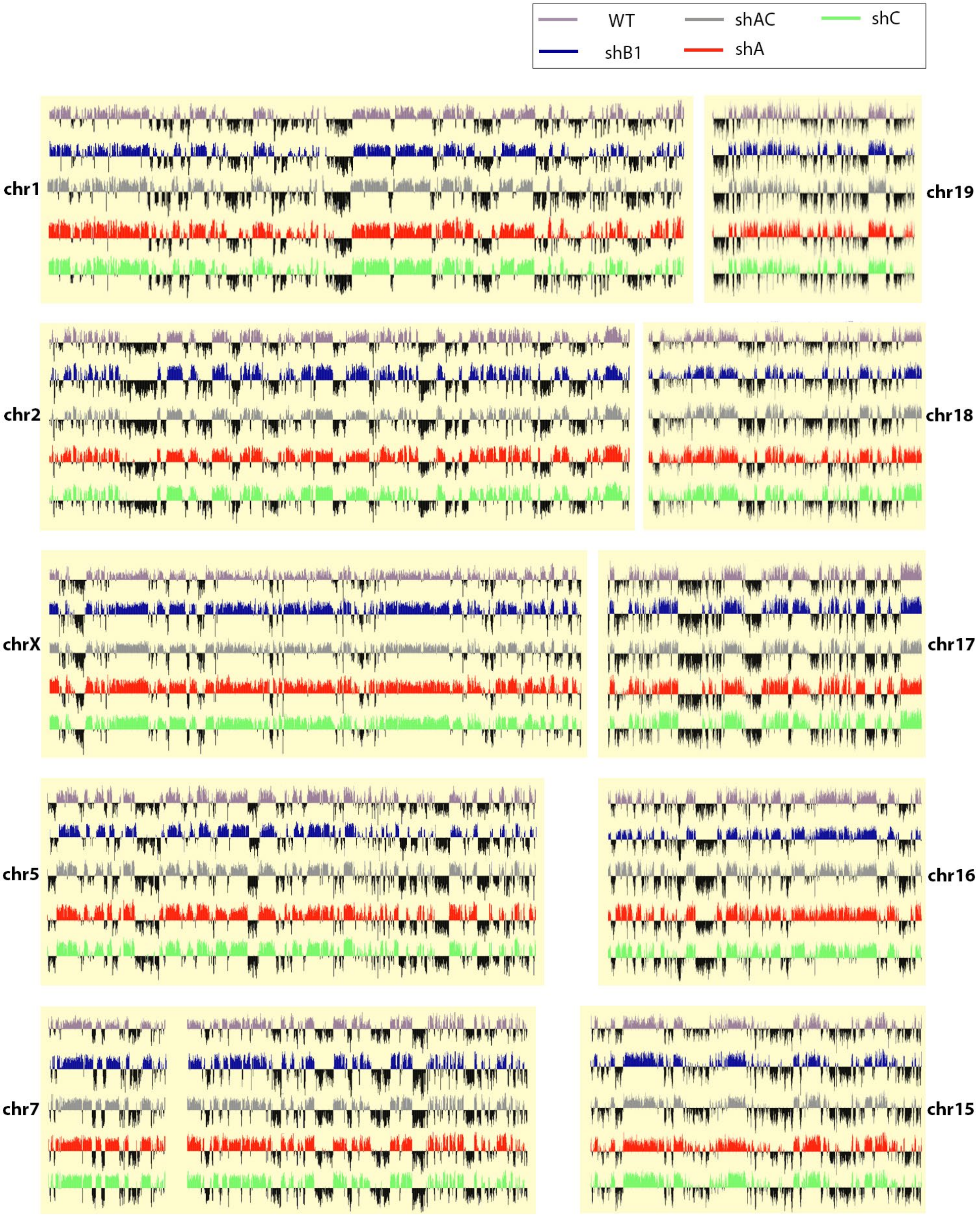
WT
shAC
shC
shB1
shA
chr1
chr19
chr2
chr18
chrX
chr17
chr5
chr16
chr7
chr15

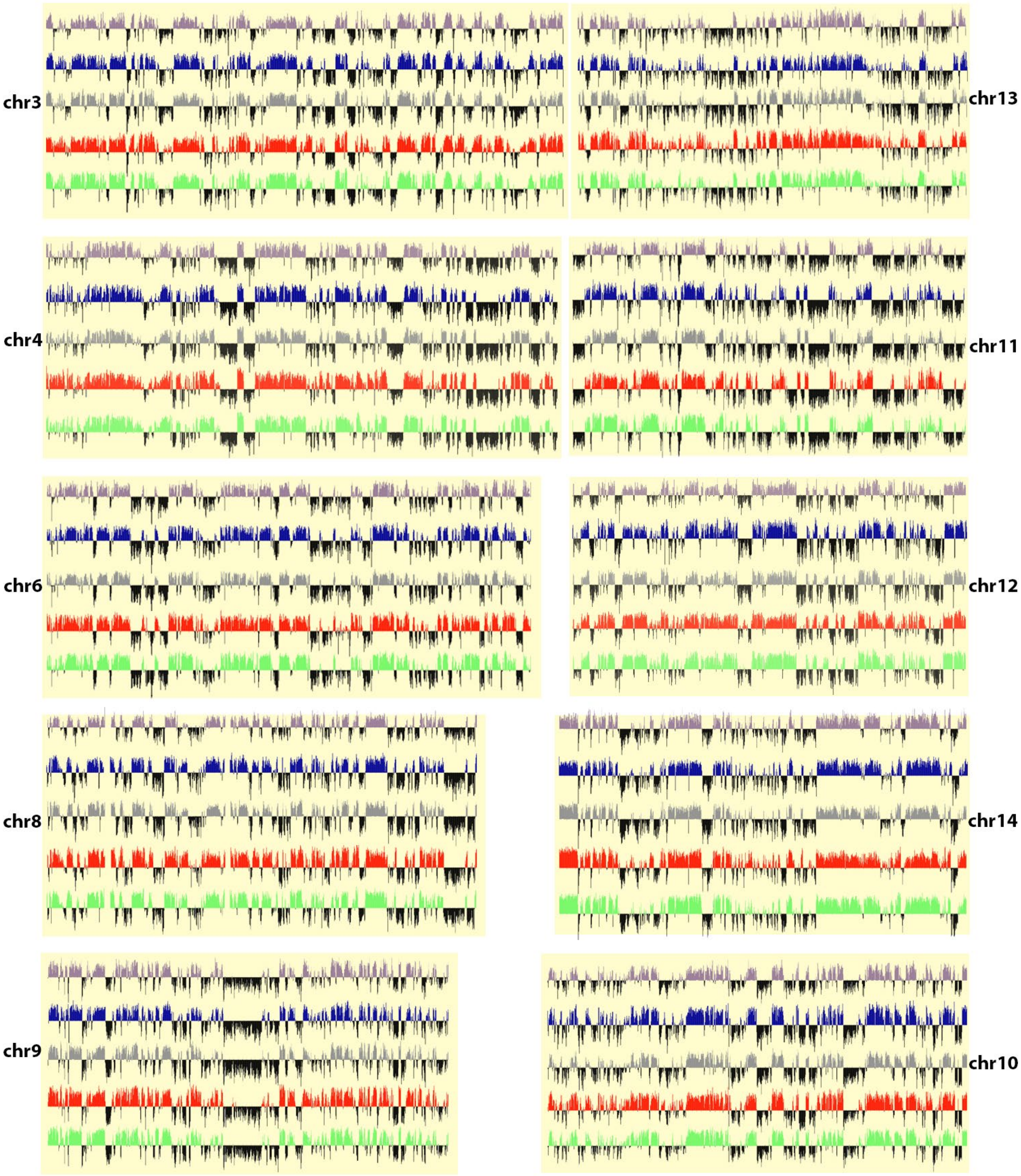

Fig S3: DamID Traces for all chromosomes

Chromosome wide DamID traces for wildtype (purple), lamin B depleted (blue), lamin A depleted (red), lamin A/C depleted (grey) and lamin C depleted (green) samples. Vertical axes are of log2 scale and traces above 0, in darker shades, indicate a higher than expected frequency of peripheral association. Scale bar = 2μm.

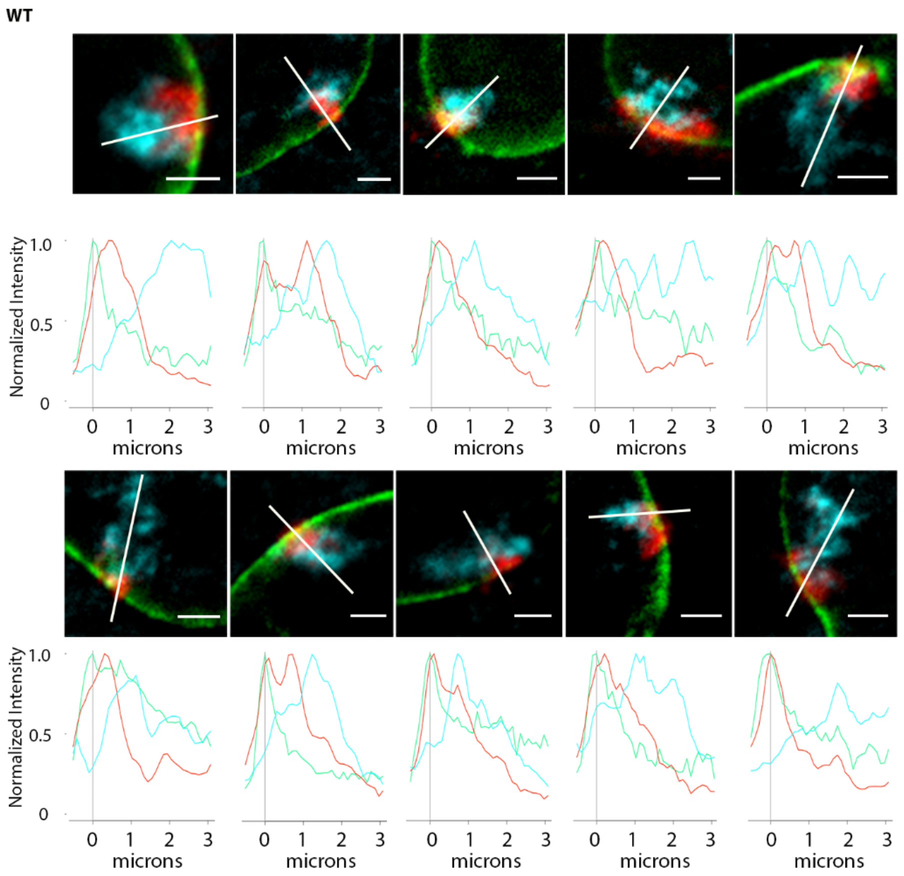

Fig S4: Wild type chromosomes show LAD aggregation and peripheral association
Representative images of chromosome conformation paints to nonLADS (cyan), LADs (red), and lamin B1 (green). Normalized fluorescence intensity histogram plots for chromosome 11 territories in wild type MEFs, plotted from the nuclear lamina (0µm) to 3µm into the nucleus. The line each plot travels through is represented by a white line. Scale bar = 2µm.

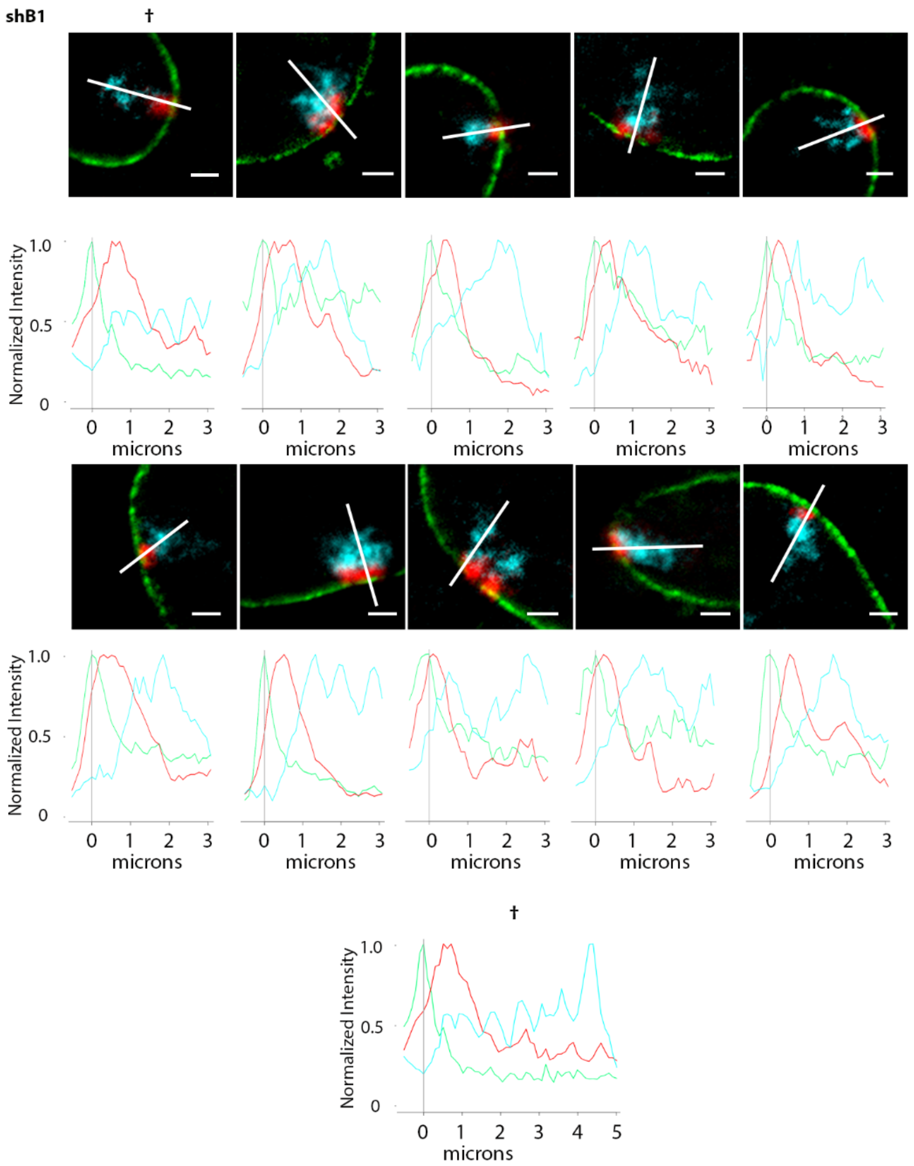

Fig S5: Knockdown of lamin B1 does not perturb LAD aggregation or peripheral association
Representative images of chromosome conformation paints, highlighting nonLADS (cyan) and LADs (red), and lamin A/C (green). Normalized fluorescence intensity histogram plots for chromosome 11 territories in knockdown of lamin B1 using a specific shRNA, plotted from the nuclear lamina (0μm) to 3μm into the nucleus. The line each plot travels through is represented by a white line. Scale bar = 2μm.

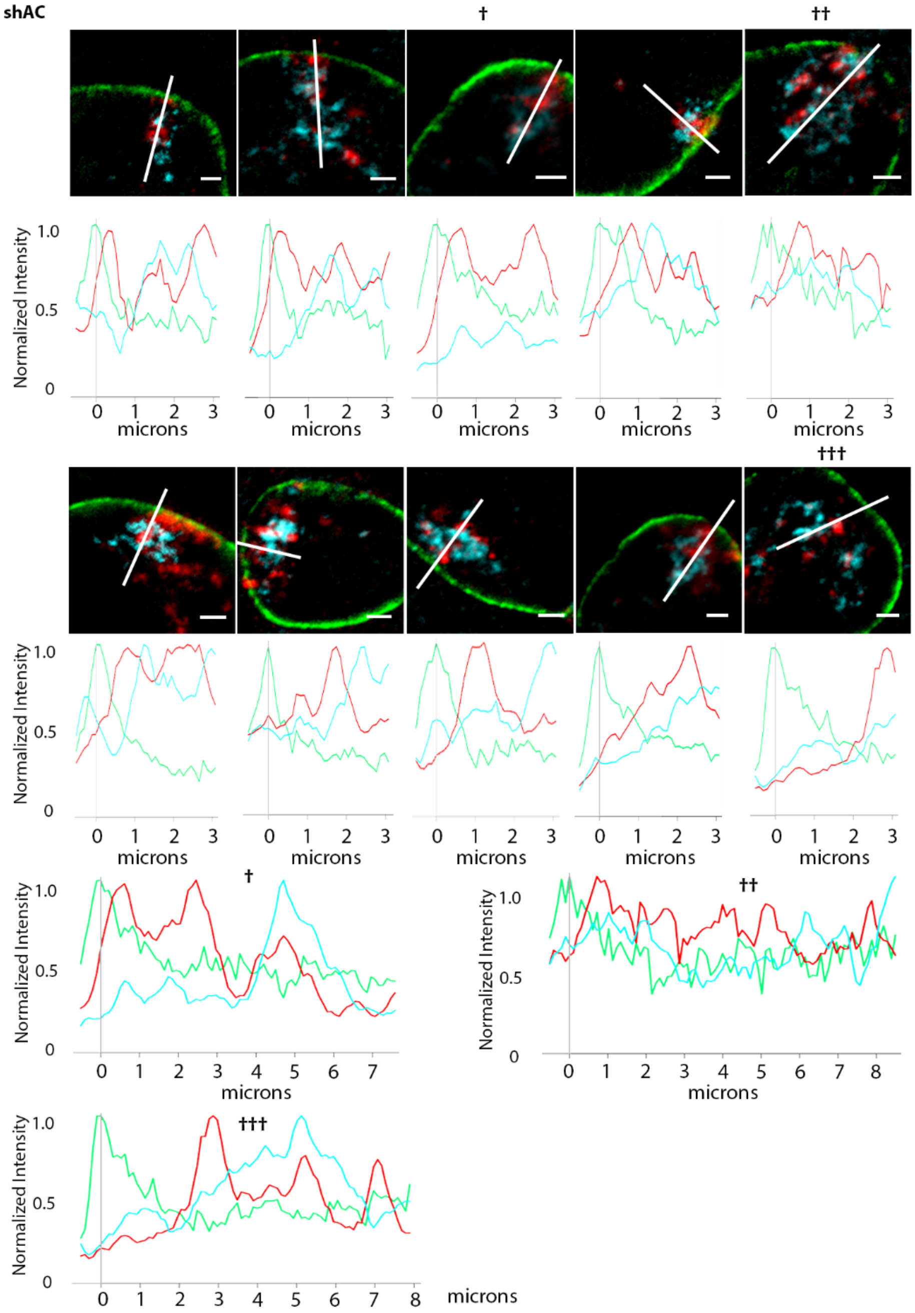

Fig S6: Knockdown of lamin A/C shows disrupted genome organization

Representative images of chromosome conformation paints, highlighting nonLADS (cyan) and LADs (red), and Lamin B1 (green). Normalized fluorescence intensity histogram plots for chromosome 11 territories in knockdown of Lamin A/C using a specific shRNA, plotted from the nuclear lamina (0μm) to 3μm into the nucleus. Territories marked with †(s) were plotted beyond 3μm to display the extent of LAD signals. The line each plot travels through is represented by a white line. Scale bar = 2μm.

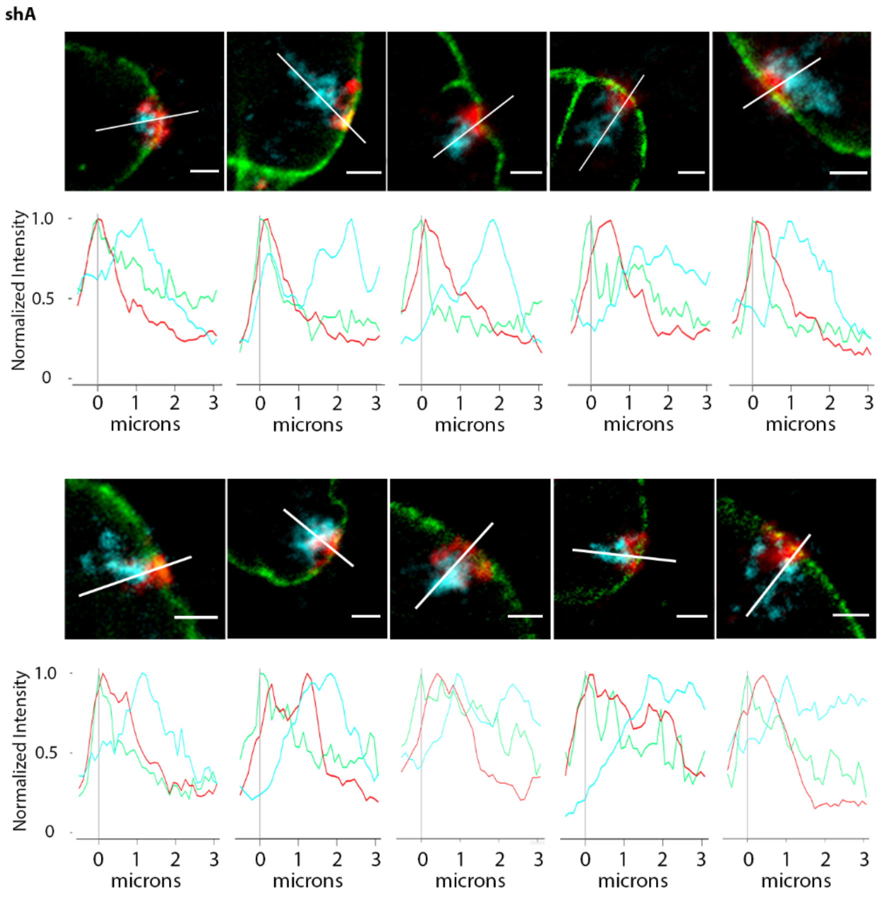

Fig S7: Knockdown of lamin A does not perturb LAD aggregation or peripheral association Representative images of chromosome conformation paints, highlighting nonLADS (cyan) and LADs (red), and lamin B1 (green). Normalized fluorescence intensity histogram plots for chromosome 11 territories in knockdown of lamin A using a specific shRNA, plotted from the nuclear lamina (0μm) to 3μm into the nucleus. The line each plot travels through is represented by a white line. Scale bar = 2μm.

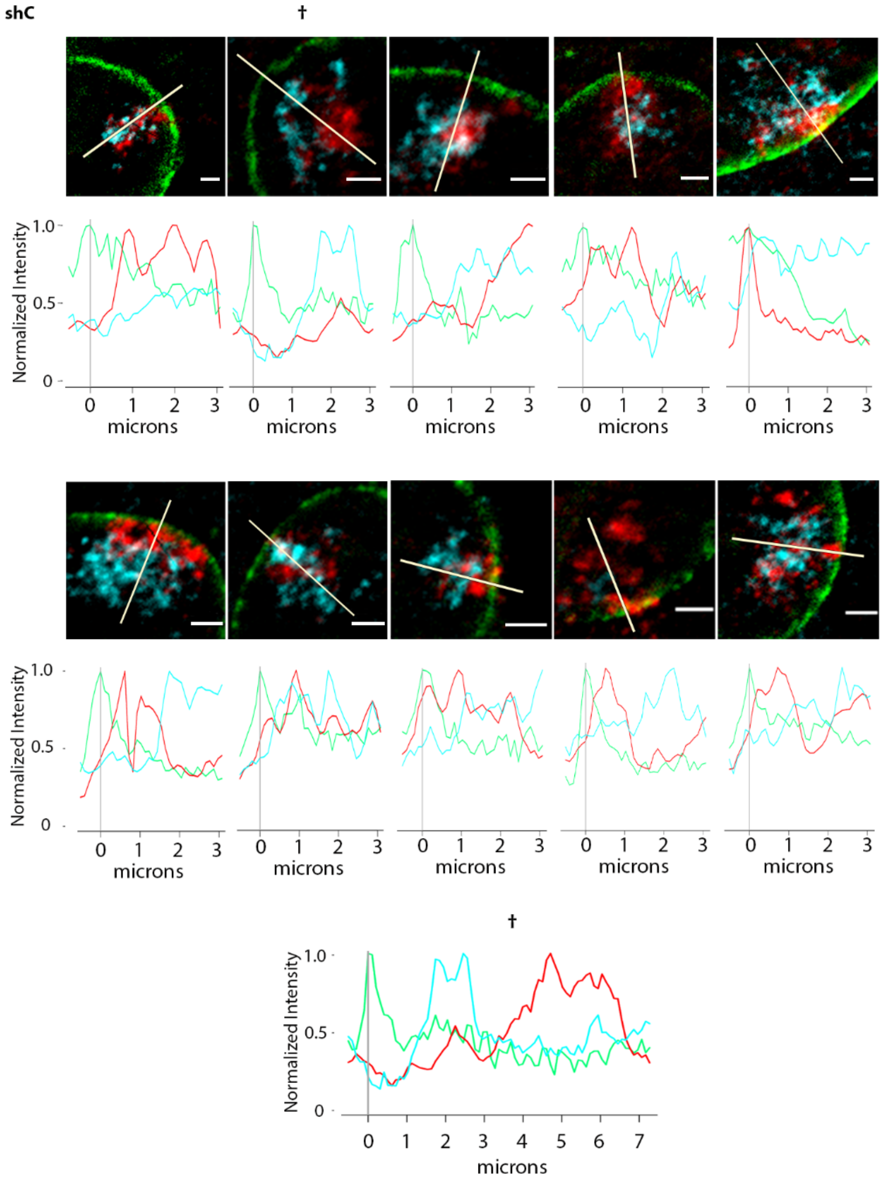

Fig S8: Knockdown of lamin C disrupts LAD self-aggregation and peripheral association Representative images of chromosome conformation paints, highlighting nonLADS (cyan) and LADs (red), and lamin B1 (green). Normalized fluorescence intensity histogram plots for chromosome 11 territories in knockdown of lamin C using a specific shRNA, plotted from the nuclear lamina (0µm) to 3µm into the nucleus. Territories marked with †(s) were plotted beyond 3µm to display the extent of LAD signals. The line each plot travels through is represented by a yfpwhite line. Scale bar = 2µm.

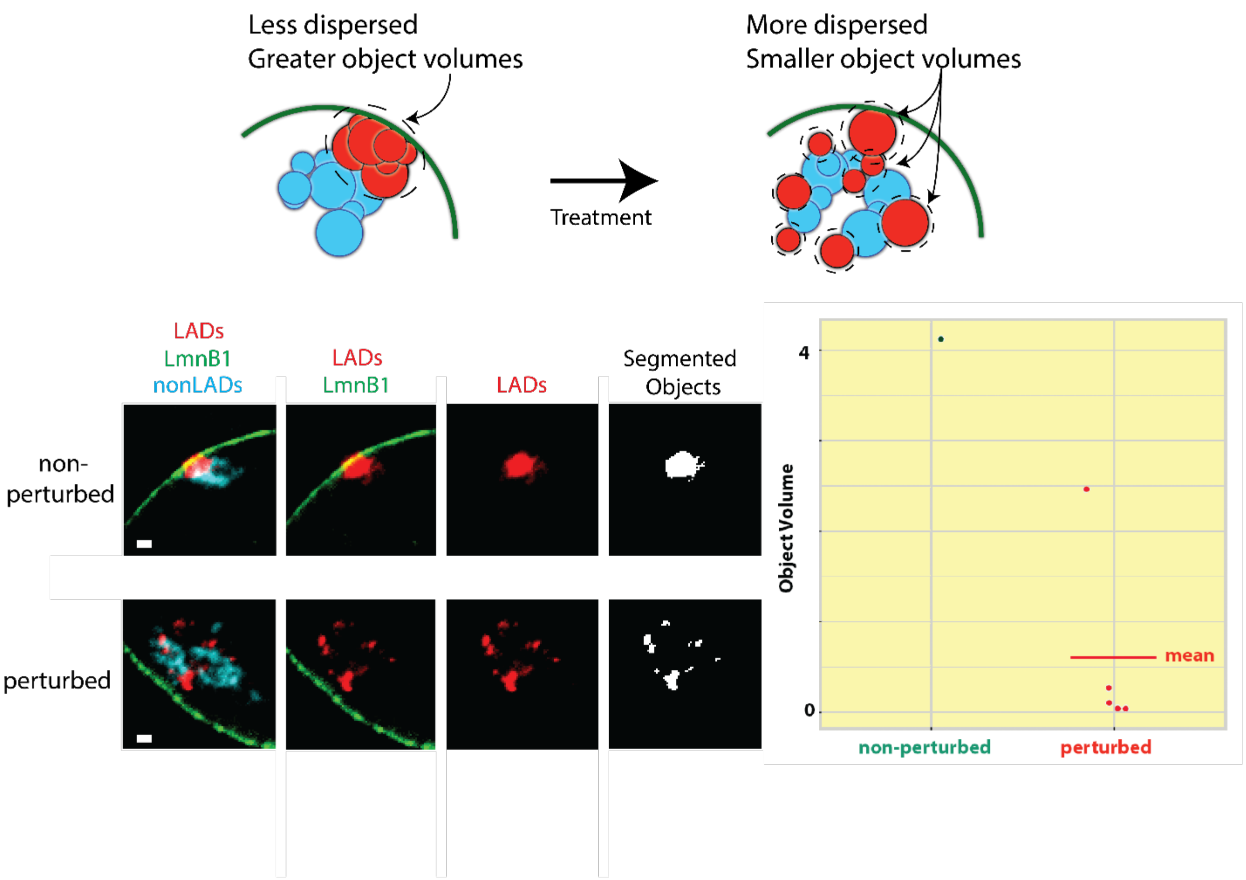

Fig S9: Illustration of LAD object segregation method

The top schematic depicts the conformation of an interphase chromosome in both non-perturbed (left) and perturbed states (right). In the non-perturbed state, LAD signals aggregate as a cluster are shown to exhibit loss of aggregation and increase in numbers and smaller sized "objects". On the bottom left, the method is illustrated on a single chromosome territory both in a non-perturbed state (top row) and perturbed state (bottom row). Segmentation is done on the LAD signal (red) and the segmented objects are pseudo-colored white. On the bottom right, volumes of the segmented objects are plotted. The volume of the object from the non-perturbed territory appears as a single point while objects from the perturbed territory are plotted as a spread of data points with reduced volumes, with the mean volume smaller than that of the non-perturbed territory.

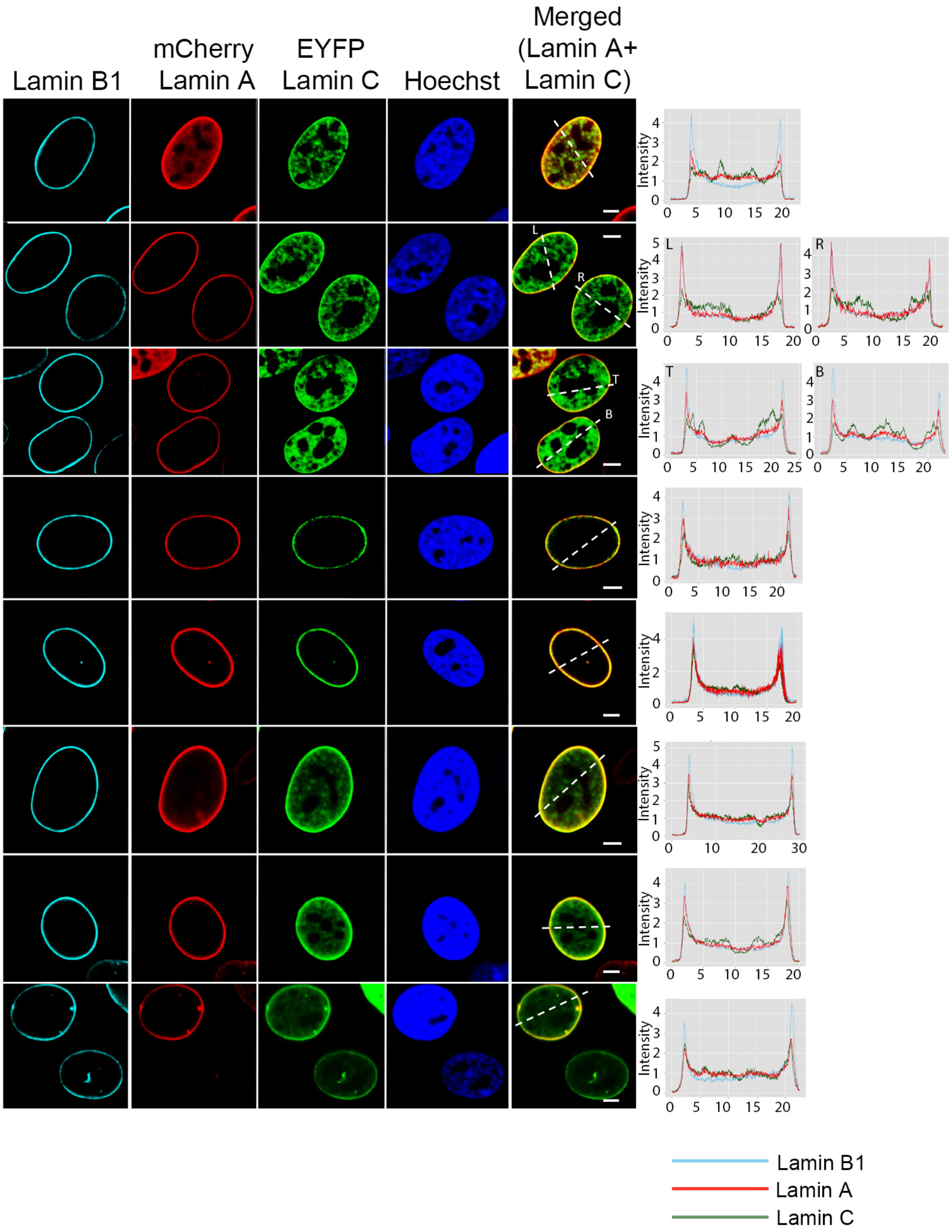

Lamin B1
mCherry Lamin A
EYFP Lamin C
Hoechst
Merged (Lamin A+ Lamin C)
Intensity
L
R
T
B
Lamin B1
Lamin A
Lamin C

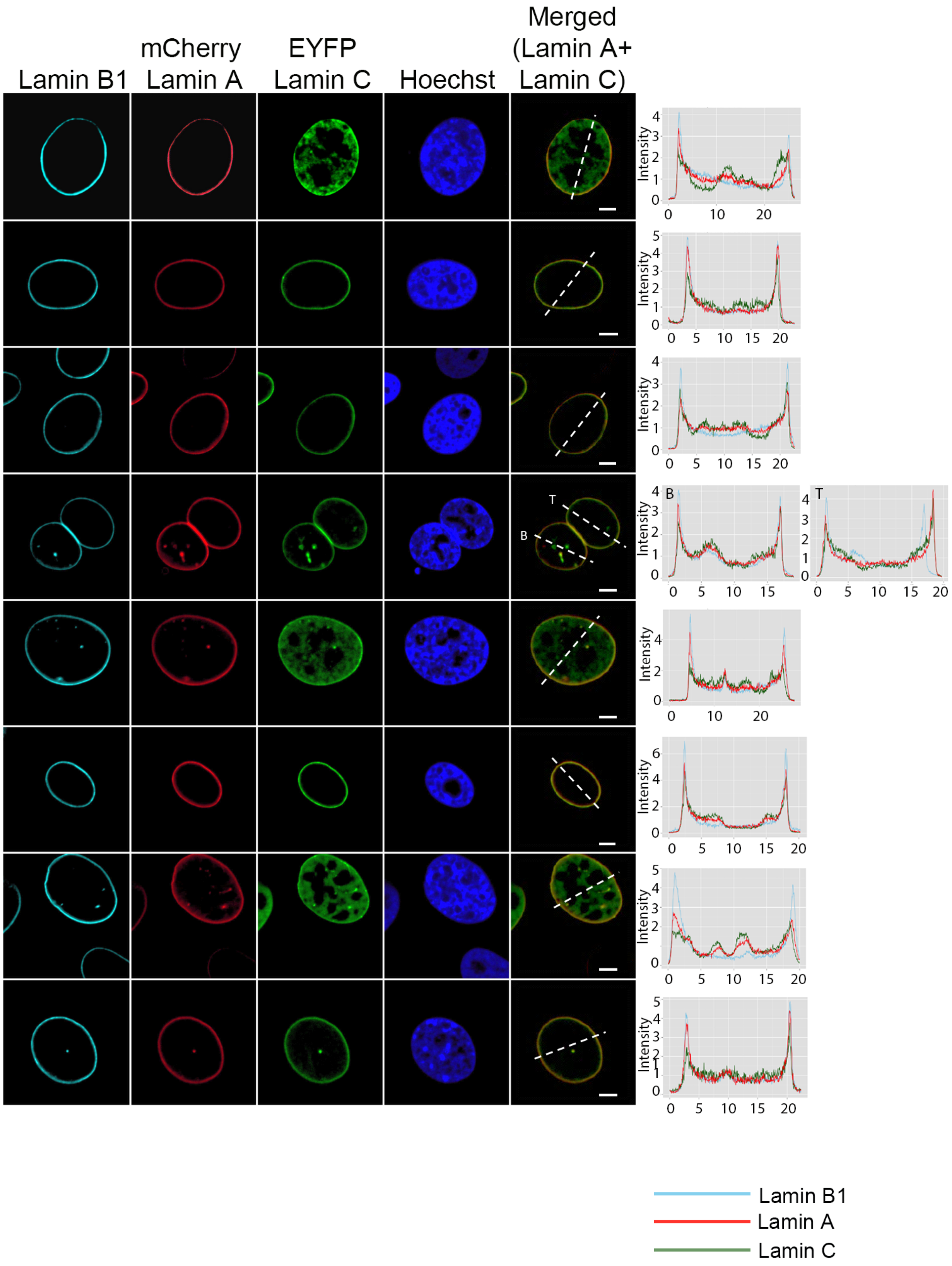

Lamin B1
mCherry Lamin A
EYFP Lamin C
Hoechst
Merged (Lamin A+ Lamin C)
Intensity
T
B
Lamin B1
Lamin A
Lamin C

Fig S10: All 3 lamins Isotype localize to the periphery during interphase
Representative images of interphase nuclei from unsynchronized cells (early G1-prophase) anti-lamin B1 (cyan), mcherry-lmnA (red), EYFP-lmnC (green) and Hoechst 33342 (blue). Normalized fluorescence intensity histogram plots for lamin B1 (cyan), lamin A (red) and lamin C (green) along the indicated white dotted line in merge. Scale bar = 5μm.

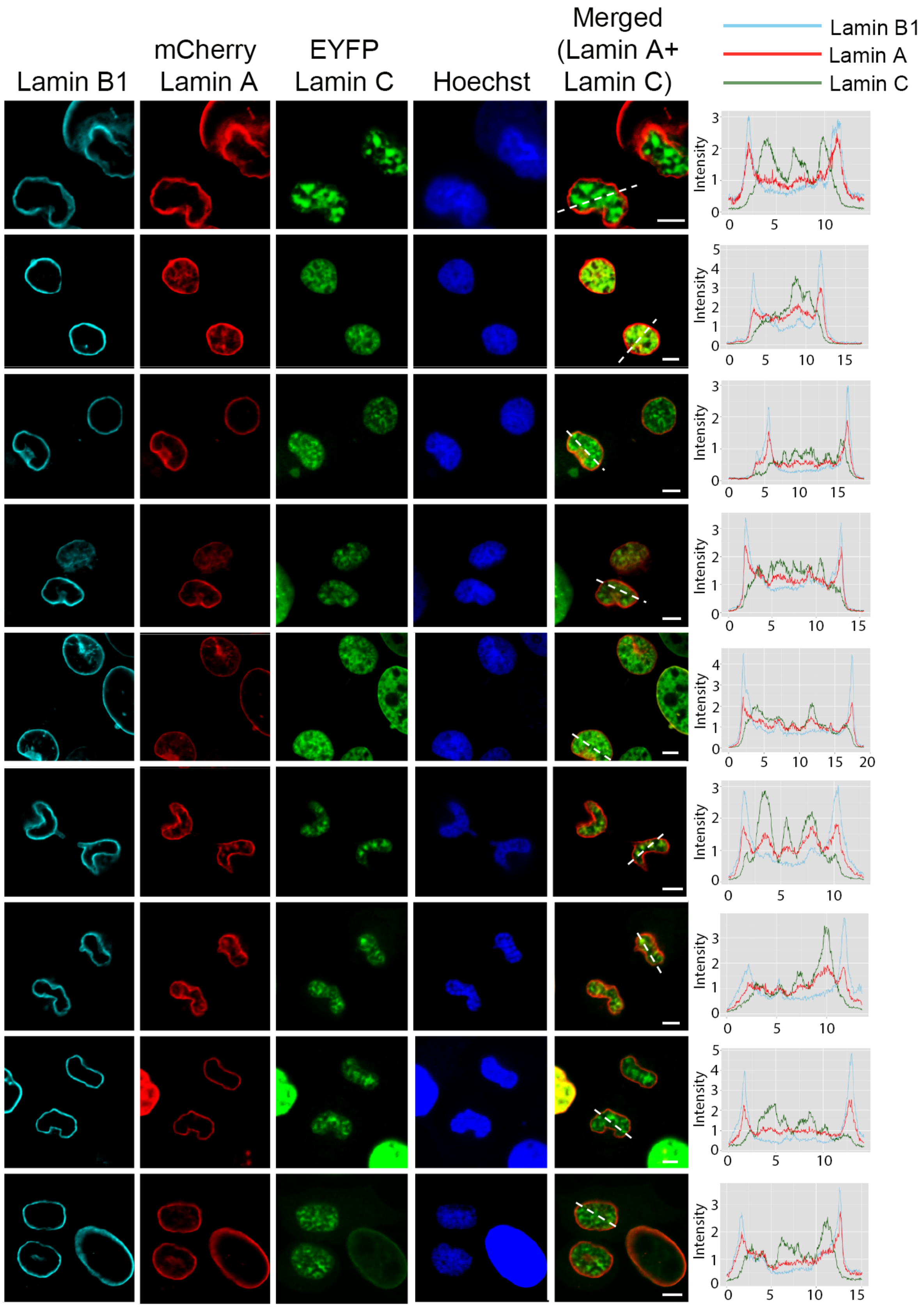

Lamin B1
mCherry Lamin A
EYFP Lamin C
Hoechst
Merged (Lamin A+ Lamin C)
Lamin B1
Lamin A
Lamin C
Intensity

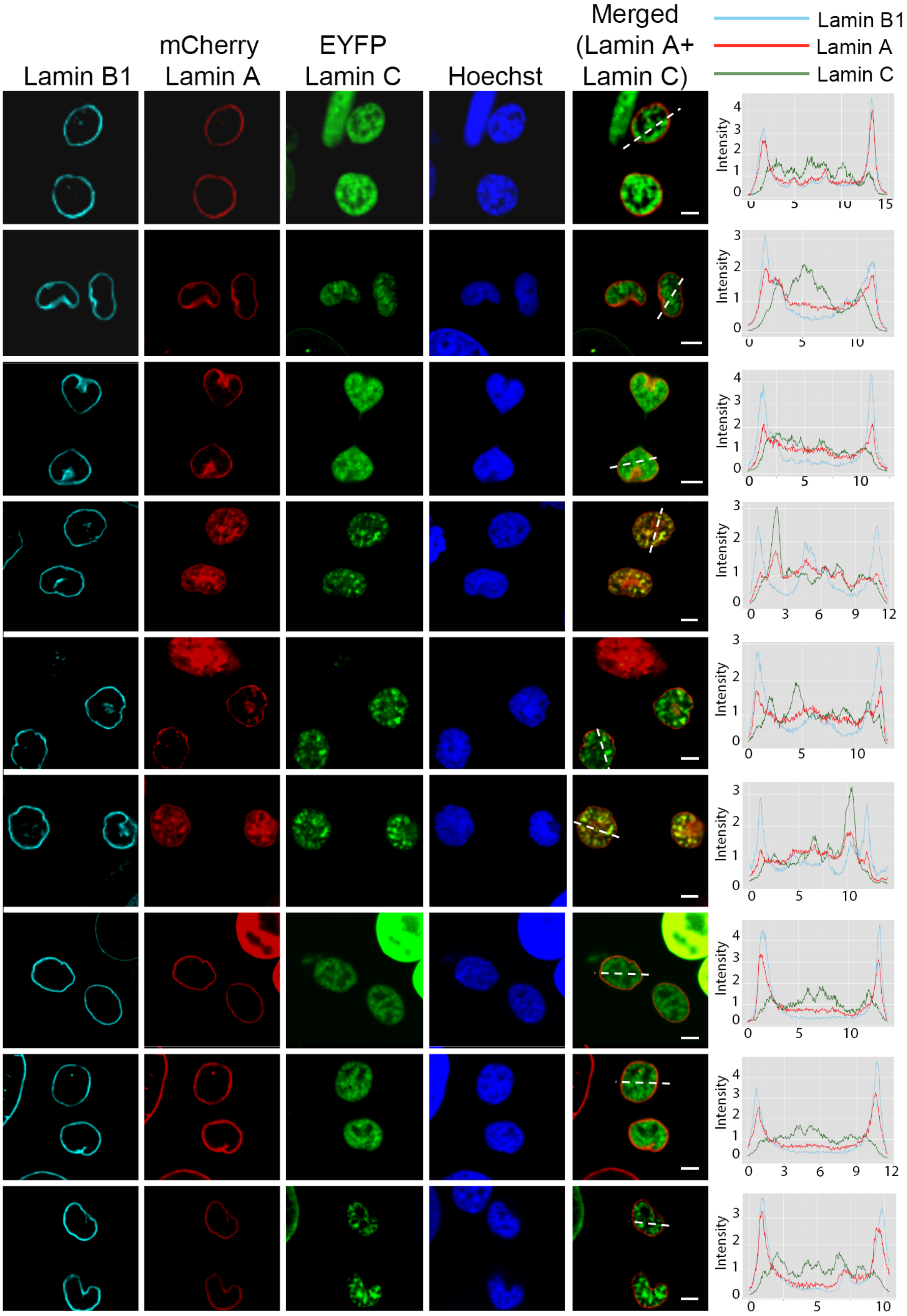

Lamin B1
mCherry Lamin A
EYFP Lamin C
Hoechst
Merged (Lamin A+ Lamin C)
Lamin B1
Lamin A
Lamin C
Intensity

Fig S11: Differential localization of lamin B, lamin A and lamin C at mitotic exit (telophase and early G1)

Representative images of telophase and early G1 nuclei anti-lamin B1 (cyan), mcherry-lmnA (red), EYFP-lmnC (green) and Hoechst 33342 (blue). Normalized fluorescence intensity histogram plots for lamin B1 (cyan), lamin A (red) and lamin C (green) along the indicated white dotted line in merge. Scale bar = 5μm.

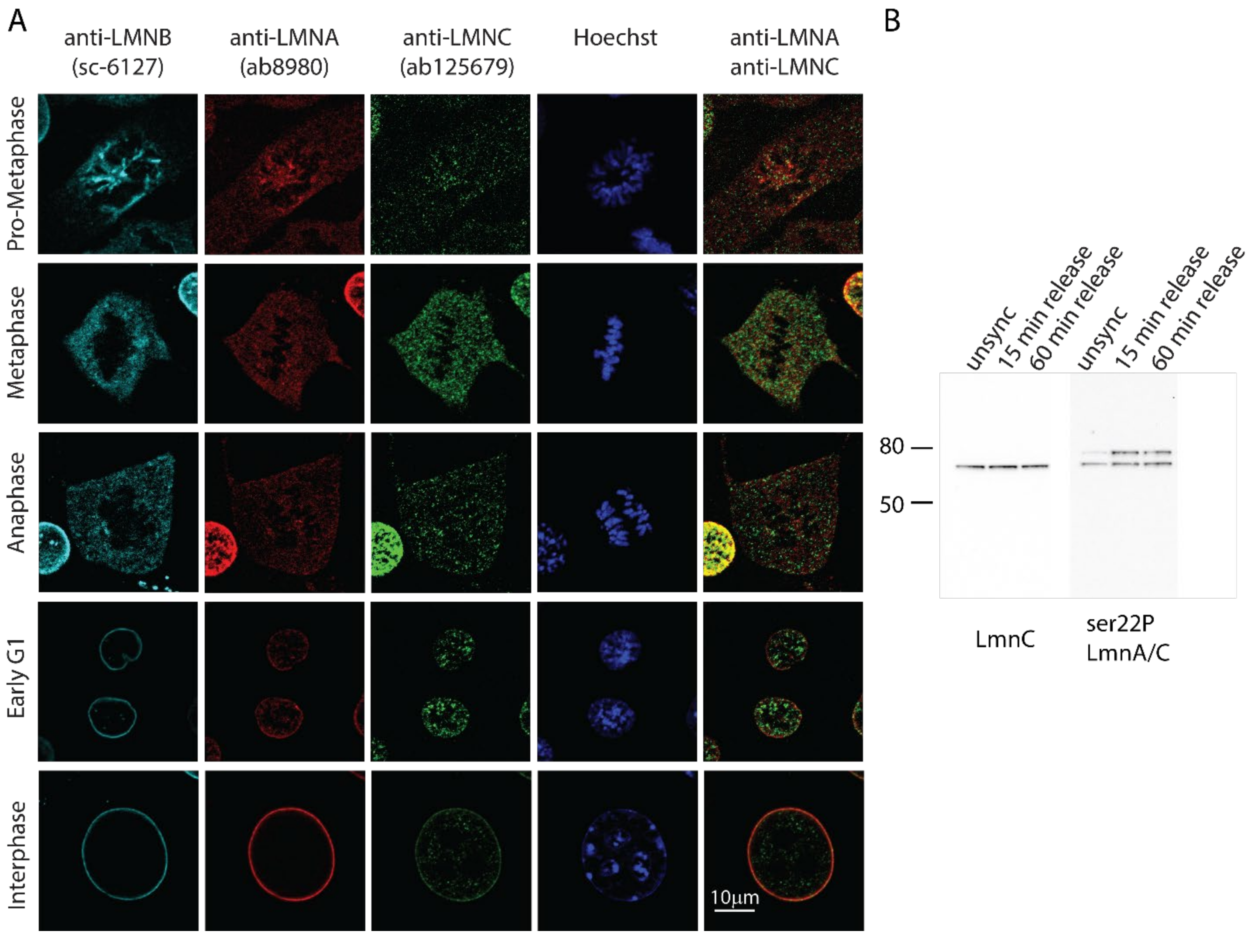

Fig S12: Antibody staining for lamin C shows the same localization as fluorescently tagged construct

(A) Representative image of MEFs during pro-metaphase, metaphase, anaphase, early G1 and interphase stained with antibodies to lamin B1 (sc-6127, cyan), lamin A (ab8980, red) and lamin C (ab125679, green) and counterstained with Hoechst (blue). Scale bar indicated by white line is 10µm. (B) Representative western blot showing the specificity of anti-lamin C even at low and high exposure levels.

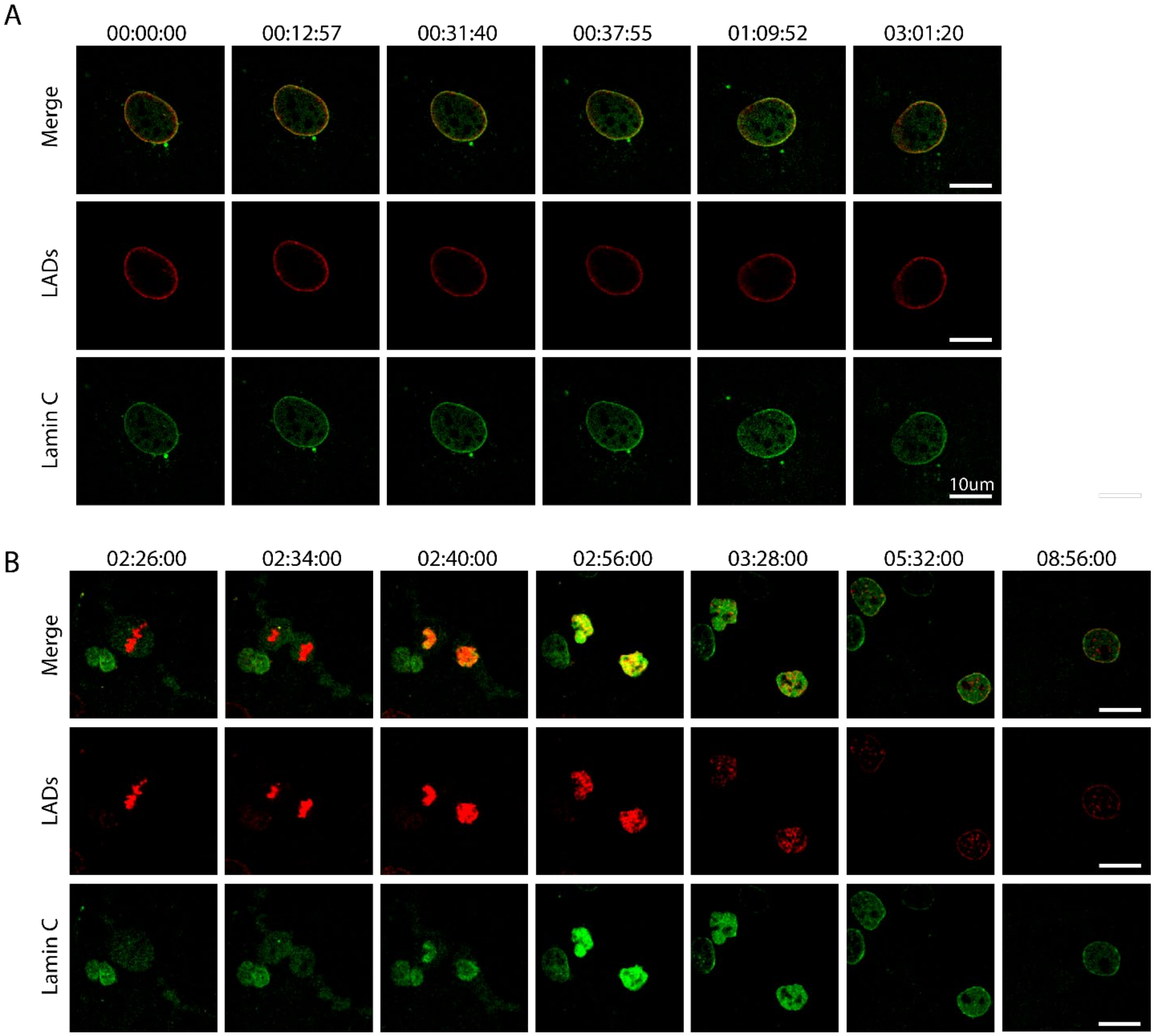

Fig S13: lamin C and LADs resolve concurrently during G1

(A) Still images from time lapse movie 4 of LADs (red) and EGFP-LmnC (green) during interphase shown over a similar time scale to movie 2. Scale Bar is 20μm. (B) Still images from time lapse movie 2 of LADs (red) and EGFP-LmnC (green) during mitosis. Scale bar is 20μm. Images were chosen to exemplify certain stages (metaphase, anaphase, telophase, early G1, partially resolved, fully resolved, and mid G1). This movie extends further in time than other movies showing how LAD configuration continues to become tighter to the lamina well into G1 phase.

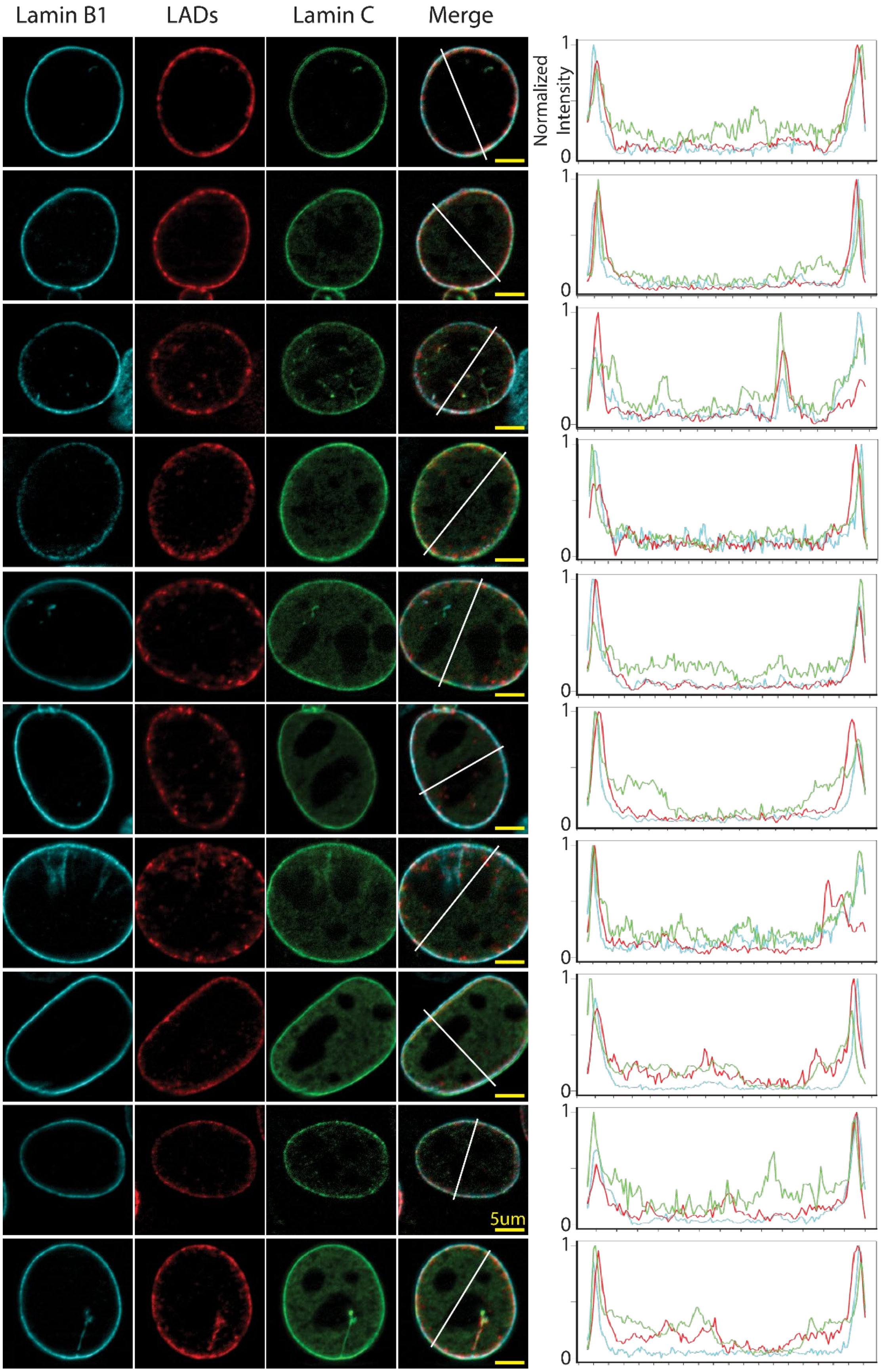

Lamin B1
LADs
Lamin C
Merge
Normalized Intensity
1
0
5um

Fig S14: LADs and lamin C show peripheral localization during interphase

Representative images of interphase nuclei anti-lamin B (cyan), m6A tracer (red) and EGFP-lmnC (green). Normalized fluorescence intensity histogram plots for lamin B1 (cyan), LADs (red) and lamin C (green) along the indicated white line. Yellow scale bar = 5μm.

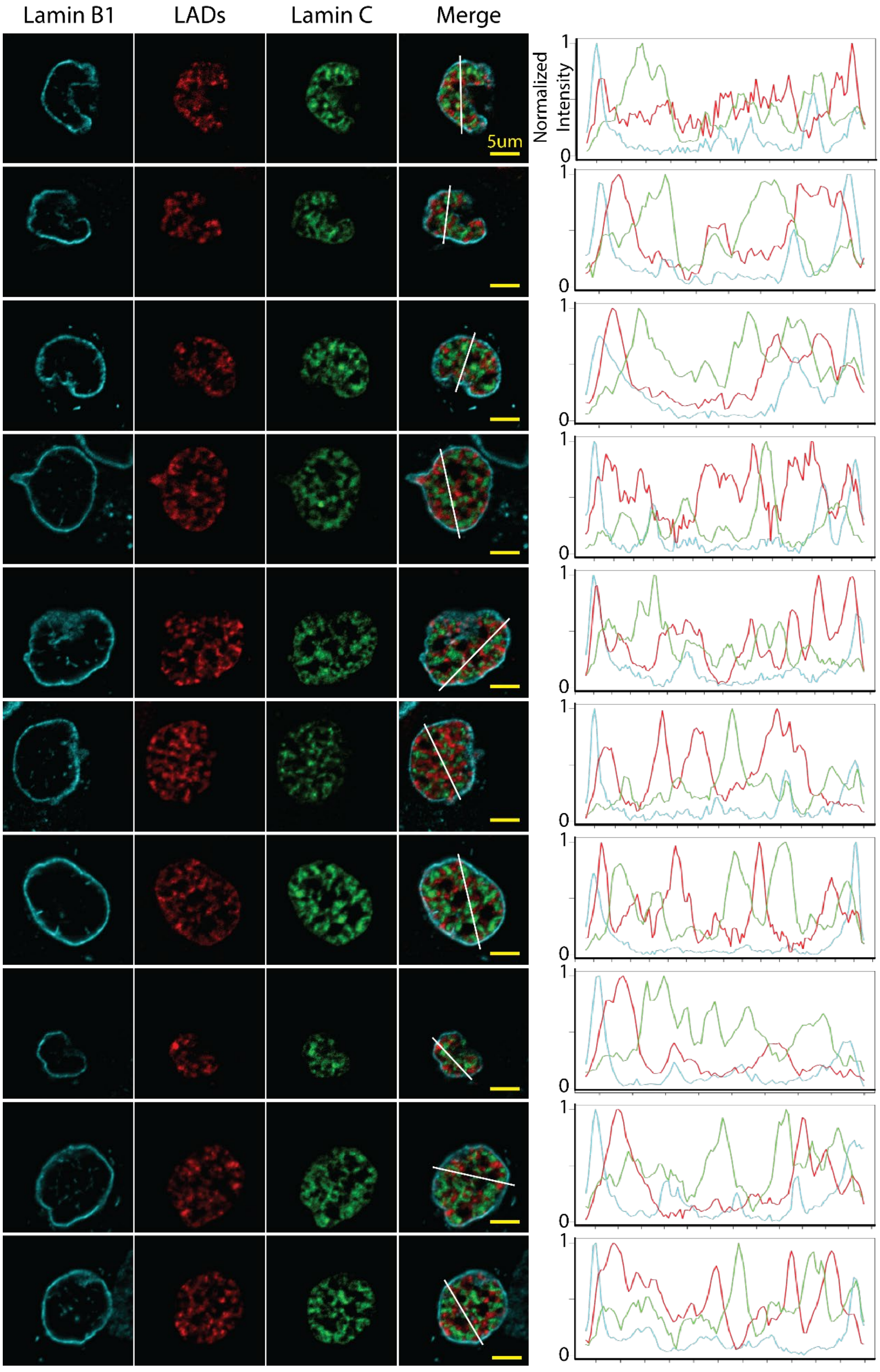
Lamin B1
LADs
Lamin C
Merge
5um
Normalized Intensity
1
0

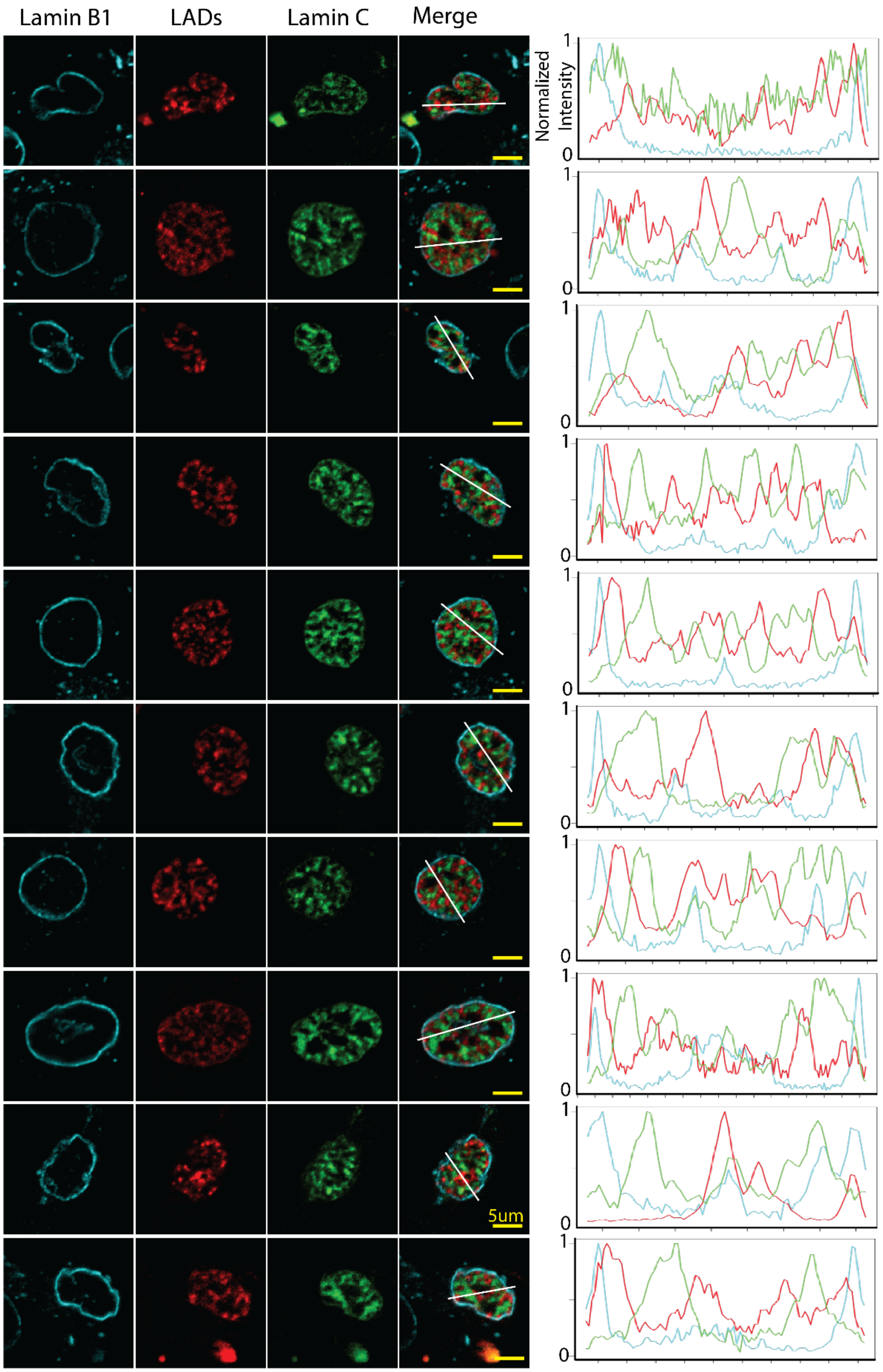

Fig S15: LADs and lamin C show nucleoplasmic localization during telophase/early G1 but do no colocalize

Representative images of telophase or early G1 nuclei: anti-lamin B1 (cyan), m6A tracer (red) and EGFP-lmnC (green). Normalized fluorescence intensity histogram plots for lamin B1 (cyan), LADs (red) and lamin C (green) along the indicated white line. Yellow scale bar = 5µm.

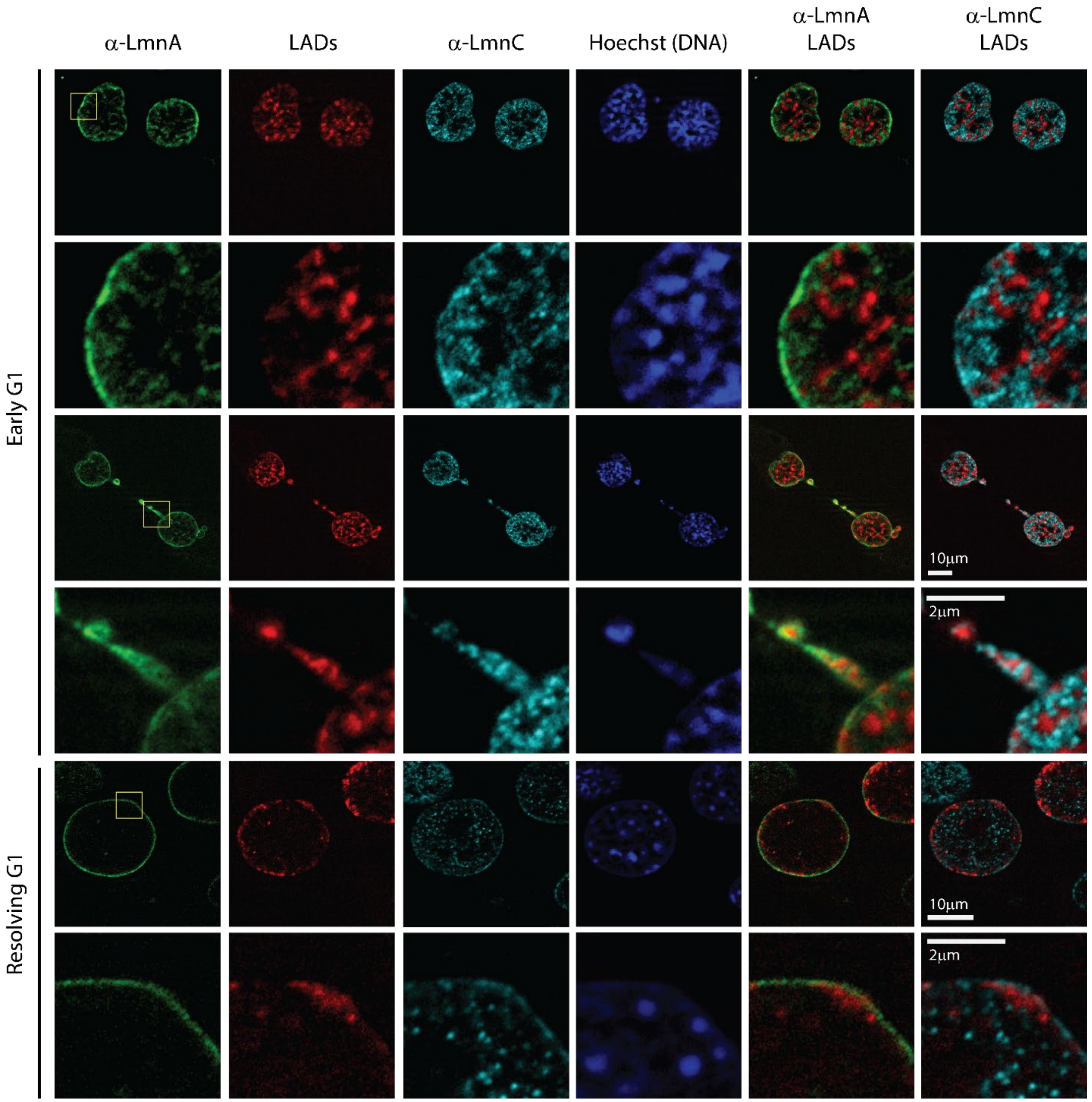

Fig S16: LADs and lamin C, not lamin A, are spatially, mutually exclusive during early G1 Representative images of early G1 nuclei and early-mid G1 (resolving interphase) nuclei with lamin A (green), m6A tracer (red), lamin C (cyan) and Hoechst 33342 (blue). In row 3, the spatial organization of lamins A, C and LADs highlights the specific exclusion of lamin C from LAD aggregates in early G1.

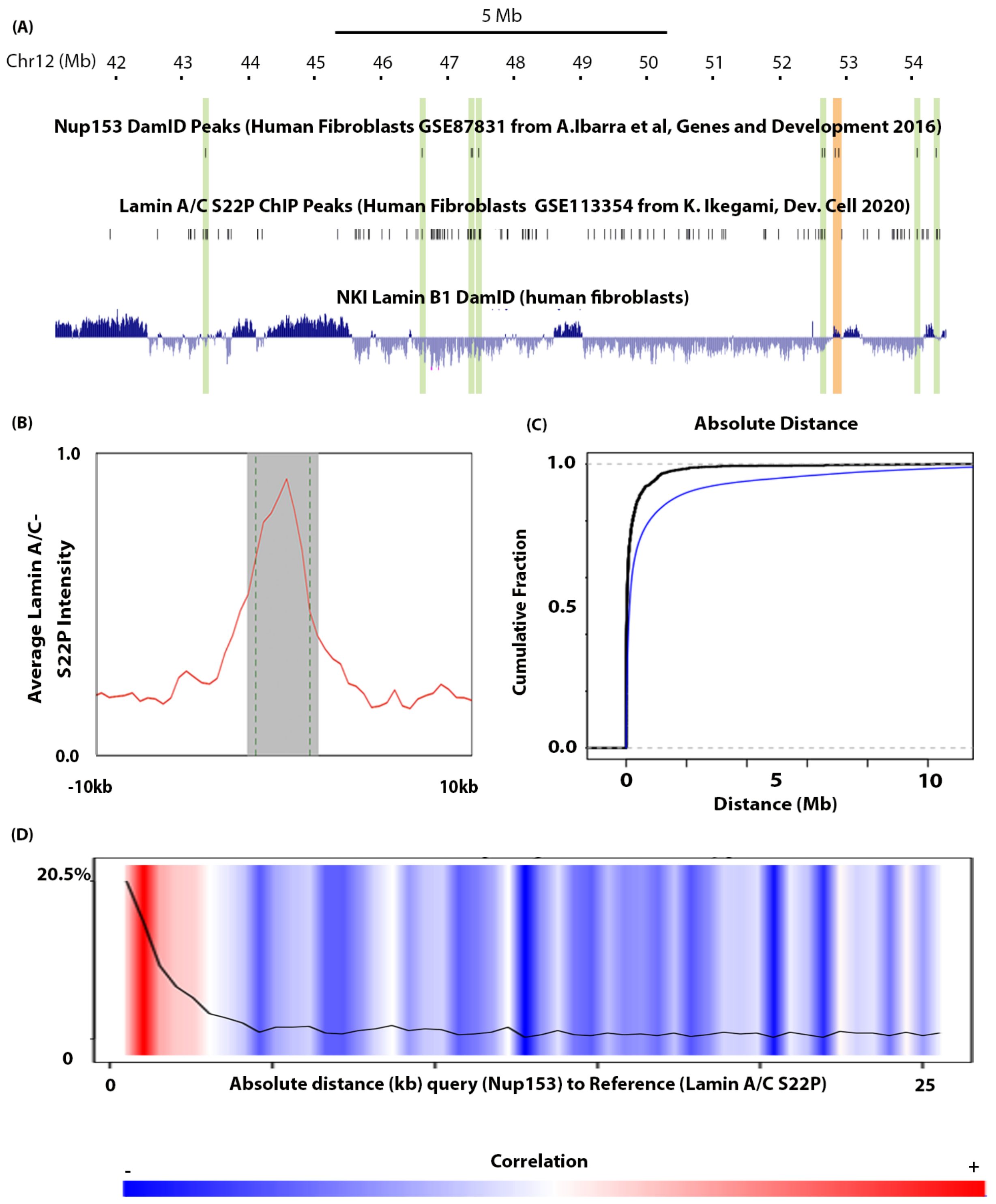

(A)
5 Mb
Chr12 (Mb) 42 43 44 45 46 47 48 49 50 51 52 53 54
Nup153 DamID Peaks (Human Fibroblasts GSE87831 from A.Ibarra et al, Genes and Development 2016)
Lamin A/C S22P ChIP Peaks (Human Fibroblasts GSE113354 from K. Ikegami, Dev. Cell 2020)
NKI Lamin B1 DamID (human fibroblasts)
(B)
1.0
0.0
Average Lamin A/C-S22P Intensity
-10kb
10kb
(C)
Absolute Distance
1.0
0.5
0.0
Cumulative Fraction
0
5
10
Distance (Mb)
(D)
20.5%
0
0
Absolute distance (kb) query (Nup153) to Reference (Lamin A/C S22P)
25
Correlation
-
+

Fig S17: Nuclear pore complexes are positionally correlated with S22P lamin A/C binding sites

(A) Nup153 DamID peaks, S22P lamin A/C peaks and Log_2 ratio plot of lamin B1 DamID from human fibroblasts are shown. Green shaded areas depict coincidence of Nup153 peaks with S22P lamin A/C peaks, orange shaded areas show non-overlapping, but proximal, Nup153 and S22P lamin A/C peaks. (B) Average S22P lamin A/C peaks intensity within 10kb of all Nup153 peaks shown. Dotted vertical lines (in red) indicate the mean Nup153 genomic interval size. Gray shaded area extends to 1 standard deviation from the dotted vertical lines. (C) An Estimator of the Cumulative Distribution Function (ECDF) plot of the cumulative fraction of Nup153 peaks occurring within an absolute distance from S22P lamin A/C peaks. The blue line depicts a distribution where there is no association between the datasets and the black line shows the actual distribution of the data. The positive difference in the area under the curve (AUC_{black} −AUC_{blue}) indicates that the Nup153 peaks are closer to S22P lamin A/C peaks than expected of a random distribution (blue), indicating a positive correlation. Jaccard Measure p−value : <0.02. (D) A graphical interpretation of the spatial relationships between Nup153 and S22P lamin A/C peaks. Nup153 peaks are depicted along the plot according to their distance to S22P lamin A/C peaks; the colors indicate deviation from the expected distribution with red being positively correlated and blue indicating a negative association. The overlay line indicates the density of Nup153 peaks at each absolute distance. The plot shows 20.5% of all Nup153 peaks directly overlapping S22P lamin A/C peaks, center to center, with most Nup153 peaks occurring within 2.5kb of S22P lamin A/C peaks. Jaccard test suggests Nup153 and S22P lamin A/C peak intervals overlap significantly more than expected by chance, Jaccard p-value < 0.02.

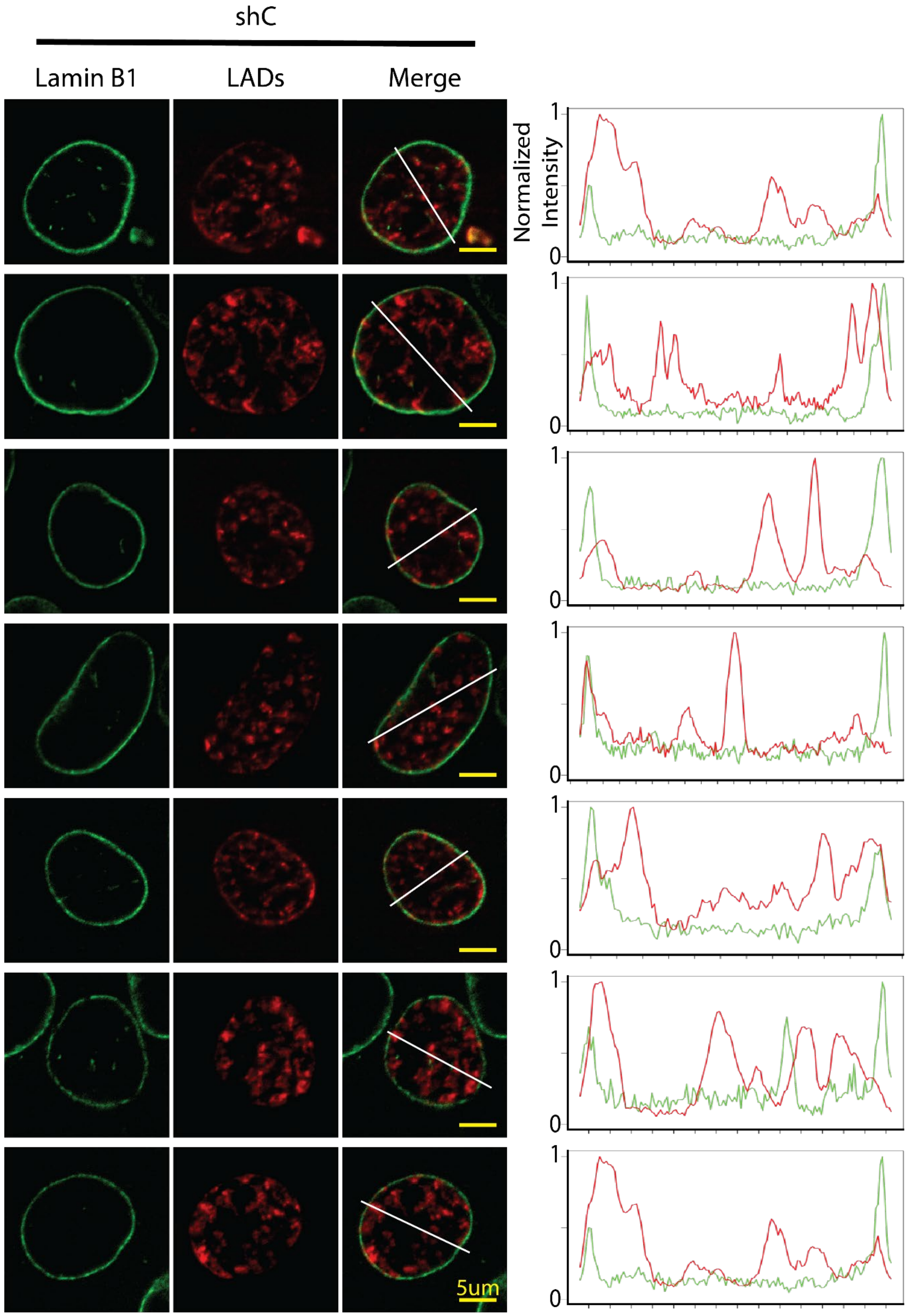
shC
Lamin B1
LADs
Merge
Normalized Intensity
1
0
5um

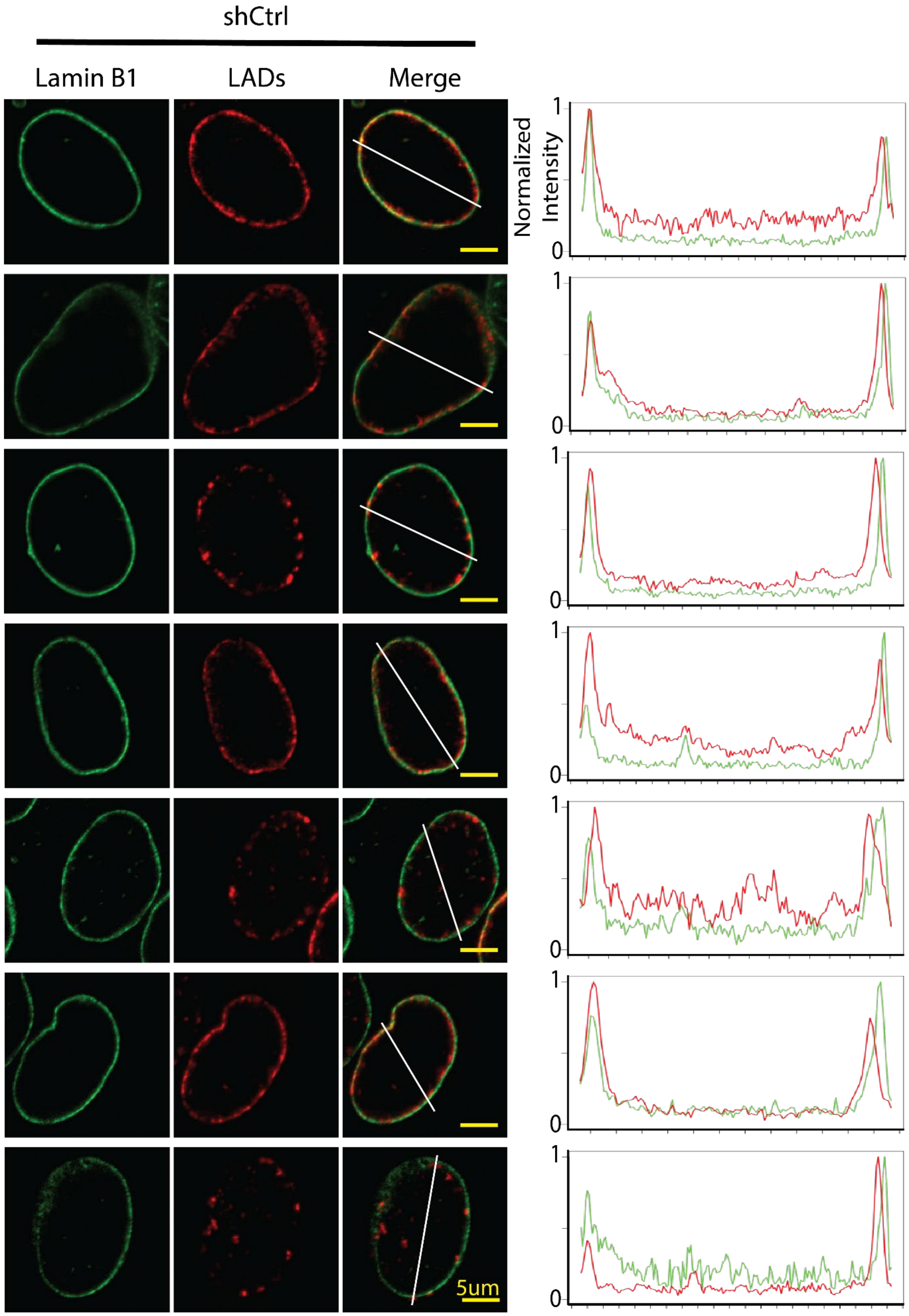
shCtrl
Lamin B1
LADs
Merge
Normalized Intensity
1
0
5um

Fig S18: LADs form network like structures after cell division in the absence of lamin C Representative images of cells with knockdown using a short hairpin RNA to a non-specific target, or lamin C in the m6A tracer system. Lamin B1 (green) and LADs (red). Normalized fluorescence intensity histogram plots for lamin B1 (green) and m6A tracer (red) along the indicated white line. Yellow scale bar = 5μm.
