# Supplementary material for: Lamin C is required to establish genome organization after mitosis
Source: Genome Biol. 2021 Nov 15;22:305. doi: 10.1186/s13059-021-02516-7 (PMC8591896; doi:10.1186/s13059-021-02516-7)
Supplement: Supplementary file 2 — Additional file 2. Contains images of full western blots [file 13059_2021_2516_MOESM2_ESM.pdf]

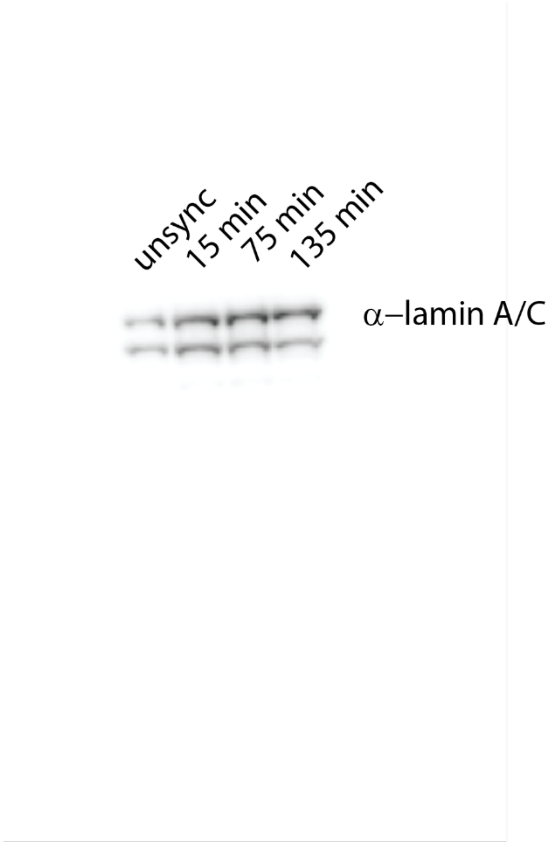

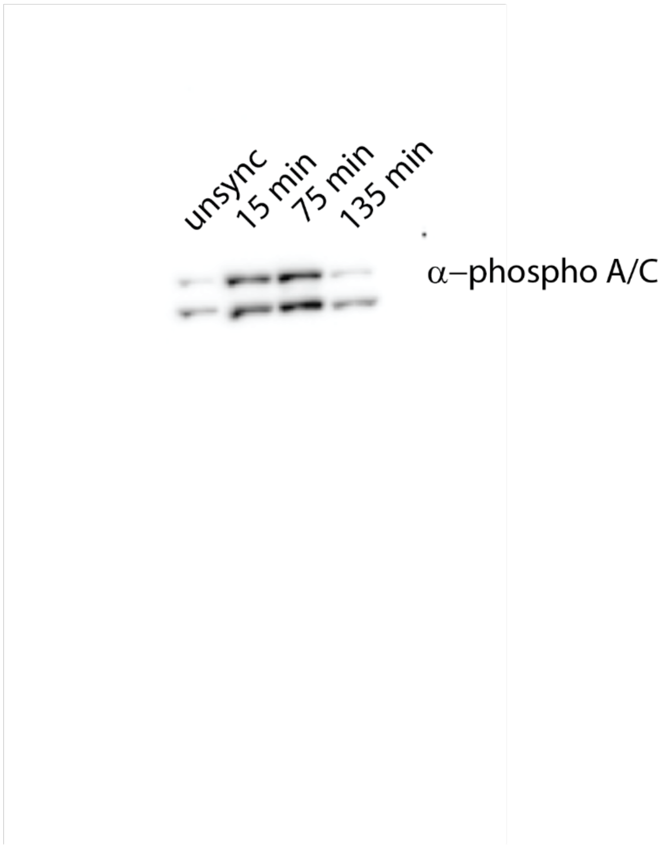

Western blot for Figure 5. Using the blot in the middle depicting anti-Lamin A/C for unsynchronized cells, 15 minutes release, 75 minutes release and 135 minutes release, respectively.

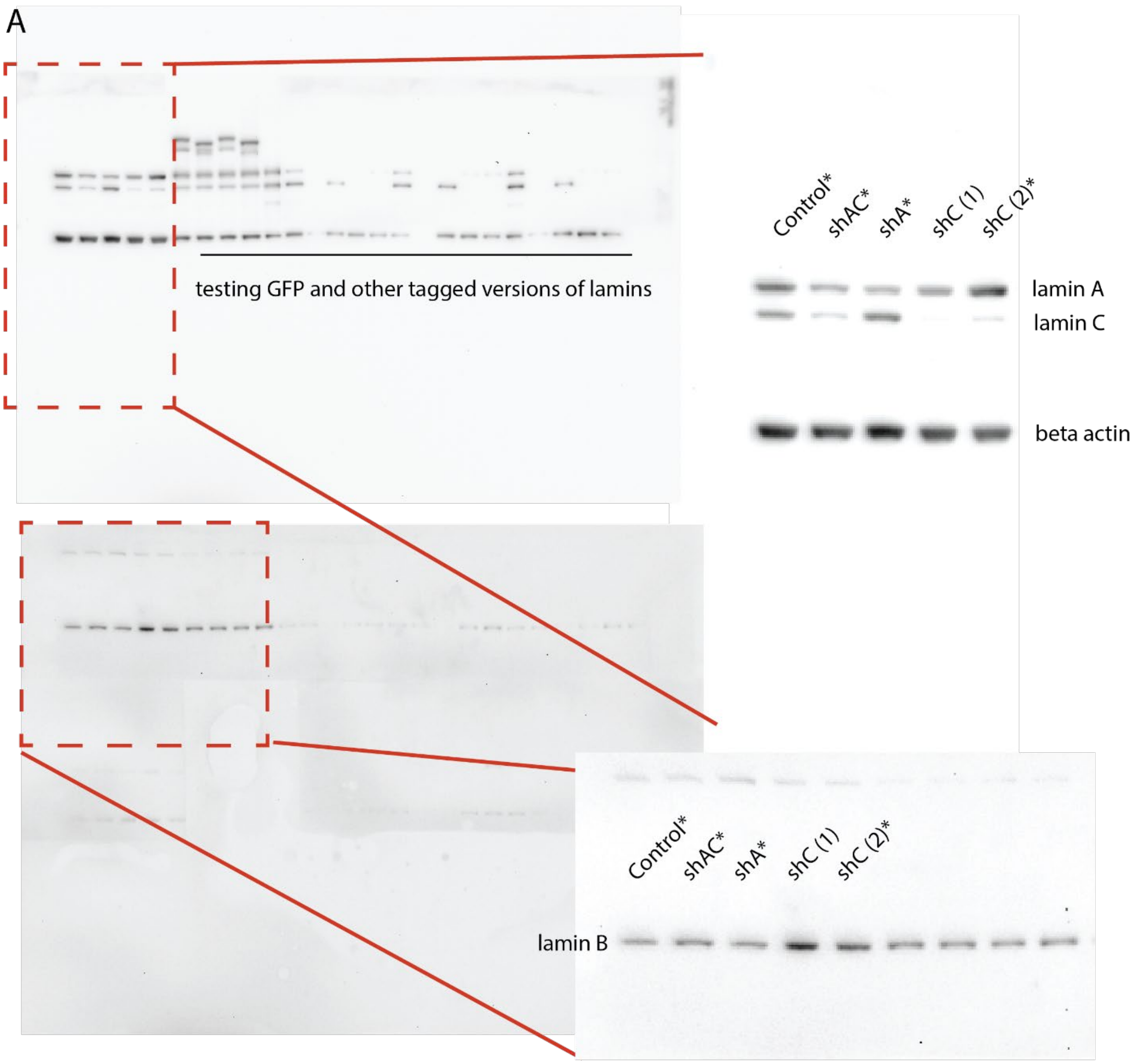

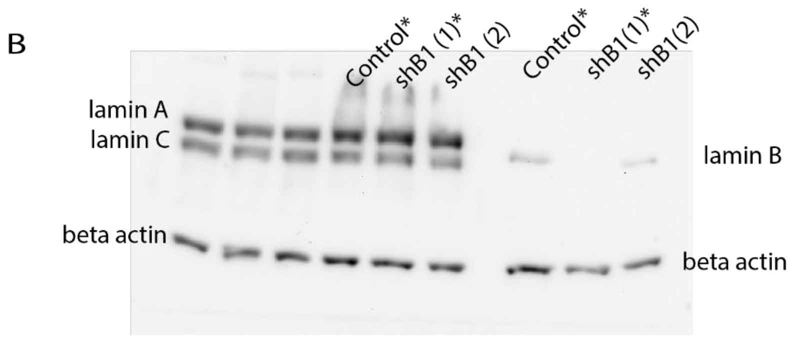

sh constructs denoted with a * were used in the study. shAC and shA were used in Harr et al., 2015, JCB

Western blot for Fig S1: (A) shRNA knockdowns for lamin A, lamin A/C, and lamin C ; (B) shRNA knockdowns form lamin B1. "X" indicates the lamin isotype targeted, * indicates the shRNA construct used in this study. Blots are labeled across the top with the shRNA construct used and specific bands detected by immuneblot are indicated on the sides. Where warranted, sections of relevant blots are magnified. Panel A has multiple experiments on the western, including labeled lamin proteins.

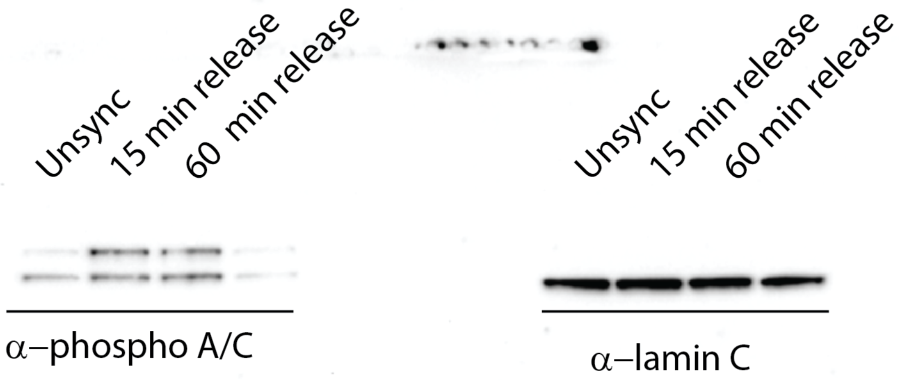

Western blot for Fig S12. Using first three lanes of each blot for the figure, depicting anti-Serine-22 Phosphorylation (left) and anti-lamin C (right) for unsynchronized cells, 15 minutes release and 60 minutes release, respectively.
