# Supplementary material for: Lamin C is required to establish genome organization after mitosis
Source: Genome Biol. 2021 Nov 15;22:305. doi: 10.1186/s13059-021-02516-7 (PMC8591896; doi:10.1186/s13059-021-02516-7)
Supplement: Supplementary file 3 — Additional file 3. Review history [file 13059_2021_2516_MOESM3_ESM.docx]

Review History

**First round of review**

**Reviewer 1**

**Are you able to assess all statistics in the manuscript, including the appropriateness of statistical tests used?** Yes, and I have assessed the statistics in my report.

**Comments to author:**

In this manuscript, Wong and colleague address the role of nuclear lamins A and C (which are two splice isoforms of the same gene) in the organization of the genome after mitosis. They first find marked differences in the localization of lamins A and C upon nuclear reformation post-mitosis and into G1; they find that lamin A associates with the nuclear envelope in telophase (which was known) whereas remarkably, lamin C is detected in the vicinity "LAD clusters" (LADs in the previous cell cycle) in the nucleoplasm. Targeting of lamin C to the nuclear envelope occurs well into G1 concomitantly with the detection of LADs at the nuclear periphery. Depletion of lamin C, but not A, disrupts LAD association with the nuclear periphery and chromosome organization (compaction state) at the periphery. The authors conclude that lamin C specifically is required for post-mitotic LAD organization and chromosome organization. They then speculate on a model of a putative role of lamin C in genome organization.
This is an interesting paper which brings new elements in the long-debated question of whether lamins A and C play distinct roles (or not) in the radial organization of chromatin. The results are solely based on imaging data, but a strength of the approach is the complementarity of techniques, including FISH (chromosome conformation paints), immunostainings, tagged chromosomal insertions and a LAD tracer system, in fixed and/or live cells.  

A number of issues however arise and should be examined or clarified.
1.     The DamID data show strikingly no difference in LADs regardless of which lamin is down-regulated (Fig. S2, S3). This is despite the marked disorganization of chromatin / LADs near the nuclear periphery after shLaC shown by FISH. How to the authors reconcile these observations? They raise the point (p4) but do not provide elements of answer (and leave the reader "hanging" here….).  See also point 6) below.  

2.     The section on LAD / chromatin sub-domain organization in wt and sh cells (Fig 1, Fig S4-8) suffers from inaccurate (and somewhat subjective) quantification: a) the concept of "disrupted organization" (Fig. 1B) is ill-defined because variations in chromosome organization between cells, and should be address; b) representation of chromosome / LAD morphology along a line is not appropriate because the end-result will obviously depend on where the line is drawn. Since the FISH images show heterogeneity in the structures,  refined approaches should be taken when quantifying chromosome/domain morphology, such as computation of volume, gyration radius, granularity or other. There is a lot more information behind the images than what a line-graph can represent. The effect of shLaC relative to shLaA and shLaA/C would be a lot more convincingly established more multiple measurements.

3.     The authors should quantify the time-difference in the targeting of lamin A, or lamin C, AND LADs to the NE their time-laps analysis. This should be possible from movie data. It would be interesting to know how much time-variation there is in the LAD targeting process to the NE between cells. Chromatin imaging is a strength of the Reddy lab and in my opinion could be taken to another level in an informative manner in this manuscript.

4.     How does LAD targeting to the NE take place? It is unclear to me whether a lamin C + LAD complex is targeted "together" to the NE, or whether both an targeted to the NE separately to end up there concomitantly. Addressing this question experimentally would significantly add to our understanding of the establishment of LADs. At the very least, this would be addressed in the discussion.

5.     LADs have been reported to be similar to NADs, based on sequence-based data. In this context, it would be relevant to know whether the nucleoplasmic lamin C+LAD "complexes" are found near nucleoli, with perhaps come even remaining there later in G1.

6.     What would be post-mitotic implications of the differential lamin A vs lamin C association with the NE? For example, are there lamin C- and lamin A-specific LADs? The authors suggest from their data that lamin A and C may contribute differently to the organization of chromatin there, a view supported by their references to the structural arrangement of lamins A, C (and B) filaments. Yet, DamID after sh-lamins B1, A, C or A/C show no difference (point 1). Are there nevertheless lamin subtype-specific LADs?

7.     While the data presented are overall solid, the discussion and model are in my opinion far too speculative. The model is also busy, making it somewhat unclear. For example, there is no evidence from the data that "chromatin loops", or "CTCF" of NPC components or lamin S22P is involved in their observations. Similarly, there are no data supporting a targeting of  promoters or enhancers to the NE mediated by lamin A S22P. I also have a scale issue problem with the model, "mid G1 - G1/S" diagram, with an "active chromatin loop" being associated with a nuclear pore in (?) / outside (?) a LAD. At the same time, there some "lamins A, B & C" in a lamina structure…. There is a scale discrepancy between the "loop" and the "LADs" represented. There is, I think, no evidence that chromatin targeting to the NE would be occur at the level of "loops", is there? I can see how some of the ideas are interesting to put forward, but I would refrain from being so speculative in this particular, data-based, manuscript.

Minor:
Fig 1B, 2B, 5B etc: unclear what the * and P val are related to (through it can be guessed). Please clarify in the legends.

**Reviewer 2**

**Are you able to assess all statistics in the manuscript, including the appropriateness of statistical tests used?** There are no statistics in the manuscript.

**Comments to author:**

This very interesting manuscript from the Reddy laboratory explores the role that A-type lamins play in defining the peripheral association of LADs. In particular, they demonstrate that the two major A-type lamin isoforms, lamin A and lamin C, are not functionally equivalent. This work represents an important addition to a body of evidence that has been slowly accumulating over the last decade suggesting that lamins A and C may have different, albeit overlapping, roles in defining nuclear organization. The evidence for this runs the gamut from mouse genetics to nanoscale microscopy, both light and EM.

The most important take-home message from this manuscript is that is that it is lamin C, not lamin A, that mediates the association of LADs with the nuclear lamina and inner nuclear membrane. This conclusion is based upon a series of knockdown experiments in which they use three independent methods to track LAD distribution, chromosome painting, chromosome insertion of tandem lacO repeats and DamID-based LAD tracer. The question obviously arises as to how these two very closely related proteins can exhibit functional differences in terms of their interactions with LADs. The authors provide intriguing evidence that these differences arise during the late stages of mitosis where lamins A and C exhibit clear differences in behavior. Lamin A appears to be recruited to the nuclear periphery in telophase ahead of lamin C. The latter remains largely nucleoplasmic, as do LADs, in to early G1. The implication is that the later recruitment of lamin C establishes a permissive environment for LAD association with the nuclear envelope. It is also consistent with studies that have suggested that lamins A and C appear to form separate filament networks. This mitosis observation suggests a possible mechanism for the segregations of lamins A and C into separate (or maybe partially separate) networks.

My only concern is with respect to the mitotic studies. I am wary about the use of large N-terminal tags on lamins and the effects that these might have on filament assembly. One way around this would be to use A vs C specific antibodies. Indeed, the authors do take advantage of an antibody "purportedly" specific for lamin C (Fig. 12). This makes me wonder whether the authors have some concerns about the validity of this reagent. It would be nice if they could include a western blot to demonstrate that it is actually C-specific.

The alternative approach to this mitotic question would be to employ short tags such as Flag, Myc or HA on the lamin A versus C N-terminus simply to confirm that lamin A is indeed recruited to the NE ahead of lamin C. This could be accomplished quickly on fixed unsynchronized cell populations.

Finally, there may be something interesting going on in Supplemental Figure 10b. There are a number of cells in which there is a large amount of nucleoplasmic lamin C. From the Hoechst staining it appears to me that these might be very early prophase cells (I can't be certain of this from the images, but I am very suspicious). If this is the case, then it would appear that lamin C is being released from the lamina ahead of lamin A at the onset of mitosis.

In conclusion, this is a well-documented and well-controlled study that should merit publication with only a few minor additions.

**Authors Response**

**Point-by-point responses to the reviewers’ comments:**

**We would like to very sincerely thank the reviewers for the time and care spent reviewing our manuscript. Your comments really helped make this study even stronger. We apologize for the delayed resubmission, but with Covid restrictions and effects on personal schedules as well as both first authors now in different laboratories, we had some hurdles to overcome. We have added a new author to the manuscript who did most of the benchwork-related additions. We have now included isotype specific antibody data via IF and have verified the specificity of the lamin C antibody. In addition, we add new data showing that the nucleoplasmic lamin C pool is highly correlated with ser-22 phophorylated lamin A/C both by IF and via western blot. We have also performed the volumetric analyses requested by reviewer 1 to come up with a “dispersion index” to enhance and further validate the chromosome organization disruptions we observe in the absence of lamin C (or lamin A/C). We hope that the additions we have made to the text and the additional data included answer your concerns and questions. We have included your original comment followed by our response in a bulleted section below it.**

In this manuscript, Wong and colleague address the role of nuclear lamins A and C (which are two splice isoforms of the same gene) in the organization of the genome after mitosis. They first find marked differences in the localization of lamins A and C upon nuclear reformation post-mitosis and into G1; they find that lamin A associates with the nuclear envelope in telophase (which was known) whereas remarkably, lamin C is detected in the vicinity "LAD clusters" (LADs in the previous cell cycle) in the nucleoplasm. Targeting of lamin C to the nuclear envelope occurs well into G1 concomitantly with the detection of LADs at the nuclear periphery. Depletion of lamin C, but not A, disrupts LAD association with the nuclear periphery and chromosome organization (compaction state) at the periphery. The authors conclude that lamin C specifically is required for post-mitotic LAD organization and chromosome organization. They then speculate on a model of a putative role of lamin C in genome Organization. This is an interesting paper which brings new elements in the long-debated question of whether

lamins A and C play distinct roles (or not) in the radial organization of chromatin. The results are solely based on imaging data, but a strength of the approach is the complementarity of techniques, including FISH (chromosome conformation paints), immunostainings, tagged chromosomal insertions and a LAD tracer system, in fixed and/or live cells. A number of issues however arise and should be examined or clarified.

Reviewer #1

1. The DamID data show strikingly no difference in LADs regardless of which lamin is down-regulated (Fig. S2, S3). This is despite the marked disorganization of chromatin / LADs near the nuclear periphery after shLaC shown by FISH. How to the authors reconcile these observations? They raise the point (p4) but do not provide elements of answer (and leave the reader "hanging" here....). See also point 6) below.

- This has been discussed in the text: “...While this was initially surprising, especially given our earlier findings that lamin A/C is required to organize regions to the lamina, we realize that an inherent limitation of DamID and related techniques is that data are aggregated from millions of cells, potentially obscuring true differences that would be detectable at the level of individual cells^6,9,46^...” This is something we have noticed in omics based approaches. Teh way DamID works is to amplify DamLMNB DNA and DamOnly DNA for normalizing. Any “above expected” interaction in DamLMNB will show as positive signals and since DAmID (like most ChIP-seq) is a binary measure of interactions, these will show up as positives. Given that these are shRNA experiments, we expect that there is enough signal from DamLMNB1 interactions to keep the maps “the same”. OF special interest, The Van Steensel and Goldman groups found in ES cells that LADs form in the “absence of lamins” yet when Zheng et al. did a quantitative analysis of that data (making an assumption that such data could be considered anything other than binary) they did find differences in LAD organization. In addition, the Van Steensel group using single cell DamID found that there is considerable cell-cell variability in some LAD regions that cannot be detected by normal DamID (although the limitation of this technique is the lack of single cell normalization and resolution issues). In summary, we do not find this to be very surprising after all.

2. The section on LAD / chromatin sub-domain organization in wt and sh cells (Fig 1, Fig S4-8) suffers from inaccurate (and somewhat subjective) quantification: a) the concept of "disrupted organization" (Fig. 1B) is ill-defined because variations in chromosome organization between cells, and should be address; b) representation of chromosome / LAD morphology along a line is not appropriate because the end-result will obviously depend on where the line is drawn. Since the FISH images show heterogeneity in the structures, refined approaches should be taken when quantifying chromosome/domain morphology, such as computation of volume, gyration radius, granularity or other. There is a lot more information behind the images than what a line-graph can represent. The effect of shLaC relative to shLaA and shLaA/C would be a lot more convincingly established more multiple measurements.

- **We have included a computational analysis of the sub-domain organization in wt and sh cells. This method segments LAD signals into objects irrespective of size (above a certain minimal setting used across all images). We show in the new version of figure 1 (Fig. 1 D) that conditions producing overt disruptions in chromosome organization yielded an increase incidence of “LAD-lets” characterized by more objects with smaller volumes as a result of loss of LAD-LAD interactions. Together with the line scans and TCIS data, this new analysis of our CCP data suggest to us that depletion of lamin C affects chromosome organization by causing LAD regions to become untethered from the periphery and also by disrupting LAD-LAD interactions.**

3. The authors should quantify the time-difference in the targeting of lamin A, or lamin C, AND LADs to the NE their time-laps analysis. This should be possible from movie data. It would be interesting to know how much time-variation there is in the LAD targeting process to the NE between cells. Chromatin imaging is a strength of the Reddy lab and in my opinion could be taken to another level in an informative manner in this manuscript.

- **From our data, Lamin C and LADs accumulate over time (hours) post mitosis (ie not an off periphery and immediately recruited at the lamina instantaneously) and hence quantifying the time difference especially between lamin A and lamin C and LAD targeting to the nascent lamina is nearly impossible. Additionally, there are cell-cell variations in mitosis duration, and thus timing of mitotic exit, making this absolute measure of time difference less meaningful. Furthermore, as mentioned in the original manuscript and extended with our new results, a significant portion of lamin C remains nucleoplasmic even while a portion gets recruited to the lamina. These considerations, along with a difficulty in scheduling and using live cell imaging instrumentation for the longer imaging times we need, has made it impossible to attempt new experiments directly addressing this particular question**.

**Nonetheless, our data is compelling that lamin C and LAD arrival at the lamina is concurrent at mid to late G1 and that these proceed after lamin A assembly at the NE (that occurs in telophase). The fact that lamins A and C are mostly identical in sequence and almost always assumed as redundant products of the LMNA gene and yet reassembles at the nuclear periphery in different stages of the cell cycle is striking.**

4. How does LAD targeting to the NE take place? It is unclear to me whether a lamin C + LAD complex is targeted "together" to the NE, or whether both an targeted to the NE separately to end up there concomitantly. Addressing this question experimentally would significantly add to our understanding of the establishment of LADs. At the very least, this would be addressed in the discussion.

- **This is the million dollar question (and what our lab focuses on generally). We have speculated a bit about this in the discussion, with the idea that lamin C serves more as a “buffer” on LADs to prevent their mis-aggregation prior to NE association. We do not think laC is necessarily directly recruiting LADsand we added this to the discussion. We now also provide new data that a subset of lamin C remains phosphorylated post mitosis where it remains nucleoplasmic. Additionally, findings from the Ikegami et al that nucleoplasmic lamins bound to euchromatin and particularly at enhancer regions are phosphorylated on Serine 22 suggesting that these interphase interactions are set up during mitosis (which we report). Taken together, these suggest that during mitotic exit, lamin C is excluded from LADs and bound to euchromatin. We surmise that this acts as a mechanism to prevent interchromosomal LAD:LAD aggregation by phase separation or other biophysical means. In addition, lamin C has been shown by Brian Burke’s group to show a tendency towards nuclear pore components. Chromatin underlying the NPCs are also documented to be euchromatic in nature. One hypothesis (speculative) is that lamin C along with its bound euchromatin gets recruited to the periphery via interactions with NPC components (with lamin C later getting incorporated into the lamina meshwork) and as a consequence corrals LADs, that have along the way, intra-chromosomally aggregated via phase separation, to the periphery. In order words, we hypothesize (this is conjecture and the subject of a grant proposal) that lamin C recruitment to the lamina is by protein:protein interactions, perhaps initially with NPC components, mediated by changes in modification state (or vice versa?) whereas LADs are initially condensed via phase-separation via heterochromatin (Karpen lab, Hp1 data) and may then move to the periphery through biophysical means, such as movements driven by and crowding from the active chromatin regions “pushing” the LADs (which are buffered by lamin C) to the lamina meshwork where they are retained these same biophysical constraints along with their interactions with resident INM proteins or adaptor proteins (X.Wong et al LSA 2021)**

5. LADs have been reported to be similar to NADs, based on sequence-based data. In this context, it would be relevant to know whether the nucleoplasmic lamin C+LAD "complexes" are found near nucleoli, with perhaps come even remaining there later in G1.

- **Thanks for pointing this out. LADs and NADs do share some overlap and it has long been known that lamin invaginations to nucleoli do occur. Recent work has identified some NAD-specific regions, so the data in this regard is still fluid.**

**We find that lamin C is excluded from nucleoli, this does not preclude their potential importance in nucleolar biology. This is an area of interest to us as well as how other heterochromatin domains (telomeres/centromeres) also behave relative to lamin C. We did a quick set of experiments to at least address this question, but do not plan on bringing this up in the text since we don't have a definitive answer to the role of lamin C in nucleolar biology (see figure at the end of this document). To justify including these data or speculating on the role of lamin C would require a different set of experiments and avenue of inquiry altogether (ie this would require a repeat of many of the experiments in this manuscript, but focused on nucleoli generally and in the absence of lamin C--and other lamins--are nucleoli behaving differently? Morphologically? Functionally?). We are trying to get funding for just such a set of experiments to understand how lamins and other chromatin/nuclear body compartments interact to “self-organize” into the fully functional mammalian nucleus.**

6. What would be post-mitotic implications of the differential lamin A vs lamin C association with the NE? For example, are there lamin C- and lamin A-specific LADs? The authors suggest from their data that lamin A and C may contribute differently to the organization of chromatin there, a view supported by their references to the structural arrangement of lamins A, C (and B) filaments. Yet, DamID after sh-lamins B1, A, C or A/C show no difference (point 1). Are there nevertheless lamin subtype-specific LADs?

- **This is an excellent point and one that, frankly, we cannot resolve with our existing tools. To get to the kind of organizational details as, one would need an inducible DamID system where protein production/degradation can be induced in a matter of minutes/hours, not days. We are developing more time-resolved DamID tools to address exactly this (and this is part of a now funded grant proposal). We recognise that DamID has been used to profile peripheral chromatin changes during developmental progression or to characterise LAD organization between cell types by various groups including ours, the changes of which are more controlled- when considering a particular locus eg the Igh locus, >90% of cells would have this locus at the nuclear periphery in non-B cells and within the nuclear interior in B cells and hence would be captured by the technique. Random perturbations to LAD organization, however, due to lamina disruptions would be stochastic; DamID data being an aggregate readout from millions of cells, potentially obscure true differences that would be detectable at the level of individual cells^6,9,46^. Therefore, in this study, we focused on imaging tools that unveil (non-averaged single cell level) disturbances with the removal of specific lamin isotypes. We show that only the removal of lamin C (also lamin A/C but shlamin A had no discernable effects) resulted in disruption of overall 3D chromosome organization and peripheral tethering. Interestingly, we also tried to get at the potential of specific LAD/lamin interactions (not within the scope of this paper) with Dam-tagged lamin isotypes. Again, our findings show no significant differences (and this is in agreement with other’s work). It is important to note that the majority of interactions identified in the absence of tightly controlled dam fusions are likely to be interphase configurations, even with the ability to stage cells since the window for current technologies cannot, even given hours to label) suss out these differences. Even with staging of cells, we routinely see 30% or more in G1/S/ or G2 (this is partly because of differential entry into prophase, but much more because different cells transit mitosis at very different rates). Thus, we are developing tools that are more rapidly responsive to cell cycle conditions and that can be easily degraded to get very acute timing of labeling.**

**Nonetheless, we acknowledge that the current DamID method has limitations in terms of spatiotemporal resolution. DamID is a proximity based assay and hence, depending on the labelling radius of the Dam moiety, the use of DamID may be more suited to determine which regions of the genome are in contact with the NE/lamina, which are not and less suited for determining which lamin isoforms are responsible for tethering different genomic regions at the nuclear periphery. Our new DamID tools (once we make them) coupled with the use of super res. microscopy techniques would perhaps be more suited to thoroughly characterise LAD organization and possibly address whether lamin subtype-specific LADs exist, particularly in deficient backgrounds. Additionally, cells are a majority of the time in interphase where most of the DamID methylation and signal would be laid (with mitotic marks potentially obscured).**

7. While the data presented are overall solid, the discussion and model are in my opinion far too speculative. The model is also busy, making it somewhat unclear. For example, there is no evidence from the data that "chromatin loops", or "CTCF" of NPC components or lamin S22P is involved in their observations. Similarly, there are no data supporting a targeting of promoters or enhancers to the NE mediated by lamin A S22P. I also have a scale issue problem with the model, "mid G1 - G1/S" diagram, with an "active chromatin loop" being associated with a nuclear pore in (?) / outside (?) a LAD. At the same time, there some "lamins A, B & C" in a lamina structure.... There is a scale discrepancy between the "loop" and the "LADs" represented. There is, I think, no evidence that chromatin targeting to the NE would be occur at the level of "loops", is there? I can see how some of the ideas are interesting to put forward, but I would refrain from being so speculative in this particular, data-based, manuscript.

- **We have changed the model figure and we generally agree with the reviewer’s comments. We would be delighted to get input on our updated model. To clarify, the model we put forth is a global picture that constitutes data from this manuscript as well as findings from other groups. We will remove most of the “possibilities” for what we labelled as “structural proteins” that may aid lamin C and LAD targeting to the nuclear periphery.**

**It has been recently shown that nucleoplasmic lamin A/C, in interphase, that is bound to euchromatin, specifically at enhancers, is phosphorylated on S22P. We provide additional data in this revised manuscript that a subset of lamin C (and much less of lamin A) remains phosphorylated on S22 upon mitotic exit. This suggests that lamin C enhancer interaction in interphase might be set up at late mitosis-early /mid G1 as we report here (LAD excluded volume).**

**Additionally, super res studies from the Burke group has shown that NPCs preferentially colocalize with the lamin C meshwork, and since we show that lamin C during this re-organization process occupy volumes excluded from LADs, with new data suggesting euchromatin binding, and the environment within the immediate vicinity of NPCs has been shown to be euchromatic, it is not unreasonable that lamin C targeting to NE can be guided by NPCs or mediated by NPCs or an unknown “structural protein”.**

**Furthermore, removal of lamin C in this organizational transition leads to a LAD organization hinting at aberrant intermingling of interchromosomal LADs and that heterochromatin has been shown to aggregate via phase transition. Similarly, our previous work has shown that LADs tend to aggregate prior to their recruitment to the periphery. It is possible that lamin C shepherds the LADs as it reassembles at the periphery via recruitment by NPCs, preserving chromosome compartmentalisation along the way.**

Minor:

Fig 1B, 2B, 5B etc: unclear what the * and P val are related to (through it can be guessed). Please clarify in the legends.

- **Thank you for pointing this out. Sometimes we are so into our own data that we forget to include these kinds of necessary details. (1B is now 1D).**

Reviewer #2

This very interesting manuscript from the Reddy laboratory explores the role that A-type lamins play in defining the peripheral association of LADs. In particular, they demonstrate that the two major A-type lamin isoforms, lamin A and lamin C, are not functionally equivalent. This work represents an important addition to a body of evidence that has been slowly accumulating over the last decade suggesting that lamins A and C may have different, albeit overlapping, roles in defining nuclear organization. The evidence for this runs the gamut from mouse genetics to nanoscale microscopy, both light and EM. The most important take-home message from this manuscript is that is that it is lamin C, not lamin A, that mediates the association of LADs with the nuclear lamina and inner nuclear membrane. This conclusion is based upon a series of knockdown experiments in which they use three independent methods to track LAD distribution, chromosome painting, chromosome insertion of tandem lacO repeats and DamID-based LAD tracer. The question obviously arises as to how these two very closely related proteins can exhibit functional differences in terms of their interactions with LADs. The authors provide intriguing evidence that these differences arise during the late stages of mitosis where lamins A and C exhibit clear differences in behavior. Lamin A appears to be recruited to the nuclear periphery in telophase ahead of lamin C. The latter remains largely nucleoplasmic, as do LADs, in to early G1. The implication is that the later recruitment of lamin C establishes a permissive environment for LAD association with the nuclear envelope. It is also consistent with studies that have suggested that lamins A and C appear to form separate filament networks. This mitosis observation suggests a possible mechanism for the segregations of lamins A and C into separate (or maybe partially separate) networks.

My only concern is with respect to the mitotic studies. I am wary about the use of large N-terminal tags on lamins and the effects that these might have on filament assembly. One way around this would be to use A vs C specific antibodies. Indeed, the authors do take advantage of an antibody "purportedly" specific for lamin C (Fig. 12). This makes me wonder whether the authors have some concerns about the validity of this reagent. It would be nice if they could include a western blot to demonstrate that it is actually C-specific. The alternative approach to this mitotic question would be to employ short tags such as Flag, Myc or HA on the lamin A versus C N-terminus simply to confirm that lamin A is indeed recruited to the NE ahead of lamin C. This could be accomplished quickly on fixed unsynchronized cell populations.

- **As Reviewer 2 suggested, we have confirmed the validity of the lamin C only antibody via western blots (new figure S12). We have also used additional antibodies to all the lamin isotypes and have included this as a complementary panel to the original figure outlining the stages of laA, laB, laC recruitment to the NE. These data confirm the timing of lamin A versus C to the NE.**

**Related to this comment, we have also included data on the timing of lamin C phosphorylation and its relationship to lamin C at these discrete timepoints. These data enhance our understanding why A and C might show this difference. Intriguingly, this dovetails quite nicely with excellent super-res data showing structurally independent networks (from the Burke lab for C vs A). By having a delay in NE recruitment (laB1-->laA--->laC) the networks could remain largely independent even if they were able (in vitro) to form mixed networks.**

Finally, there may be something interesting going on in Supplemental Figure 10b. There are a number of cells in which there is a large amount of nucleoplasmic lamin C. From the Hoechst staining it appears to me that these might be very early prophase cells (I can't be certain of this from the images, but I am very suspicious). If this is the case, then it would appear that lamin C is being released from the lamina ahead of lamin A at the onset of mitosis.

- **We thank reviewer #2 for this very insightful observation. We recognise the possibility that (similar to differential targeting of the lamin isotypes to the nascent NE) the various lamins can dissociate from the lamina at different rates at the onset of mitosis.**

**Firstly, these cells were not synchronised and hence we discriminate easily between cells in specific phases of the cell cycle except the obvious mitotic stages vs “interphase”. That being said, prophase is not a very protracted stage so it would be odd to pick up so many prophase by chance, particularly when laC is dissociated, but not the other lamins, although it is possible. We also do not see a correlation in terms of “prophase like” hoechst staining and levels of nucleoplasmic lamin C. (Comparing similar hoechst stain in row 1 and row 4, yet very different amount of nucleoplasmic lamin C in these cells). We do note that we see this kind of condensed image both in early G1 and in prophase. We will include this in the legend to clarify to the reader.**

**This would be a very interesting question to pursue in future studies. We would love to see this via live cell imaging--indeed one of the movies provided capture the dissolution of laC relative to LADs at prophase.**

In conclusion, this is a well-documented and well-controlled study that should merit publication with only a few minor additions.

**Second round of review**

**Reviewer 1**

The authors have satisfactorily addressed my comments. This ended up being a very nice manuscript.
